# Tumor microenvironment-based screening repurposes drugs targeting cancer stem cells and cancer-associated fibroblasts

**DOI:** 10.7150/thno.62676

**Published:** 2021-09-21

**Authors:** Pei-Jung Lee, Chao-Chi Ho, Hao Ho, Wan-Jiun Chen, Chiu-Hua Lin, Yi-Hua Lai, Yi-Chen Juan, Wen-Chung Chu, Jia-Hua Lee, Sheng-Fang Su, Hsuan-Yu Chen, Jeremy J. W. Chen, Gee-Chen Chang, Ker-Chau Li, Pan-Chyr Yang, Huei-Wen Chen

**Affiliations:** 1 Graduate Institute of Oncology, National Taiwan University College of Medicine, Taipei, 10051, Taiwan.; 2 Graduate Institute of Toxicology, National Taiwan University College of Medicine, Taipei, 10051, Taiwan.; 3 Department of Internal Medicine, National Taiwan University Hospital and National Taiwan University College of Medicine, Taipei, 10051, Taiwan.; 4 Institute of Statistical Science, Academia Sinica, Taipei, 11529, Taiwan.; 5 College of Medicine, China Medical University, Taichung, 40402, Taiwan.; 6 Rheumatic Diseases Research Center, China Medical University Hospital, Taichung, 40402, Taiwan.; 7 Rheumatology and Immunology Center, China Medical University Hospital, Taichung, 40402, Taiwan.; 8 Institute of Biomedical Sciences, National Chung Hsing University, Taichung, 40227, Taiwan.; 9 Health Data Research Center, National Taiwan University, 10050, Taipei, Taiwan.; 10 Chung-Shan Medical University, Taichung, 40201, Taiwan.; 11 Department of Statistics, University of California, Los Angeles, Los Angeles, California, 90095, USA.

**Keywords:** Cancer-associated fibroblasts, cancer stem cells, drug screening, tumor microenvironment, high-throughput

## Abstract

The tumorous niche may drive the plasticity of heterogeneity and cancer stemness, leading to drug resistance and metastasis, which is the main reason of treatment failure in most cancer patients. The aim of this study was to establish a tumor microenvironment (TME)-based screening to identify drugs that can specifically target cancer stem cells (CSCs) and cancer-associated fibroblasts (CAFs) in the TME.

**Methods:** Lung cancer patient-derived cancer cell and CAFs were utilized to mimic the TME and reproduce the stemness properties of CSCs *in vitro* and develop a high-throughput drug screening platform with phenotypical parameters. Limiting dilution assay, sphere-forming and ALDH activity assay were utilized to measure the cancer stemness characteristics. *In vivo* patient-derived xenograft (PDX) models and single-cell RNA sequencing were used to evaluate the mechanisms of the compounds in CSCs and CAFs.

**Results:** The TME-based drug screening platform could comprehensively evaluate the response of cancer cells, CSCs and CAFs to different treatments. Among the 1,524 compounds tested, several drugs were identified to have anti-CAFs, anticancer and anti-CSCs activities. Aloe-emodin and digoxin both show anticancer and anti-CSCs activity *in vitro* and *in vivo*, which was further confirmed in the lung cancer PDX model. The combination of digoxin and chemotherapy improved therapeutic efficacy. The single-cell transcriptomics analysis revealed that digoxin could suppress the CSCs subpopulation in CAFs-cocultured cancer cells and cytokine production in CAFs.

**Conclusions:** The TME-based drug screening platform provides a tool to identify and repurpose compounds targeting cancer cells, CSCs and CAFs, which may accelerate drug development and therapeutic application for lung cancer patients.

## Introduction

Lung cancer remains the leading cause of malignancy-related mortality worldwide 1. Although the identification of driver mutations in non-small cell lung cancer (NSCLC) has helped guide specific targeted therapy for individual patients, drug resistance eventually occurs 2. Growing evidence indicates that cancers are not merely composed of cancer cells; the surrounding tumor microenvironment (TME) and the heterogeneity of tumors also affect tumor progression, drug resistance and metastasis, especially of cancer stem cells (CSCs), which may play a pivotal role 3. The high expression of stemness signature genes is associated with poor clinical outcomes in lung cancer 4, and the therapeutic targeting of CSCs may have promising clinical benefit for cancer patients 5-7.

While efforts have been made to elicit CSCs by targeting their surface markers or signaling cascades, some studies propose that interactions among CSCs, differentiated cancer cells and stromal cells should also be considered 8. When cancer cells were cocultured with stromal cells, the cancer cells showed resistance to chemotherapy 9 and EGFR tyrosine kinase inhibitors in lung cancer cells with EGFR mutations 10. Moreover, differentiated cancer cells can also dedifferentiate into CSCs once the TME is established 11. In particular, cancer-associated fibroblasts (CAFs), which are key components of stromal cells in the TME, can regulate the plasticity of cancer cells 12, 13 and contribute to chemoresistance 14-16. Therefore, strategies targeting both cancer cells and CAFs are urgently needed 17.

However, maintaining the characteristics and plasticity of CSCs and the heterogeneity of cancer cells *in vitro* are difficult. In our previous study, we successfully established a sustainable *in vitro* coculture system using a lung cancer cell and CAFs to reproduce the stemness phenotype of CSCs and a stemness niche supported by CAFs. Additionally, this model could mimic the heterogeneity of the TME 18. Based on this concept, we developed image-based high-content analysis (HCA) screening using a novel coculture system to identify compounds that were effective against CSCs and/or CAFs. This system may facilitate drug discovery and the repurposing of drugs to overcome chemoresistance in cancer patients. We also verified our results and explored the mechanisms using a lung cancer patient-derived xenograft (PDX) model and single-cell RNA sequencing (scRNA-seq).

## Materials and methods

### Cell culture of lung cancer cells, CLS1 cells, and CAFs

Primary cultures of lung cancer cells, CLS1 cells, and CAFs were performed as described by Chen WJ *et al*. 18. Briefly, CLS1 (Table S1) and CAFs were isolated from cancer-associated regions of resected tissues from late-stage NSCLC patients with adenosquamous carcinoma. Two CAFs (CLF1 and 517CAF) were used in this study. CLF1 was derived from the same patient matched with CLS1; the other sample is from another patient (517CAF). We confirmed that both CAFs could upregulate Nanog in CLS1 and that digoxin could inhibit Nanog expression by conducting a western blot analysis (Figure S1A). The CLS1 cells were cultured and maintained in plates preseeded with CAFs (5×10^5^ cells/10 cm dish) at a density of 5,000 viable cells/10 cm dish for a total of 8-10 days (Figure S1B) and cultured in RPMI 1640 medium supplemented with 10% fetal bovine serum (FBS), antibiotics and L-glutamine (2 mM). After colony formation, CLS1 was subcultured as previously described with the following modifications: CLS1 was dissociated by 0.05% trypsin (Invitrogen) for 5 min at 37 °C by picking up the colonies with a 100 μL pipette. The cells were collected by centrifugation (430 × g, 5 min). CLS1 cells confirmed by the single-cell status were seeded at a density of 5,000 viable cells in 10 cm dishes preseeded with CAFs at a density of 5×10^5^ cells.

To compare the stemness markers of CLS1 and cytokines of CAFs under different culture conditions, CLS1 cells were seeded at a density of 5,000 viable cells in 10 cm dishes preseeded with CAFs at a density of 5×10^5^ cells. On the following day, the medium was changed to (1) 10% FBS, (2) EGF (20 ng/mL, Sigma; E9644), bFGF (20 ng/mL, PeproTech; 100-18b) and 1% N2 (Thermo; A1370701), or (3) EGF (20 ng/mL), bFGF (20 ng/mL) and 2% B27 (Thermo; 17504044), and the cells were cultured for a total of 9 days. CD90 bead separation was used to collect the cancer cells and CAFs. The samples were procured and utilized according to approved IRB protocols for research involving human subjects (NTUH IRB201103028RC and IRB201106046RC). Written informed consent was obtained from all patients.

### CD90 bead separation

CD90 magnetic beads were utilized to separate the CAFs from the cancer cells. Briefly, the cocultured cells were detached with trypsin and filtered with a preseparation filter (Miltenyi Biotec, Bergisch-Gladbach, Germany; 30 µm, #130-041-407). After centrifugation, the cells were resuspended in 80 µL of separation buffer (PBS containing 0.5% BSA, 2 mM EDTA, pH=7.2) and 20 µL of CD90 microbeads (Miltenyi Biotec; #130-096-253). After 15 min of refrigeration at 4 °C, 5 mL of separation buffer were added, and the cells were centrifuged at 760 × g for 5 min. The LS column (Miltenyi Biotec; #130-042-401) was attached to the front of the MACS separator and rinsed with 5 mL of separation buffer. The cells were resuspended in 1 mL of separation buffer and added to the LS column. The unlabeled cells (CD90^-^) were collected, and the column was washed three times with 3 mL of separation buffer. The labeled cells (CD90^+^) were collected by the addition of 5 mL of separation buffer, followed by immediate flushing with a plunger. Flow cytometry confirmed that the CAFs from the cocultured CLS1/CAFs showed 99% purity, were CD90-positive (Figure S1C) and expressed α-SMA, whereas the separated CLS1 cells from the cocultured CLS1/CAFs did not express α-SMA, indicating no CAFs contamination (Figure S1D).

### Flow cytometric analysis

CLS1/CAFs coculture cells were separated by CD90 magnetic beads as described above. The CAFs (2×10^5^ cells) were incubated with a CD90-PE antibody (BD; 5196812) or isotype control (BD; 559320) in FACS buffer at 4 °C for 30 min. Then, the cells were washed twice with PBS and analyzed by flow cytometry on an LSRFortessa (Becton Dickinson). The live and dead cells were distinguished by staining with a live/dead fixable dye (Invitrogen; L34976).

### Cell viability assay

To compare the cell viability of CAFs under different culture conditions, CAFs cells were seeded at a density of 5,000 viable cells in 96-well in RPMI 1640 medium supplemented with 10% FBS. 4 h later, cell viability was examined by a Cell Counting Kit-8 (CCK-8) assay (Dojindo Molecular Technologies, CK04-13). 10-fold dilution of CCK-8 was added to cells and incubated for 1.5 h at 37 °C. Then, the absorbance was measured using a multilabel plate reader at 450 nm (Victor3; Perkin-Elmer, USA). The OD value of 4 h was defined as day 0. Next, cells were wash once with PBS and the medium was changed to (1) 10% FBS, (2) EGF (20 ng/mL, Sigma; E9644), bFGF (20 ng/mL, PeproTech; 100-18b) and 1% N2 (Thermo; A1370701), or (3) EGF (20 ng/mL), bFGF (20 ng/mL) and 2% B27 (Thermo; 17504044). Cell viability was measured every day for a total of 7 days. CCK-8 solution was diluted in indicated culture medium.

### Drug screening coculture platform

CAFs were seeded at a density of 2,000 viable cells/96-well and were allowed to attach for 24 h. CLS1 cells were pickup up from the coculture colonies with a 100 μL pipette and digested by 0.05% trypsin (Invitrogen) for 5 min at 37 °C. The CLS1 cells were collected by centrifugation (430 × g, 5 min). The cells were counted by the trypan blue method and seeded at a density of 200 viable cells/96-well for 24 h. The drug treatments were applied for 72 h. Finally, the cells were fixed, and immunofluorescence was conducted (Figure S2A). All compounds were analyzed in triplicate by applying a single dose commonly used in the literature for 72 h. The drugs and doses are summarized in Table S2. The compounds were diluted to a final working concentration of 0.1% DMSO. The compounds included pharmacologically active drugs from the Lopac library (Sigma, St. Louis, MO), natural herbal compounds from ChromaDex (ChromaDex, Irvine, CA) and stem cell signaling inhibitors from Sigma (St. Louis, MO).

### Immunofluorescence

The cells were fixed with 4% paraformaldehyde in PBS at room temperature for 15 min. After washing once with PBS, the cells were permeabilized with permeabilization buffer (0.05% SDS and 0.1% Triton X-100 in PBS, subjected to 0.22-μm syringe filtration) for 15 min. Then, 3% BSA (wt/vol) was used for blocking and hybridization after washing once with PBS. The cells were incubated with monoclonal antibodies targeting Nanog (ReproCELL, Beltsville, MD; 09-0020; 1:300) and CD90 (BD Pharmingen; Clone 5E10; 550402; 1:150) overnight at 4 °C. After washing three times with PBS, the cells were incubated with a tetramethylrhodamine isothiocyanate (TRITC)-conjugated secondary antibody (Thermo Fisher Scientific; A11012; 1:100; goat anti-rabbit IgG (H+L) conjugate) and a fluorescein isothiocyanate (FITC)-conjugated secondary antibody (Thermo Fisher Scientific; A11001; 1:100; goat anti-mouse IgG (H+L) conjugate) for 2 h at room temperature. The nuclei were counterstained with Hoechst 33342 dye (Sigma; 14533; 5 µg/mL) for 15 min at room temperature after washing once with PBS. The stained cells were examined under an Axiovert 200 microscope (Carl Zeiss, Göttingen, Germany) or a confocal laser scanning microscope (C1 si, Nikon, Japan) with MetaXpress (Molecular Devices, Sunnyvale, CA, USA).

### Image analysis

The plates were imaged by ImageXpress Micro XLS Widefield High-Content (Molecular Devices). Four fields per well were captured using a 4× objective. After the image acquisition, a montage image was generated for further segmentation and cell scoring using MetaXpress Custom Module (Version: MetaXpress 6; Molecular Devices). A top hat filter (size=30 pixels, shape=circle) was applied to identify small bright spots based on a filter shape and size from the Nanog images. Then, an open filter (size=6 pixels, shape=circle) was applied to remove parts of the bright areas where the filter shape and size did not fit from the top hat Nanog images. The cell scoring module was utilized to define the positive and negative cells in the sample using user-defined intensity above local threshold methods and maximum and minimum widths. First, the cells that were positively stained with both Hoechst 33342 and Nanog were identified as Nanog^+^ cells. Then, the cells positively stained with both Hoechst 33342 and CD90 were identified as CD90^+^ cells. To identify colonies, the Nanog^+^ and Nanog^-^ cells were expanded by growing object tools (5 pixels) and allowed to touch each other. The grow objects were then filled holes to allow the filled area to be included in the measurement data. Finally, the filled objects were filtered based on (1) an elliptical form factor (ratio of length/breadth) ≤2.5 (to select ellipse or round shaped objects), (2) the area of objects above 10,000 μm^2^ and (3) user-defined minimum intensity standard deviation of Nanog (to remove low intensity objects). A simple threshold was set to help segment the feature from the background. The Nanog^+^ cells within the colony were defined as cancer stem cells, whereas the Nanog^-^ cells within the colony were defined as differentiated cancer cells. The total cells were defined as the sum of Hoechst 33342 positive cells. The total CAFs were defined as the sum of CD90^+^ cells. The total CSCs were defined as the sum of Nanog^+^ cells within the colonies. The total colony cells were defined as the sum of Nanog^+^ and Nanog^-^ cells within the colony. The colony density was defined as the total colony cells divided by the total colony areas, representing the distribution pattern of the colony 19.

### Drug screening data analysis

All compounds were analyzed in triplicate, and the mean of the data was calculated. The mean of the data was used to calculate the percentage change as follows: (Drug-Control)/Control. The percentage change of each drug was normalized by parameter via Blom's method (SAS Institute Inc., Cary, NC). After normalization, a heatmap was generated by hierarchical clustering using the Euclidean distance and centroid linkage via GAP-Lite software.

### 3D- chitosan-hyaluronan spheroid assay

For the berberine experiment, CRL4058-mCherry (300 cells/96-well) was cocultured with CLS1-Nanog-EGFP cells (100 cells/96-well) in chitosan-hyaluronan plates 20. After 24 h, the cells were treated with berberine (1 μM) for 48 h, and Hoechst 33342 (Sigma; 14533; 5 µg/mL) was used to label each cell.

For the digoxin experiment, CAFs (5×10^3^ cells/24-well) were prestained with PKH26 (Sigma; PKH26GL) according to the manufacturer's instructions. Then, PKH26-labeled CAFs were cocultured with CLS1-Nanog-EGFP cells (5×10^3^ cells/24-well). After 24 h, the cells were treated with digoxin (1 nM) for 48 h. Hoechst 33342 was used to label each cell. The stained cells were examined under an Axiovert 200 microscope (Carl Zeiss, Göttingen, Germany) or a confocal laser scanning microscope (C1 si, Nikon, Japan) with MetaXpress (Molecular Devices, Sunnyvale, CA, USA).

### Real-time RT q-PCR

To examine the stemness markers of CLS1 after the aloe-emodin treatment, CLS1 cells (5×10^3^ cells/10 cm dish) were cocultured with CAFs (5×10^5^ cells/10 cm dish) for a total of 7 days. Then, the cocultured cells were treated with or without aloe-emodin (1 μM) for 24 h. Finally, the CLS1 cells were collected by CD90 bead separation for further gene expression assays (Figure S2B). To examine the stemness markers of CLS1 and cytokines of CAFs after the digoxin treatment, CLS1 cells (5×10^3^ cells/10 cm dish) were cocultured with CAFs (5×10^5^ cells/10 cm dish) for a total of 5 days and then treated with or without digoxin (1 nM) for 72 h (Figure S2B). Finally, the CLS1 cells and CAFs were collected by CD90 bead separation for further gene expression assays.

The expression levels of related genes were determined by real-time reverse transcriptase (RT) q-PCR using an ABI Prism 7900 sequencer (Applied Biosystems, Foster City, CA, USA). The primers were designed using Primer Express 3.0 (Applied Biosystems). β-actin and TBP were used as internal controls. The expression levels were normalized to those of the internal controls and defined as ΔCT=[CT_target_-CT_internal control_]. The relative expression ratio was calculated as the fold change relative to the control (2^-∆∆CT^). The primer sequences are listed in Table S3.

### Sphere-forming assay

The sphere-forming assay was conducted as previously described 18 as follows: cells (800 cells/well) were seeded in 24-well ultralow plates (Corning; 3473) in MCDB201 medium (Sigma; M6770) (pH=7.1) supplemented with EGF (Sigma; E9644; 20 ng/mL), bFGF (PeproTech; 100-18b; 20 ng/mL), NaHCO_3_ (Sigma; S5761; 1.2 g/L) and L-glutamine (2 mM); growth factors were added every 2 days, and the spheres were observed after 1-4 weeks.

### Xenograft model

All animal studies were conducted in an Association for Assessment and Accreditation of Laboratory Animal Care, AAALAC International-accredited facility and approved by the Institutional Animal Care and Use Committee (IACUC) at National Taiwan University College of Medicine. To examine CLS1 tumor growth following the aloe-emodin treatment, CLS1 cells (5×10^3^ cells/10 cm dish) were cocultured with CAFs (5×10^5^ cells/10 cm dish) for a total of 7 days. Then, the cocultured cells were treated with or without aloe-emodin (1 μM) for 24 h. The CLS1 cells that formed colonies were picked up with a 100 μL pipette and dissociated by 0.05% trypsin (Invitrogen) for 5 min at 37 °C (Figure S2B). The cells were collected by centrifugation (430 × g, 5 min). The cell numbers were counted by the trypan blue method, and 1,000 viable CLS1 cells (in 100 µL of PBS mixed in Matrigel, BD) were subcutaneously injected into six-week-old SCID mice (LASCO, Taipei, Taiwan).

To investigate the tumor initiation ability of the CLS1 cells after the digoxin treatment, CLS1 cells (5×10^3^ cells/10 cm dish) were cocultured with CAFs (5×10^5^ cells/10 cm dish) for a total of 10 days and CLS1 cells were picked up with a 100 μL pipette and dissociated by 0.05% trypsin (Invitrogen) for 5 min at 37 °C. The cells were collected by centrifugation (430 × g, 5 min). Thirty and 100 viable CLS1 cells (in 100 µL of PBS mixed in Matrigel, BD) were injected into NSG or NSG-SGM3 mice (Jackson Laboratory). On the following day, the mice were randomly separated into two groups and injected intraperitoneally with vehicle control (1% DMSO in normal saline) or digoxin (2 mg/kg/day).

To investigate the tumor growth of primary lung cancer cells following digoxin treatment (Table S1), 6.5×10^3^ to 10^4^ viable primary lung cancer cells or ALDH^+^ lung cancer cells were subcutaneously injected into NSG or NSG-SGM3 mice (Jackson Laboratory). H&E staining confirmed that the histology of the patient tissues and xenograft model were similar (Figure S3A). On the following day, the mice were randomly assigned to receive vehicle control (1% DMSO in normal saline) or digoxin (2 mg/kg/day) intraperitoneally.

For the PDX model (Table S1), the tumor was cut into 1 mm^3^ pieces and implanted into the flanks of NSG mice. On the following day, the mice were randomly assigned to receive vehicle control (1% DMSO in normal saline) or digoxin (2 mg/kg/day) intraperitoneally. H&E staining confirmed that the histology of the patient tissues and xenograft model were similar (Figure S3B). All mice were monitored three times per week for 2-10 weeks, and the tumor volumes and body weights were recorded. The mice were sacrificed if the tumor volume exceeded 1 cm^3^ or body weight loss was greater than 20%.

To examine the tumor growth of CLS1 cells by digoxin in combination with chemotherapy, CLS1 cells (5×10^3^ cells/10 cm dish) were cocultured with CAFs (5×10^5^ cells/10 cm dish) for a total of 10 days and CLS1 cells were picked up with a 100 μL pipette and dissociated by 0.05% trypsin (Invitrogen) for 5 min at 37 °C. In total, 1,000 viable CLS1 cells (in 100 µL of PBS mixed in Matrigel, BD) were subcutaneously injected into six-week-old SCID mice (LASCO, Taipei, Taiwan). After the tumor volume reached approximately 30 mm^3^, the mice were randomly assigned to receive vehicle control, digoxin (2 mg/kg/day), cisplatin (3 mg/kg, twice a week) or paclitaxel (10 mg/kg, twice a week). The mice were monitored for 3 weeks, and the tumor volume and body weight were examined. The tumor volume was monitored three times per week by electronic Vernier calipers and calculated using the formula volume=0.4×*ab*^2^, where *a* and *b* are the longest and shortest diameters of the tumors, respectively.

### Western blotting

CLS1 cells (5×10^3^ cells/10 cm dish) were cocultured with CAFs (5×10^5^ cells/10 cm dish) for a total of 5 days and then treated with or without digoxin (1 or 10 nM) for 72 h. On the following day, the CLS1 and CAFs cells were collected by CD90 bead separation for western blot assay (Figure S2B). The primary antibodies against Nanog (#4903S; 1:1,000) and α-SMA (#14968; 1:1,000) were purchased from Cell Signaling Technology; anti-β-actin (MAB1501; 1:5,000) was purchased from Millipore. The membranes were washed three times with TBST and incubated with horseradish peroxidase (HRP)-conjugated secondary antibody (Millipore, 1:2,000) in 5% skim milk in TBST. The bound antibody was detected using an enhanced chemiluminescence system (Millipore, Burlington, MA). The chemiluminescent signals were captured using a Fujifilm LAS 3000 system (Fujifilm, Tokyo, Japan). An image J software was used to quantify protein bands. Full-length images of the immunoblots are shown in Figure S4-5.

### Limiting dilution assay (LDA)

*In vitro* LDA was performed as previously described with modifications as follows 21, 22: CLS1 cells (5×10^3^ cells/10 cm dish) were cocultured with CAFs (5×10^5^ cells/10 cm dish) for a total of 5 days and then treated with or without digoxin (1 nM) for 72 h. The CLS1 cells and CAFs were collected by CD90 bead separation (Figure S2B). Then, 1-500 CLS1 cells/well were seeded in 96-well ultralow plates (Corning; 3474) in MCDB201 medium supplemented with EGF (20 ng/mL), bFGF (20 ng/mL), NaHCO_3_ (1.2 g/L), L-glutamine (2 mM), sodium pyruvate (1 mM), heparin (Sigma; H3149; 4 mg/mL) and 2% B27 (Thermo; 17504044) (hereafter referred to as MCDB complete medium); growth factors were added every 2 days. To evaluate the CSC frequency of the xenograft tumors, the tumors were digested, the viable tumor cells were determined by the trypan blue method, and tumor cells were seeded at 1-1,000 cells/well into ultralow 96-well plates in MCDB complete medium. The CSC frequency was calculated by an extreme limiting dilution assay as previously reported 23.

### Tumor digestion

Tumors derived from the xenograft model were washed with PBS three times. Collagenase I (1 mg/mL) and deoxyribonuclease I (1 mg/mL) in serum-free RPMI 1640 supplemented with PSA (digestion buffer) were used for the tumor digestion. First, 1 mL of digestion buffer was added to a 10 cm dish, and the tumors were cut into pieces smaller than 1 mm^3^ by surgical scissors. Then, the tumor pieces were collected by 9 mL of digestion buffer into a 50 mL tube, incubated at 37 °C and shaken every 10 min three times. The tumors were filtered by a strainer and washed with 10 mL of RPMI 1640 supplemented with 10% FBS, antibiotics and L-glutamine (complete medium). The cells were harvested at 430 × g for 5 min and washed three times with complete medium. RBC lysis buffer (155 mM NH_4_Cl, 12 mM NaHCO_3_ and 0.1 mM EDTA) was used to remove red blood cells. The cells were resuspended in MCDB complete medium for the limiting dilution assay.

### Aldefluor assay

Cellular ALDH activity was measured using an Aldefluor Assay Kit (StemCell Technologies, Vancouver, Canada; #01700) following the manufacturer's protocol. Briefly, the cells were suspended in Aldefluor assay buffer containing the ALDH substrate BODIPY aminoacetaldehyde (1 mM per 1×10^6^ cells, incubated for 30 min at 37 °C). As a negative control, a fraction of the cell sample was treated with diethylaminobenzaldehyde, a specific ALDH inhibitor. An LSRFortessa instrument (Becton-Dickinson, Franklin Lakes, New Jersey, USA) was used for the analysis.

### MTT assay

Cells were seeded in 96-well plates at a density of 3,000-5,000 cells/well depending on the type of cell. After 24 h, the cells were treated with different concentrations of the indicated drugs and incubated for 72 h. Then, 0.5 mg/mL MTT ([3-(4,5-dimethylthiazolyl-2)-2,5-diphenyltetrazolium bromide]) (Sigma; M2128) solution was prepared and added to the culture medium in the wells. After 1.5 h of incubation at 37 °C, the medium was removed, and 100 µL of DMSO was added to each well. The colorimetric intensity was measured at 570 nm using a multilabel plate reader (Victor3; Perkin-Elmer, USA). The IC_50_ was determined with GraphPad software (La Jolla, California, USA) according to the manufacturer's instructions. To determine the IC_50_ of the drugs in CSCs, the total CSCs derived from the HCA data were calculated.

### Lung cancer cells

Primary lung cancer cell lines (CL1-0, CLB1, CLH8, CLH16, CL100, CL83, CL25, CL141, CL152, and CLY1) (Table S1) were procured and utilized according to approved IRB protocols for research involving human subjects (IRB201705055RINC and SF18106A). Written informed consent was obtained from all patients. The BEAS-2B cells and CRL4058 cells were obtained from American Type Culture Collection (ATCC; Manassas, VA, USA). PC-9 was a kind gift from Dr. Chih-Hsin Yang (National Taiwan University Hospital, Taiwan). The CL1-0, CLB1, CLH8, CLH16, CL100, CL83, CL25, CL141, CL152 and PC-9 cells were cultured in RPMI 1640 supplemented with 10% FBS, antibiotics and L-glutamine, while 5 ng/mL EGF was added to the CLH8 culture medium. The cells were grown at 37 °C under a humidified atmosphere consisting of 20% O_2_ and 5% CO_2_. CL1-5 cells were cultured in DMEM supplemented with 10% FBS, antibiotics and L-glutamine. CLY1 cells were cultured in F medium as previously reported 24.

### Population-based nested case-control study

#### Study population

The data sources for this analysis were the Taiwan National Health Insurance (NHI) 25 database and the Taiwan Cancer Registry 26. The study cohort consisted of all patients with an incident diagnosis of CHF and/or AF between January 1, 2005, and December 31, 2012, with follow-up through December 31, 2013. All cases of incident lung cancer in the cohort were ascertained from the Taiwan Cancer Registry. Among a cohort of 651,830 patients with CHF/AF in Taiwan (2005~2012), 6,928 cases of incident lung cancer and 69,267 matched controls were identified (Figure S6A). The baseline characteristics of the cases and controls showed no significant difference within 1 year prior to the diagnosis of CHF/AF (Table S4). Incidence density sampling among the CHF/AF cohort was used to identify up to 10 controls for each incident lung cancer case, matched by sex, year of birth (+/-1 year), and year of first CHF/AF diagnosis (+/-1 year). The index date was defined as the date of diagnosis of lung cancer in the Taiwan Cancer Registry, and the same date was assigned to the matched controls for each case. All digoxin prescriptions from the date of first CHF/AF diagnosis through the index date for all cases and controls were identified. The usage of digoxin before cohort entry (first CHF/AF incidence) was not included. Based on the assumption that recent exposure to digoxin would not have a substantial impact on the lung cancer diagnosis and because cancer initiation occurred before the lung cancer diagnosis, digoxin use during the 90 days before the index date was not considered in the case-control analysis.

#### Digoxin exposure duration and accumulative prescription day definition

The digoxin exposure duration was defined as the period between the first to the last day of digoxin prescription (duration between A_1_+A_2_+A_3_…+A_n_ in Figure S6B) and was 1.87 months in the cases and 2 months in the controls on average (Table S5). The cumulative prescription days of digoxin were defined as the sum of digoxin from the medical orders.

#### Statistical analysis

The strength of the association between digoxin use and incident lung cancer was evaluated by a conditional logistic regression and is presented as the crude and adjusted odds ratios (ORs) and 95% confidence intervals (CIs). Income and comorbidities were included as covariates in the multiple regression model. The potential dose-response relationship between digoxin and lung cancer risk was evaluated among subjects with different cumulative days of digoxin prescriptions, excluding digoxin use within 90 days prior to the index date. The data management and analysis were conducted with SAS version 9.4 (SAS Institute Inc., Cary, NC), and the data were obtained and processed in accordance with the guidelines of the Health and Welfare Data Science Center (HWDC), Ministry of Health and Welfare (MOHW). This study was reviewed and approved by the ethics committee of National Taiwan University Hospital (NTUH approval number: 201512065 W).

### Immunohistochemical staining

The sections were deparaffinized and then rehydrated. Then, the sections were incubated in antigen retrieval buffer (10 mM sodium citrate, 0.05% Tween 20; pH=6) at 121 °C for 10 min. Subsequently, the samples were treated with 3% H_2_O_2_ at room temperature for 15 min. Then, the slides were incubated with a polyclonal anti-Nanog antibody (ReproCELL, 09-0020; 1:100) or an anti-α-SMA antibody (Sigma, A5228; 1:500) overnight at 4 °C. The staining was visualized using diaminobenzidine chromogen (Thermo, TA-125-QHDX), followed by counterstaining with hematoxylin (Leica; 3801570). An IHC Profiler was used to quantify the nuclear expression of Nanog according to the instruction manual 27. Samples with scores of 2 (positive) and 3 (highly positive) were considered Nanog^+^ populations.

### Single-cell RNA-sequencing

CLS1 cells (5×10^3^ cells/10 cm dish) were cocultured with CAFs (5×10^5^ cells/10 cm dish) for 5 days and then treated with or without digoxin (1 nM) for 72 h. The CLS1/CAFs coculture mixture cells were collected by trypsin. The cells were suspended in 100 μL of Dead Cell Removal MicroBeads (Miltenyi Biotec; 130-090-101) and incubated for 15 min at room temperature. The separation column (Miltenyi Biotec; 130-042-201) was rinsed with 3 mL of separation buffer (PBS containing 0.5% BSA, 2 mM EDTA, pH=7.2). Then, a cell suspension was applied to the column, and the live cells were collected by washing four times with 3 mL of separation buffer. Live single cells were loaded on a Chromium Single Cell Instrument (10× Genomics, Pleasanton, CA), and cDNA amplification and library construction steps were performed according to the manufacturer's instructions. The single-cell libraries were sequenced using the Illumina NextSeq 500 system (parameters: paired-end sequencing with dual indexing, 26 cycles for Read 1, 8 cycles for I7 Index Read and 98 cycles for Read 2).

#### Preprocessing for each single-cell RNA-seq library

For each library, the reads were mapped to the prebuilt human reference transcriptome GRCh38-3.0.0 provided by 10x Genomics and used to generate the raw count matrix using Cell Ranger version 3.1.0. Cells with UMI counts less than one-tenth of the 99th percentile or more than 15% derived from the mitochondrial genome were filtered. Doublets were annotated by the R package scds 28 with a cutoff of the hybrid score greater than the outer fence. For the remaining singlets, the expression matrix was normalized using the R package scran 29. To reduce the dimensionality, highly variable genes were selected using scran with a cutoff of an FDR<0.01 from testing the null hypothesis that the biological component of the variance was no greater than 0. The first 50 principal components of the highly variable genes were computed and used to generate two-dimensional UMAP embedding using Monocle3 30. The Leiden community detection algorithm was performed to cluster cells into different cell types 31. Small clusters with median numbers of expressed genes fewer than 3,000 were considered low-quality cells and removed.

#### Cell-type-specific single-cell RNA-seq data analysis

The raw count matrices of the cells from the two libraries were merged together for each of the following two cell types: CLS1 and CAF. The same normalization and highly variable gene detection procedures were applied to the merged count matrix of each cell type. The cell cycle scores of the S and G2M phases were estimated by the CellCycleScoring function in Seurat 32. For the dimensional reduction step, the highly variable genes were first summarized by the first 50 principal components. To remove the effects of technical and unrelated biological factors, the cell cycle scores, mitochondrial read percentage, UMI counts and number of features expressed were regressed out from the top 50 PCs using the function align_cds in Monocle3 30. The aligned PCs were used to generate two-dimensional UMAP embedding.

The top genes that varied over UMAP were identified by the graph test function in Monocle3 30 with cutoffs of Q<0.01 and Moran's I>0.1. Then, the selected genes were grouped into modules using the find_gene_modules function in Monocle3 30. For each module, a score was computed by summing all gene expression within the module and scaled to unit-variance. The module scores were used to cluster the cells by hierarchical clustering, where the optimal number of clusters was determined by the average silhouette score. The marker genes in each cluster were identified by the top_markers function in Monocle3 30 with cutoffs of Q<0.01 and cluster specificity>0.2. A two-sided Wilcoxon rank sum test was performed to compare the control and digoxin groups using the function FindMarkers in Seurat 32.

### Cytokine secretion

CLS1 cells (5×10^3^ cells/10 cm dish) were cocultured with CAFs (5×10^5^ cells/10 cm dish) for a total of 5 days and then treated with or without digoxin (10 nM) for 72 h. On the following day, cells were washed once gently with PBS and incubated in RPMI serum free medium for 24 h. The cell culture supernatant were then centrifuged at 430 × g for 5 min at 4 °C, and cytokines were validated by ProcartaPlex multiplex immunoassay (Thermo Fisher Scientific).

### Statistical analysis

Quantitative *in vitro* and *in vivo* data are presented as the mean±s.e.m. unless otherwise noted. Statistical significance was assessed using two-tailed Student's t-test for pairwise comparisons of groups. One-way analysis of variance with Tukey's post hoc correlations was used for comparisons of multiple groups. All tests were two tailed, and P-values≤0.05 (*P-values≤0.05, ** P-values≤0.01, ***P-values≤0.001) were considered significant.

## Results

### Image-based high-content TME-directed drug screening platform

An *in vitro* coculture system comprising CAFs and a population of lung cancer cells can mimic the TME of patient and maintain stemness of cancer cells 18. The cancer cells (CLS1 cells) cocultured with primary cultured CAFs grew robustly, formed tumorous colonies with high tumorigenicity, and maintained cancer stemness characteristics as determined by cancer stemness markers (Nanog, Sox2, Oct3/4, KLF4, ALDH1A1 and CD44) (Figure S7) 18. Since culture medium with serum has been reported to promote the differentiation of CSCs 33, we further examined the CLS1/CAFs coculture system in different culture media (Figure S8A). The stemness marker of the CLS1 cells was enriched when cocultured with CAFs under 10% FBS culture conditions and was comparable to that observed under serum-free culture conditions (Figure S8B). On the other hand, it is crucial for the survival and maintenance of the growth of CAFs in serum-containing media (Figure S8C). CAFs might provide a niche maintaining cancer stemness by secreting inhibitory factors against cell differentiation, e.g., IGF-II, CXCL12, IL6 and IL8 14, 18, 34 (Figure S8D). These data demonstrate that this coculture system could enrich the stemness of CLS1 cells and cytokines of CAFs in serum-containing culture medium.

**Figure 1:**
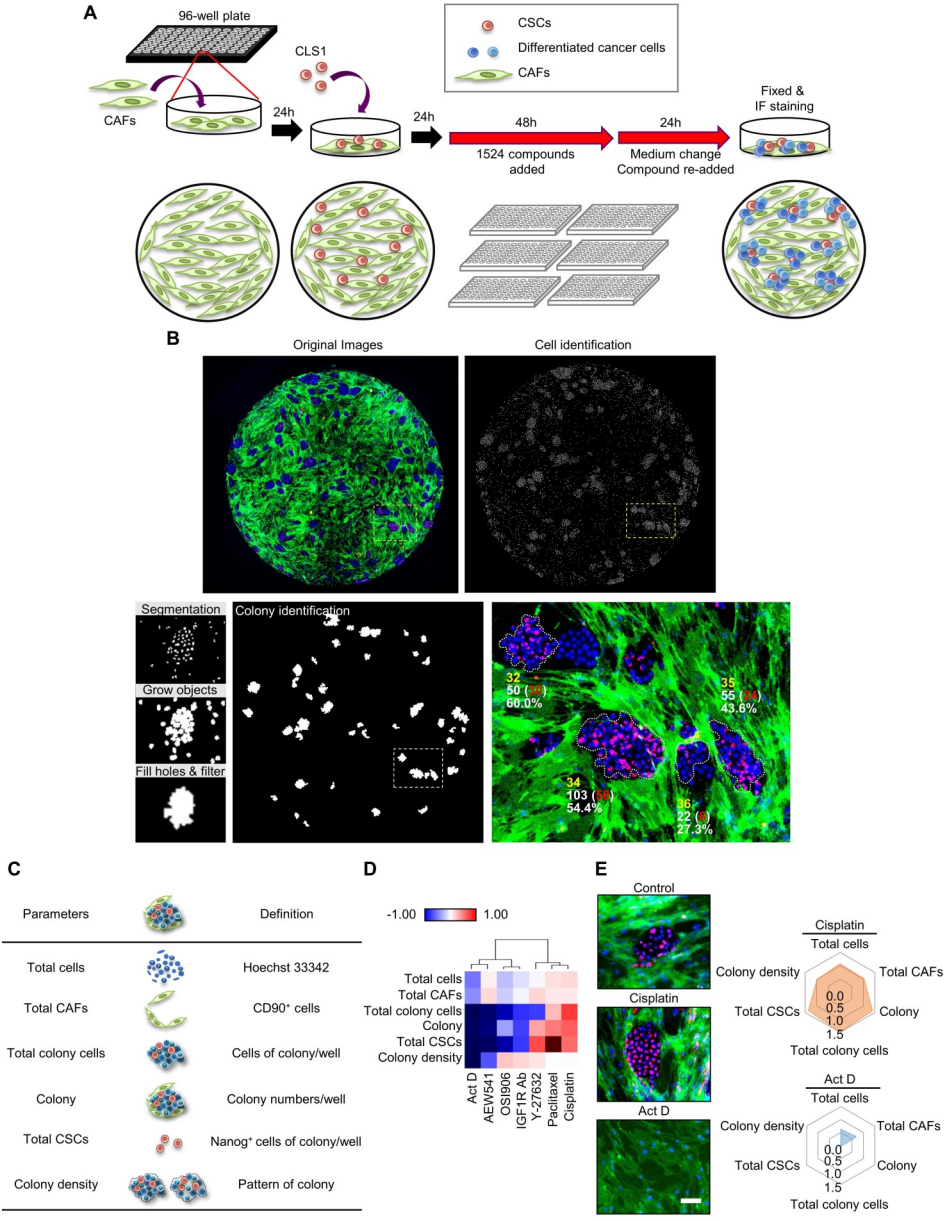
** The TME-based CSCs/CAFs coculture system serves as a drug screening platform. (A)** Schematic depiction of the CLS1/CAFs coculture drug screening protocol. **(B)** Cell segmentation and colony identification by image-based HCA (MetaXpress 6). **(C)** Six parameters were used to validate the efficacy of drugs in the CLS1/CAFs coculture screening platform. **(D)** Unsupervised hierarchical clustering of mean factor scores for the training set results. The data are presented as the mean of three replicates normalized to the control from CLS1/CAFs coculture screening platform data by HCA (Act D: 10 nM, AEW541: 1 nM, OSI906: 10 nM, IGF1R Ab: 1 μg/mL, Y-27632: 1 nM, paclitaxel: 10 nM, cisplatin: 100 nM). **(E)** Images and radar chart showing the six parameters of actinomycin D and cisplatin. The data are presented as the mean of three replicates normalized to the control from CLS1/CAFs coculture screening platform data by HCA (Act D: 10 nM, cisplatin: 100 nM, scale: 100 μm). Act D: actinomycin D; HCA: high-content analysis, IGF1R Ab: IGF1R neutralizing antibody. Results were repeated at least three times.

Therefore, an image-based high-content TME-directed drug screening platform comprising CLS1 cells and CAFs was established (Figure 1A). Since Nanog expression in the CLS1 cells was significantly increased when cocultured with CAFs (Figure S7), Nanog served as the CSC marker of CLS1 cells. CD90 exhibited CAF-specific staining (Figure S1C) 35 and was used to identify the numbers of CAFs. Hoechst 33342 was used to define the nuclei and the location of the nucleus as a parameter used to determine the total cell numbers. The plates were imaged by an HCA platform using a 4× objective. Four fields per well were captured at each wavelength, and a montage was created for further analysis. Subsequently, the cells were segmented for the cell scoring. The cells that were positively stained with both Hoechst 33342 and Nanog were defined as Nanog^+^ cells. The cells that were positively stained with both Hoechst 33342 and CD90 were defined as CD90^+^ cells. To identify colonies, the Nanog^+^ and Nanog^-^ cells were expanded by growing objects, and the cells were allowed to touch each other. Then, cell cluster masks comprising grew-Nanog^+^ and Nanog^-^ cells were created, and were filled holes to allow the filled area to be included in the measurement data. The cell cluster masks with low intensity of Nanog would be removed and cluster masks that formed ellipse or circle shape and the area greater than 10,000 μm^2^ were defined as colonies (Figure 1B) 18. To quantify the effects of the compounds on the niche, six parameters were designed to profile the compound-responsive phenotypes (Figure 1C). The total cell count referred to cells with positive Hoechst 33342 staining. The total CAFs were positively stained with CD90 and Hoechst 33342. The colony indicates the number of cells that formed colonies. The total CSCs were defined as the sum of Nanog^+^ cells in each colony, and the total colony cells were defined as the sum of cancer cells (Nanog^-^ and Nanog^+^ cells) in each colony. The colony density was defined as the total colony cells divided by the total colony areas and represents the distribution pattern of the colony 19. The image-based high-content TME-based drug screening platform determined whether the candidate compounds were anti-CSCs drugs (because a reduced level of cancer stemness parameters was observed) or anti-CAFs drugs (because a reduced level of total CAFs was observed).

To validate the platform, we initially trained our system with known compounds (Figure 1D). First, we used cisplatin, paclitaxel and Y-27632, which have been reported to promote the growth of the CSCs (Nanog^+^ cell) population. In addition, OSI906, an IGF1R neutralizing antibody and actinomycin D have been shown to inhibit CSCs characteristics 18. In our platform, cisplatin had the ability to enrich the CSC population, whereas actinomycin D significantly inhibited CSCs (Figure 1E). These data are consistent with the results of previous reports 18, 36-38 and demonstrate the robustness of our system. Therefore, we applied this platform to large-scale high-throughput drug screening.

### TME-based high-content screening identified compounds with anti-CSCs and/or anti CAFs properties

**Figure 2:**
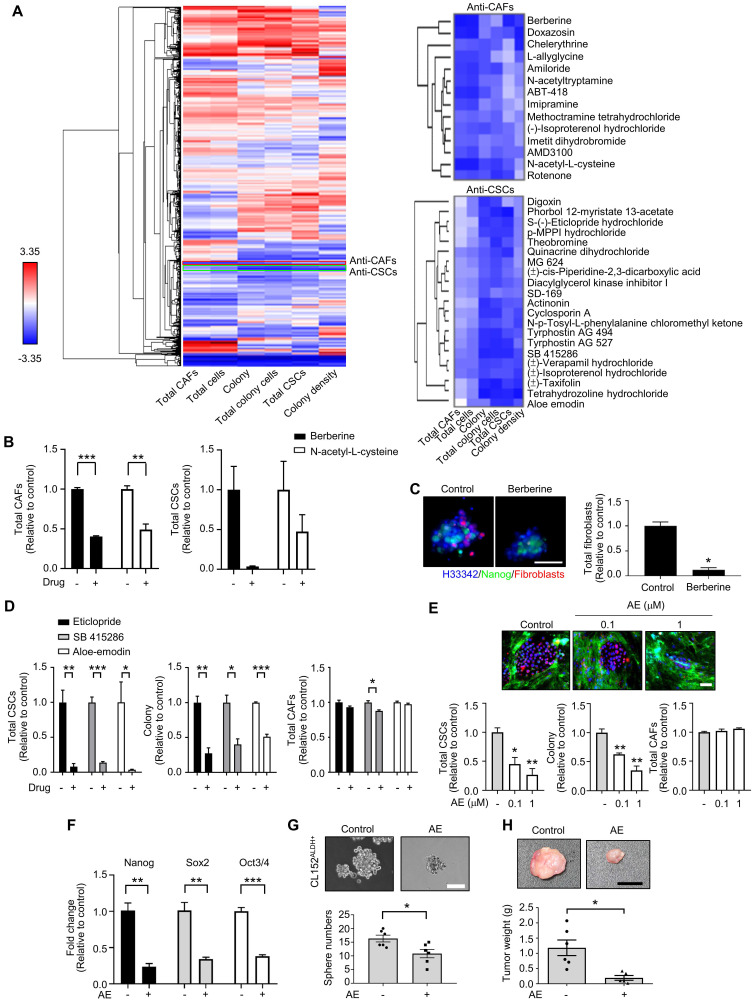
** High-throughput drug screening identifies anti-CAFs and anti-CSCs drugs. (A)** All compounds were analyzed in triplicate by applying a single dose commonly used in the literature for 72 h. Unsupervised hierarchical clustering of the mean factor scores of each of the 1,524 hits. Clustering was based on centroid-linkage criteria and the Euclidean distance metric. **(B)** Total CAFs and total CSCs of anti-CAFs clustering compounds from CLS1/CAFs coculture screening platform data by HCA (N=3, 1 μM). **(C)** Total fibroblasts in CLS1/CAFs 3D coculture in chitosan-HA plates treated with berberine (1 μM) for 48 h (Scale: 100 μm) (N=3). **(D)** Total CSCs, colonies and total CAFs of anti-CSCs clustering compounds from coculture screening platform data by HCA (N=3, 1 μM). **(E)** Total CSCs, colonies and total CAFs of aloe-emodin from coculture screening platform data by HCA (N=3, scale: 100 μm). **(F)** CLS1/CAFs coculture cells treated with aloe-emodin (1 μM, 72 h) and the gene expression of stemness markers in CLS1 cells were examined by RT-qPCR (N=3). **(G)** Sphere numbers of 1,000 viable CL152^ALDH+^ cells treated with aloe-emodin for 2 weeks (N=3, scale: 100 μm). **(H)** CLS1/CAFs coculture cells treated with aloe-emodin (1 μM, 72 h) and CLS1 cells were subcutaneously injected into SCID mice. Tumor weight was recorded (N=6 for control; N=5 for aloe-emodin). The data represent the mean±s.e.m. (B-H). Differences were assessed using Student's t-test. Results were repeated at least two-three times (B-G). AE: aloe-emodin.

Based on the CLS1/CAFs coculture drug screening data of 1,524 compounds, the mean of triplicate data was calculated. Then, the percentage change ((Drug-Control)/Control) of each drug was calculated and normalized by parameter via Blom's method. The compounds could be profiled into different clusters by hierarchical clustering using the Euclidean distance and centroid linkage (Figure 2A). To identify the compounds targeting CAFs, clusters that showed inhibitory effects on total CAFs were selected. Furthermore, to avoid nonselective cytotoxicity, clusters that inhibited >80% of the total CSCs, colony and total colony cells were excluded. We identified one cluster, i.e., berberine and N-acetyl-L-cysteine, which showed anti-CAFs effects (Figure 2B). In addition, berberine showed a tendency to reduce the total CSCs, but the difference did not reach statistical significance (p=0.08) (Figure 2B). Berberine and N-acetyl-L-cysteine have also been reported to regulate fibroblasts 39, 40. We further examined the anti-CAFs activity of berberine in a 3D coculture model 20 comprising CLS1 cells and fibroblasts. Berberine decreased the number of fibroblasts in the 3D culture system, which is consistent with the data from the drug screening coculture (Figure 2C).

Additionally, to identify anti-CSCs compounds, clusters that exhibited inhibitory effects on colony, total colony cells, total CSCs and colony density were selected. Then, CAFs were used to validate the direct cytotoxicity of the compounds and determine whether the compounds show nonselective cytotoxicity or is specific to CSCs/cancer cells. Therefore, we identified one cluster that showed inhibitory effects on colony, total colony cells, total CSCs and colony density but fewer cytotoxic effects on CAFs (less than 15% on average). Several candidates from the chosen cluster, including aloe-emodin, eticlopride and SB 415286 (Figure 2D), have been reported to inhibit cancer stemness 41-43. Aloe-emodin was confirmed to significantly inhibit the Nanog^+^ CSC population and reduce the colony numbers by the CLS1/CAFs drug screening coculture platform (Figure 2E). The gene expression of stemness markers (Nanog, Sox2 and Oct3/4) in the CLS1 cells was also downregulated by the aloe-emodin treatment (Figure 2F). Aloe-emodin decreased the sphere numbers of CL152^ALDH+^ cells, exhibiting anti-CSCs properties *in vitro* (Figure 2G). Aloe-emodin also exhibited a tumor reduction of 84% *in vivo* (Figure 2H). Collectively, the CLS1/CAFs drug screening coculture platform could identify potential candidates with anti-CAFs properties, such as berberine, and/or anti-CSCs properties, such as aloe-emodin, which exhibits anti-CSCs activity and antitumor effects.

### TME-based screening of repurposed cardiac glycoside digoxin with anti-CSCs properties

**Figure 3:**
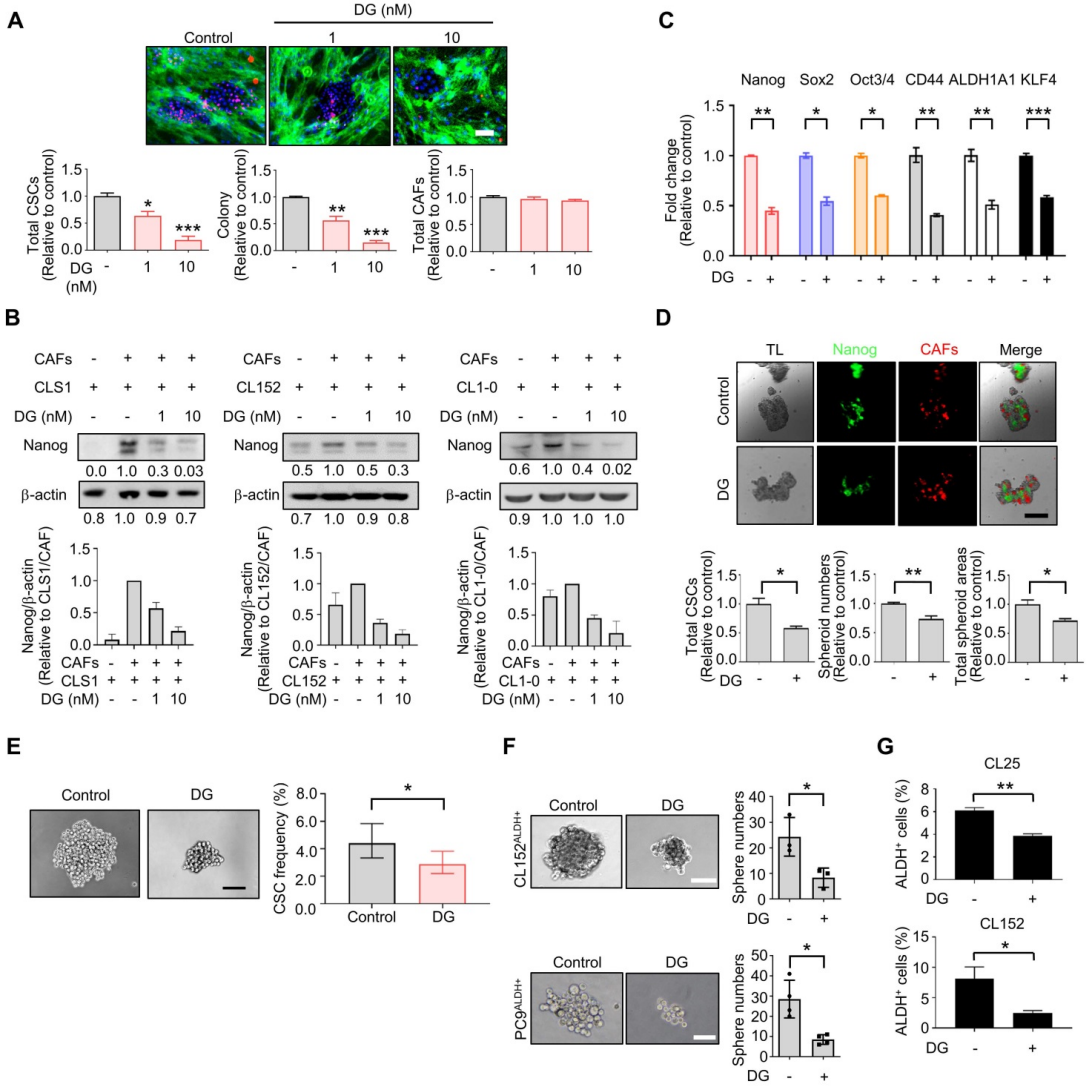
** The tumor niche drug screening platform identifies digoxin with anti-CSCs activity. (A)** Total CSCs, colonies and total CAFs of digoxin (72 h) from coculture screening platform data by HCA (N=3, scale: 100 μm). **(B)** CLS1, CL152 and CL1-0 cells were cocultured with CAFs treated with digoxin for 72 h. Nanog expression of CLS1, CL152 and CL1-0 was examined by a western blot analysis. The bar chart represents the quantification of western blots of biological replicates (N=3 for CLS1, CL152; N=2 for CL1-0). **(C)** CLS1/CAFs coculture cells treated with digoxin (1 nM, 72 h) and the gene expression of stemness markers in CLS1 cells were examined by RT-qPCR (N=3). **(D)** CLS1 Nanog-EGFP reporter cells cocultured with CAFs and formed 3D spheroids on chitosan-HA plates. Total CSCs (Nanog reporter expression), spheroid numbers and total spheroid areas after treatment with digoxin (1 nM, 48 h) were examined (Scale: 100 μm) (N=3). **(E)** CLS1 cells were cocultured with CAFs treated with digoxin (1 nM, 72 h) *in vitro,* and the CD90 magnetic bead separation method was used to collect the CLS1 cells. The CSC frequency of CLS1 was determined by a limiting dilution assay (N number as summarized in Table S6, scale: 100 μm). **(F)** Sphere numbers of CL152^ALDH+^ and PC9^ALDH+^ cells treated with digoxin (1 nM, 2 weeks) (Scale: 100 μm). **(G)** ALDH activity in CL25 and CL152 cells treated with digoxin (1 nM, 72 h) (N=3). The data represent the mean±s.e.m. (A, B, C, D, F, G); mean±95% CI (E). Differences were assessed using Student's t-test (A, C, D, F, G); extreme limiting dilution assay (E). Results were repeated at least two-three times. AE: aloe-emodin; DG: digoxin; TL: transmitted light.

Among the anti-CSCs clusters, cardiac glycoside digoxin exhibited anti-CSCs and anticancer potency at concentrations as low as 1.0 to 10 nM, which mimicked the dose used in clinical practice (0.8 to 2 ng/ml, equal to 1 nM-2.6 nM) 44. Digoxin inhibited the Nanog^+^ CSC population and colony number, and the survival of CAFs was only slightly affected by the CLS1/CAFs drug screening coculture platform (Figure 3A). The inhibition of the Nanog^+^ CSC population and colony number were also observed to a lesser extent in CL152 cells cocultured with CAFs from the HCA data (Figure S9A). The inhibition of Nanog protein expression was confirmed by a western blot analysis of the CLS1 cells and other primary lung cancer cell lines (CL152 and CL1-0) cocultured with CAFs treated with digoxin (Figure 3B). In addition, the gene expression of cancer stemness markers was downregulated in CLS1 cells treated with digoxin (Figure 3C). We also utilized a CLS1/CAFs 3D coculture model to verify this 2D coculture drug screening platform. Digoxin inhibited the spheroid numbers and Nanog^+^ cells of CLS1 (Figure 3D). The CSC frequency was determined by a canonical limiting dilution assay (LDA) to examine whether digoxin affects the CSC population. Digoxin significantly decreased the CSC frequency of CLS1 *in vitro* (Figure 3E and Table S6). The sphere-forming ability was utilized to examine the anti-CSCs effects of digoxin in other primary lung cancer cells by seeding a low density of cells in a nonadherent plate. Digoxin inhibited the sphere numbers of CL152^ALDH+^ and PC9^ALDH+^ cells (Figure 3F). ALDH activity was also inhibited by digoxin, showing anti-CSCs properties in different primary lung cancer cell lines (Figure 3G). To validate the specificity and selectivity of digoxin for CSCs, the IC_50_ was examined by using CLS1 cells and CL152^ALDH+^ cells cocultured with CAFs or in the absence of CAFs (differentiated CLS1 and CL152 cells). Digoxin showed specificity to CSCs and had no significant cytotoxic effects on stromal cells under the therapeutic dosage (Table S7). In summary, our established image-based high-content TME-directed drug screening platform could screen anti-CSCs candidates, such as repurposed digoxin *in vitro.*

### Digoxin inhibited CSCs *in vivo* and suppressed tumor growth in a PDX model

**Figure 4:**
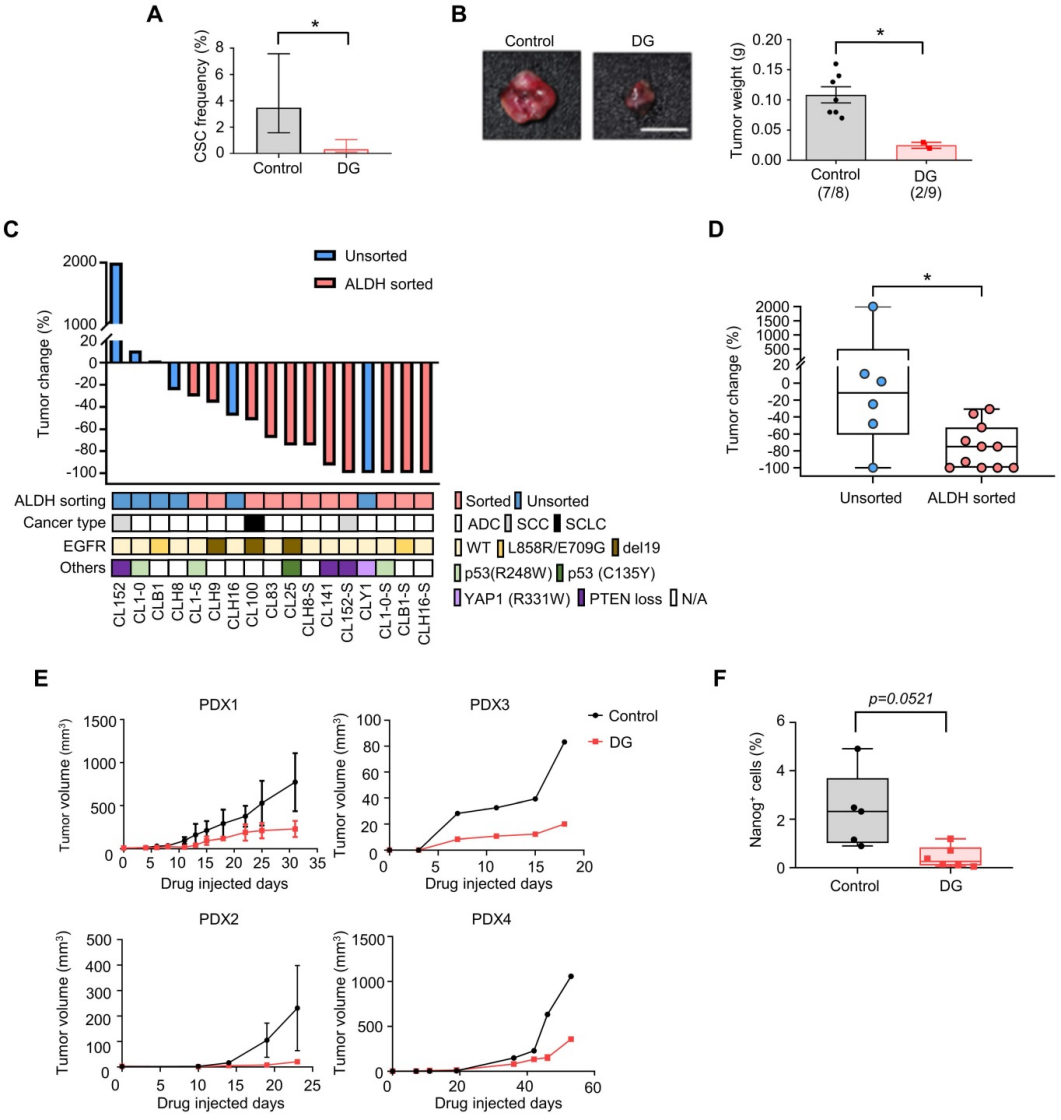
** Digoxin inhibits CSCs of CLS1 *in vivo* and suppresses tumor growth in a PDX model. (A)** CSC frequency of CLS1 with or without digoxin treatment *in vivo* by a limiting dilution assay (N number is summarized in Table S8). **(B)** Tumor weight of CLS1 with or without digoxin *in vivo* (N=8 for control; N=9 for digoxin, 100 cells/mouse, scale: 1 cm). **(C)** Waterfall plot showing the percentage of tumor weight change in the digoxin-treated tumors compared to that in the control tumors *in vivo*. The underlined box summarizes the background and characteristics of each primary lung cancer cell line (ALDH, ALDH^+^ population with and without sorting, pink: with sorting, blue: without sorting; cancer type, ADC: adenocarcinoma; SCC: squamous cell carcinoma; SCLC: small cell lung cancer; EGFR, mutation status of EGFR; Others: other mutation in each primary lung cancer cell, N/A: not available). N number of each cell line: CL152 (control=1, DG=1); CL1-0 (control=2, DG=2); CLB1 (control=1, DG=1); CL1-5 (control=1, DG=1); CLH9 (control=1, DG=1); CLH16 (control=2, DG=2); CL100 (control=1, DG=1); CL83 (control=1, DG=1); CL25 (control=1, DG=1); CLH8^ALDH+^ (control=1, DG=1); CL141 (control=1, DG=1); CL152^ALDH+^ (control=4, DG=5); CLY1 (control=1, DG=1); CL1-0^ALDH+^ (control=1, DG=1); CLB1^ALDH+^ (control=1, DG=1); CLH16^ALDH+^ (control=1, DG=1). **(D)** Tumor weight change in the ALDH-sorted and unsorted groups. **(E)** Tumor growth in each PDX model with or without digoxin (2 mg/kg/day) (N number of each PDX: control=3, DG=4 for PDX1; control=2, DG=1 for PDX2; control=1, DG=1 for PDX3; control=1, DG=2 for PDX4). **(F)** Quantification of Nanog^+^ cells from IHC staining of the PDX1 model. The data represent the mean±95% CI (A); mean±s.e.m. (B, E); minimum to maximum with all points (D, F); differences were assessed using an extreme limiting dilution assay (A); Student's t-test (B, F); Mann-Whitney test (D). Results were repeated at least two times (A, B, F). DG: digoxin; PDX: patient-derived xenograft.

To further confirm the anti-CSCs effects of digoxin* in vivo*, a limiting dilution assay was used to measure the CSCs frequency of CLS1 after the digoxin treatment *in vivo*. CAF-cocultured CLS1 cells were subcutaneously implanted into immunodeficient mice. On the following day, digoxin was administered to examine the tumor initiation ability. Digoxin significantly decreased the CSCs in CLS1 by 10.5-fold (Figure 4A and Table S8) and the tumor weight (Figure 4B and Figure S9B) without severe side effects (Figure S9C-D). In addition to the CLS1 cells, we further sorted the ALDH^+^ population of CL152 cells to represent CSCs to examine the anti-CSC effects of digoxin. Digoxin was administered 1 day after the tumor implantation. Tumors occurred in the control group in 4 of 4 injected hosts, whereas the digoxin-treated group did not form tumors (0 of 5 hosts; Figure S9E). Hence, digoxin inhibited the tumor initiation ability of CLS1 and CL152^ALDH+^ cells *in vivo*. We also examined the antitumor effects of digoxin on other patient-derived primary cancer cell-generated xenograft models. Digoxin was administered 1 day after the tumor implantation. Interestingly, digoxin showed an inhibitory trend toward the ALDH-sorted cells as determined by the tumor weight change (Figure 4C). The tumor weight change was significantly different, showing that digoxin predominantly inhibits tumor growth in the ALDH-sorted group (Figure 4D).

Finally, we validated the antitumor effects of digoxin in a PDX model, which was established as a standardized anti-CSCs validation model. The PDX model presented histology comparable to that of lung cancer patients (Figure S3B). The microenvironmental heterogeneity of tumor cells and stromal cells (CAFs with positive staining for the myofibroblast marker α-SMA was comparable to our* in vitro* model (Figure S9F). As CSCs play a critical role in tumor initiation, digoxin was administered 1 day after the tumor implantation. Digoxin significantly inhibited tumor growth in different PDX models at a clinically comparable dose (Figure 4E) 45, and the subpopulation of Nanog-positive cells was reduced (Figure 4F). Since CSCs are crucial for the initiation of cancer 46, our data suggest that digoxin could have the potential to inhibit tumor initiation. Interestingly, epidemiological research based on health insurance data may support our observation as a potential benefit in terms of a lower odds ratio of lung cancer was observed in the digoxin usage group in the Taiwan National Health Insurance data (Table S9). In conclusion, these data show that digoxin exhibits anti-CSCs properties *in vivo* and inhibits tumor growth in a PDX model, demonstrating the feasibility of this drug screening platform.

### Digoxin in combination with chemotherapy inhibits CSC subpopulation and tumor growth *in vivo*

**Figure 5:**
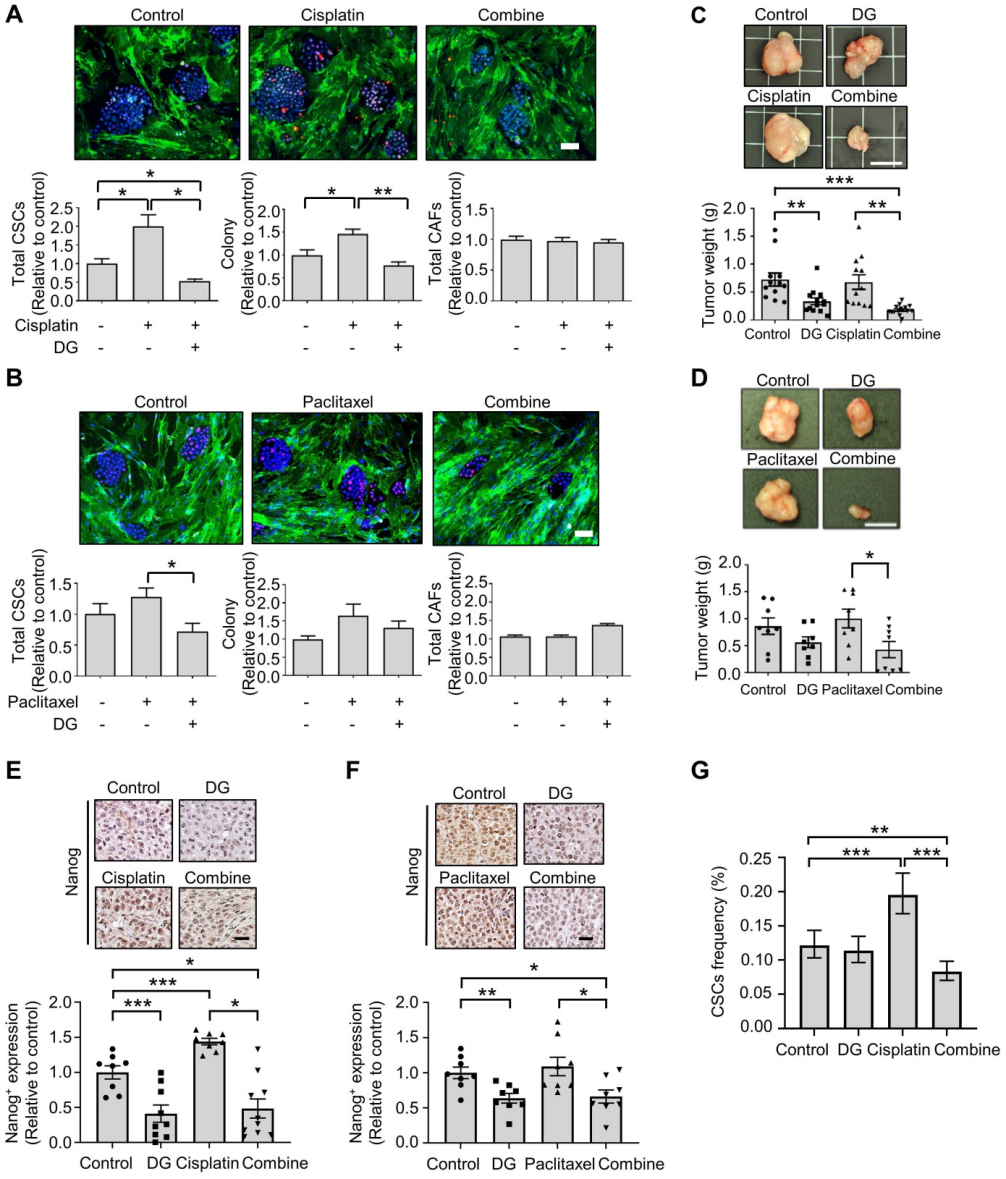
** Digoxin in combination with chemotherapy shows antitumor effects and inhibits CSC subpopulation. (A)** Total CSCs, colony and total CAFs of cisplatin (100 nM), digoxin (1 nM) or combination from coculture screening platform data by HCA (72 h, N=3, scale: 100 μm). **(B)** Total CSCs, colony and total CAFs of paclitaxel (10 nM), digoxin (1 nM) or combination from coculture screening platform data by HCA (72 h, N=3, scale: 100 μm). **(C)** CLS1 cells were cocultured with CAFs for 10 days, and the CLS1 cells were picked up. In total, 1,000 cells/mouse were subcutaneously injected into NOD-SCID mice, and the mice were treated with digoxin (2 mg/kg/day), cisplatin (3 mg/kg, twice a week) or their combination. Tumor weight was recorded (scale: 1 cm). **(D)** CLS1 cells were cocultured with CAFs for 10 days, and the CLS1 cells were picked up. In total, 1,000 cells/mouse were subcutaneously injected into NOD-SCID mice, and the mice were treated with digoxin (2 mg/kg/day), paclitaxel (10 mg/kg, twice a week) or their combination. Tumor weight was recorded (scale: 1 cm). **(E-F)** IHC staining and quantification of Nanog expression in xenograft tumor sections from Figure 5C and 5D; (E) cisplatin and (F) paclitaxel (scale: 20 μm). **(G)** Tumors derived from Figure 5C were digested, and the CSC frequency of the corresponding tumor cells was measured by a limiting dilution assay (N number is summarized in Table S10). The data represent the mean±s.e.m., (A-F); mean±95% CI (G); differences were tested by one-way analysis of variance (ANOVA); an extreme limiting dilution assay (G). Results were repeated at least two-three times (A-F). DG: digoxin.

Accumulating evidence indicates that cytotoxic chemotherapeutic treatments can “reawaken” quiescent CSCs, leading to cell division, repopulation of residual tumors and resistance to chemotherapy 47. We hypothesized that if chemotherapy-“activated” CSC populations are critical for drug resistance, this activation could be abrogated by cotreatment with the anti-CSCs candidate compound digoxin. According to the Nanog expression in the CLS1 cells determined by HCA, digoxin exhibited inhibitory effects on the chemotherapy drug-induced CSC population (Figure 5A-B). Despite the resistance to chemotherapy and enrichment of the CSC population of CLS1 cells based on previous findings 18 and the above data, digoxin exhibited antitumor growth effects with cisplatin in the CLS1 xenograft model (Figure 5C). Digoxin in combination with paclitaxel also led to the inhibition of tumor growth compared to the paclitaxel treatment alone (Figure 5D). Furthermore, tumors treated with chemotherapy exhibited increased Nanog^+^ populations, while digoxin alone and the combined treatment led to the inhibition of Nanog expression (Figure 5E-F). Finally, the CSC frequency of the CLS1 tumors from the xenograft model was evaluated. Cisplatin increased the CSC frequency, while digoxin in combination with cisplatin inhibited the CSC frequency (Figure 5G and Table S10). Collectively, digoxin in combination with chemotherapy could inhibit the CSC subpopulation and tumor growth and might show the potential of digoxin as an anticancer therapeutic adjuvant.

### Cardiac glycoside digoxin had dual inhibitory effects on CSCs and CAFs

**Figure 6:**
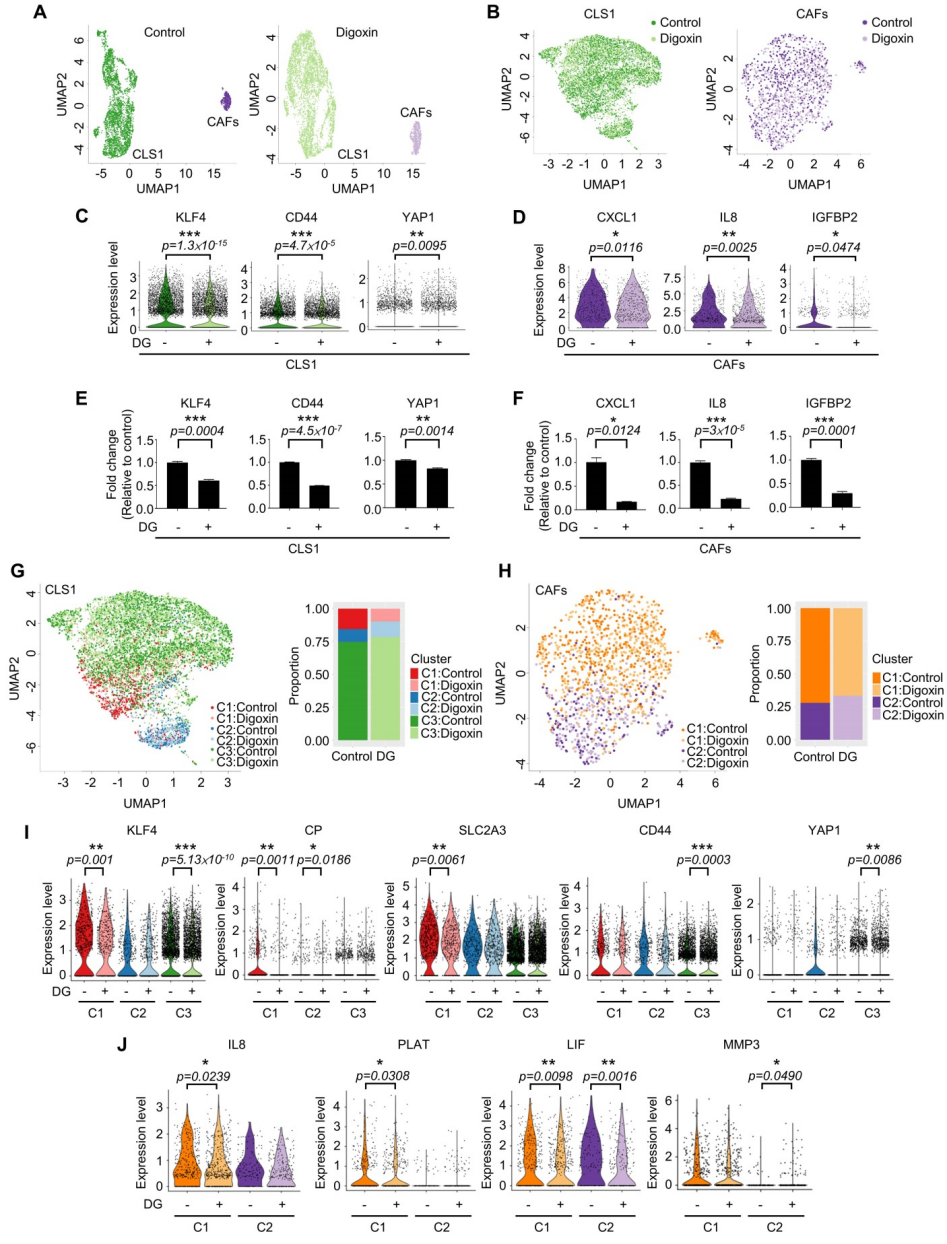
** Single-cell RNA sequencing reveals dual effects of digoxin on CSCs and CAFs. (A)** UMAP embeddings of the cocultured CLS1 and CAF cells from the control and digoxin (1 nM, 72 h) libraries separately. A total of 3,830 CLS1 cells and 543 CAFs from a library obtained under control conditions and 4,304 CLS1 cells and 724 CAFs from a library obtained under digoxin conditions (1 nM, 72 h). **(B)** UMAP embeddings showed that the cells merged from the two libraries of CLS1 and CAFs. **(C)** Overall expression of stemness-related genes in CLS1 cells with or without digoxin (1 nM, 72 h). **(D)** Overall expression of cytokines in CAFs with or without digoxin (1 nM, 72 h). **(E)** Gene expression of stemness markers in CLS1 cells with or without digoxin by RT-qPCR (1 nM, 72 h; N=3). **(F)** Gene expression of cytokines in CAFs with or without digoxin by RT-qPCR (1 nM, 72 h; N=3). **(G)** UMAP embeddings showing the cell clusters by module scores, and the bar charts show the population composition of CLS1 cells. **(H)** UMAP embeddings showing the cell clusters by module scores, and the bar charts show the population composition of CAFs. **(I)** Stemness-related genes in different clusters of CLS1 cells with or without digoxin (1 nM, 72 h). **(J)** Cytokine genes in different clusters of CAFs with or without digoxin (1 nM, 72 h). The bars represent the mean±s.e.m. and the differences were assessed using a two-sided Wilcoxon rank sum test (C, D, I, J) and Student's t-test (E, F). DG: digoxin.

Due to the heterogeneity of cancer cells and the complicated interaction between cancer and stromal cells, we used a single-cell RNA sequencing (scRNA-Seq, 10X Genomics) approach to determine whether this platform could truly identify candidates targeting the CSC population at the cellular level as demonstrated by digoxin. Unsupervised clustering analysis revealed two populations of CLS1 and CAFs (Figure 6A). Then, we merged the cells from the two libraries by each cell type (Figure 6B). Overall, the downregulation of CSC markers, including CD44, KLF4 and YAP1 48-51, was observed in CLS1 cells treated with digoxin (Figure 6C). Furthermore, the overall scRNA-seq data revealed the downregulation of cytokine genes, including CXCL1, IL8 and IGFBP2, in the CAFs treated with digoxin (Figure 6D). IL8 and IGFBP2 are also critical for inducing stemness in lung cancer and glioma, respectively 14, 52. We further confirmed that the gene expression of CSC markers was downregulated in the CLS1 cells treated with digoxin (Figure 6E), while the gene expression and secretion of cytokines were inhibited in the CAFs treated with digoxin (Figure 6F and Figure S10A). Subsequently, based on the coexpressed genes that varied between the cells in UMAP, the cells sharing a similar pattern of module scores were grouped (Figure 6G-H). We found that the number of CLS1 cells in the C1 cluster was significantly reduced from 15.6% to 9.8% (p-value=3.339×10^-15^), and, in C1, several differentially expressed genes that have been proposed to regulate CSCs, including KLF4, ceruloplasmin (CP) and solute carrier family 2 member 3 (SLC2A3) 53, 54, were downregulated after treatment with digoxin as confirmed by RT q-PCR (Figure 6I, 3C and S10B). Digoxin also inhibited the gene expression of CD44 and YAP1 of the cells in the C3 cluster (Figure 6I). Regarding CAFs, several CSC-supporting cytokine genes were also downregulated in the control versus digoxin comparison, including IL8 14, tissue-type plasminogen activator (PLAT) 55 and leukemia inhibitory factor (LIF) 56 in the C1 cluster. In the C2 cluster, matrix metallopeptidase 3 (MMP3), which has been reported to promote the epithelial-mesenchymal transition of lung cancer 57, was inhibited by digoxin (Figure 6J and Figure S10C). In summary, the scRNA-seq data further support that the CLS1/CAFs drug screening platform could indeed identify candidates targeting the CSC population. The scRNA-seq data also repurposes digoxin at a clinically applicable dose with dual effects on CSCs and CAFs.

## Discussion

In this study, by using a CLS1/CAFs coculture and high-content analysis, we established a TME-based high-throughput drug screening system. This TME-based drug screening platform enabled us to comprehensively evaluate the response of cancer cells, CSCs and CAFs to different treatments. We identified compounds with anti-CAFs, anticancer and anti-CSCs activities. Aloe-emodin and digoxin are two examples that showed both anticancer and anti-CSCs activity *in vitro* and *in vivo*. We further confirmed that the combination of digoxin and chemotherapy could improve the therapeutic efficacy in a PDX model.

Although numerous efforts are dedicated to oncology, the rate of the successful development of new drugs in this field is comparatively lower than that for other diseases. One factor is the lack of an appropriate preclinical model 58. The heterogeneity of tumor and the complicated interaction between cancer cells and stromal cells are difficult to mimic *in vitro*. Especially for CSCs, the difficulty of isolating CSCs and maintaining cancer stemness properties *in vitro* and considering the effects of the TME hinder drug development 59. Here, we proposed a TME-based drug screening model comprising CSCs, differentiated cancer cells and CAFs to mimic the TME and examine the effect of the microenvironment on cancer cells and CSCs. This model could simultaneously screen candidates with anti-CSCs potential and the additional benefit by testing the effects on cancer and stromal cells from a single well by image-based high-content analysis with specific phenotypical parameters. The novel TME-based drug screening platform provided a new method to identify candidates via multiple evaluations, including pharmacological and toxicological phenotypes and molecular events. To date, the conversion of differentiated cancer cells into CSCs by the addition of cytokines is widely used to screen anti-CSCs drugs. However, cell-cell interactions between cancer and stromal cells are lacking and the defined cytokines might not truly recapitulate the TME. A 3D culture model that recapitulates the TME in patients is also adapted for drug screening but is currently confined to low throughput. Moreover, cell viability is widely used to evaluate drug effects, which might discover drugs targeting proliferative cells and, consequently, enrich the CSCs population 59. We proposed the TME-based drug screening model to compensate for the insufficiency of the previous model. Molecular events coupled with phenotypical parameters enable us to identify specific drugs. This platform could be used to study the interplay among CSCs, differentiated cancer cells and stromal cells and identify drugs targeting different populations in the niche. The TME-based drug screening platform could also be applied to stroma-rich cancers, such as pancreatic cancer and breast cancer, and is not limited to lung cancer (data not shown).

Among the 1,524 pharmacological drugs and natural products screened, we observed that berberine and N-acetyl-L-cysteine have potential anti-CAFs activity. Berberine has been reported to inhibit angiotensin II-induced collagens and cytokines in cardiac fibroblasts, while N-acetyl-L-cysteine has been reported to regulate cytokines by downregulating the PI3K/AKT signaling pathway in cancer cell-cocultured CAFs 39, 40. Furthermore, we identified potential anti-CSCs candidates, and some targeting signals have been reported 41-43. Eticlopride was identified to reduce the total CSCs and colony numbers and is a dopamine antagonist found to selectively target leukemia stem cells and lung CSCs via antagonism of D2-family dopamine receptors and apoptotic mechanisms 43. Anthraquinone-based compounds have been found to inhibit CSCs and reduce CD44 variant 6 expression in melanoma stem-like cells 41. Here, we identified that aloe-emodin could significantly inhibit CSCs in our model. We also found that digoxin inhibited CSCs at a therapeutic dosage (1-10 nM) 44, which was almost a hundred times lower than that previously reported in the inhibition of various cancers 60, 61. The anti-CSCs activity of digoxin was also examined *in vivo* by measuring the CSCs frequency of CLS1 and injecting ALDH^+^ primary lung cancer cells. We also observed that the antitumor effects of digoxin show a preference for ALDH-sorted cells compared to unsorted cells. It might be more appropriate to compare the effects of digoxin in ALDH^+^ cells with differentiated cancer cells (ALDH^-^ cells). However, the sorted differentiated cancer cells (ALDH^-^ cells) displayed a low tumor formation ability *in vivo* 18; thus, using these cells might be difficult and limited to examining the effects of digoxin. We also repurposed digoxin to exhibit both anti-CAFs and anti-CSCs activity at a clinically relevant dose of 1 nM, which might fulfill currently unmet needs of targeting cancer and stroma cells simultaneously 17. Collectively, these data demonstrate that the TME-based platform was able to identify drugs specifically targeting CSCs or CAFs. The potential anti-CSCs candidates could be further confirmed *in vitro* and *in vivo*, demonstrating the feasibility and translatability of this platform.

The intrinsic parameters of cancer and extrinsic stimulation of stromal cells in the TME contribute to the response to therapy, and approaches considering cancer and stroma are urgently needed 17. For example, the presence of CAFs is correlated with a poor prognosis, and CAF-secreted cytokines (IL-6 and IL-8) confer drug resistance in lung cancer 14. After treatment with a combination of docetaxel and sonidegib targeting breast cancer and CAFs, one patient experienced complement remission, and 3 of 12 patients showed benefits in the EDALINE clinical trial 62. These studies highlight the importance of dual targeting and should be considered during drug development. Our TME-based drug screening platform could compensate for this clinically unmet need. We proposed a new therapeutic strategy by demonstrating that dual-targeting CSCs and stromal cells could inhibit the CSC subpopulation as confirmed by scRNA-seq data. Further detailed mechanisms are worthy of investigation to clarify the interplay between CSCs and CAFs and identify new therapeutic vulnerabilities.

In conclusion, we established a niche-based CSCs/CAFs drug screening platform to mimic the TME of cancer patients and identify compounds that can specifically target CSCs or CAFs. We demonstrated that the platform could identify compounds with translatability; for example, aloe-emodin and digoxin inhibited the expression of stemness markers, sphere-forming ability and tumor growth in combination with chemotherapy and a PDX model. This model has great potential in facilitating drug discovery and repurposing drugs that can overcome chemoresistance in lung cancer patients.

## Supplementary Material

Supplementary figures and tables.
