# Supplementary material for: Tumor microenvironment-based screening repurposes drugs targeting cancer stem cells and cancer-associated fibroblasts
Source: Theranostics. 2021 Sep 21;11(19):9667–86. doi: 10.7150/thno.62676 (PMC8490509; doi:10.7150/thno.62676)
Supplement: Supplementary file 1 — Supplementary figures and tables. [file thnov11p9667s1.pdf]

**Supplementary Materials**

# Tumor micorenviroment-based screening repurposes drugs targeting cancer stem cells and cancer-associated fibroblasts

Pei-Jung Lee,[1,2] Chao-Chi Ho,[3,#] Hao Ho,[4,#] Wan-Jiun Chen,[1,#] Chiu-Hua Lin,[2] Yi-Hua Lai,[5,6,7] Yi-Chen Juan,[8] Wen-Chung Chu,[2] Jia-Hua Lee,[2,4] Sheng-Fang Su,[1] Hsuan-Yu Chen,[4] Jeremy J. W. Chen,[7] Gee-Chen Chang,[9] Ker-Chau Li,[4,10] Pan-Chyr Yang[1,3,*] and Huei-Wen Chen[2,*]

*Corresponding authors: Pan-Chyr Yang & Huei-Wen Chen

Email: pcyang@ntu.edu.tw & shwchen@ntu.edu.tw

## Supplementary Information

### The association between digoxin usage and lung cancer

Both *in vitro* and *in vivo* data support that digoxin can significantly inhibit CSCs using a therapeutic dose (1 nM). Since digoxin has been used for decades, we sought to evaluate the association between digoxin treatment and lung cancer. Although previous evidence-based on early observations of cardiac patients being treated with digoxin has shown that digoxin can act as an anticancer drug and may reduce the incidence of cancer, recent epidemiological studies have shown that digoxin could be responsible for increasing or reducing cancer risk, and this effect remains controversial for different types of cancers [1-3]**.** Here, we conducted a nested case-control study using data from the Taiwan National Health Insurance (NHI) data and the Taiwan Cancer Registry among incident atrial fibrillation (AF) and/or congestive heart failure (CHF) patients. Among a cohort of 651,830 patients with CHF/AF in Taiwan (2005~2012), 6,928 cases of incident lung cancer and 69,267 matched controls were identified (Figure S6A). The baseline characteristics of the cases and controls showed no significant difference within 1 year prior to the diagnosis of CHF/AF (Table S4). As shown in Table S9, the odds ratio (OR) of digoxin use was 0.92 (95% confidence interval (CI): 0.86-0.99). Furthermore, the adjusted OR was 0.89 (95% CI: 0.83-0.96) when adjusted for income and comorbidities. Interestingly, the adjusted OR was 0.93 (95% CI: 0.84-1.03) for those who received less than 30 days of cumulative days of digoxin treatment, 0.87 (95% CI: 0.76-1.00) for those who received 30-89 days of cumulative days of digoxin treatment, and 0.87 (95% CI: 0.79-0.97) for those who received digoxin more than 90 days of cumulative days of digoxin treatment. Collectively, the NHI database showed that a lower odds ratio was observed in the digoxin usage group, while confounding factors (e.g., smoking and family history) were not taken into consideration due to limitations of the database.

**Supplementary References**

1. Biggar RJ, Wohlfahrt J, Oudin A, Hjuler T, Melbye M. Digoxin use and the risk of breast cancer in women. J Clin Oncol. 2011; 29: 2165-70.

2. Boursi B, Haynes K, Mamtani R, Yang YX. Digoxin use and the risk for colorectal cancer. Pharmacoepidemiol Drug Saf. 2014; 23: 1147-53.

3. Osman MH, Farrag E, Selim M, Osman MS, Hasanine A, Selim A. Cardiac glycosides use and the risk and mortality of cancer; systematic review and meta-analysis of observational studies. PloS one. 2017; 12: e0178611.

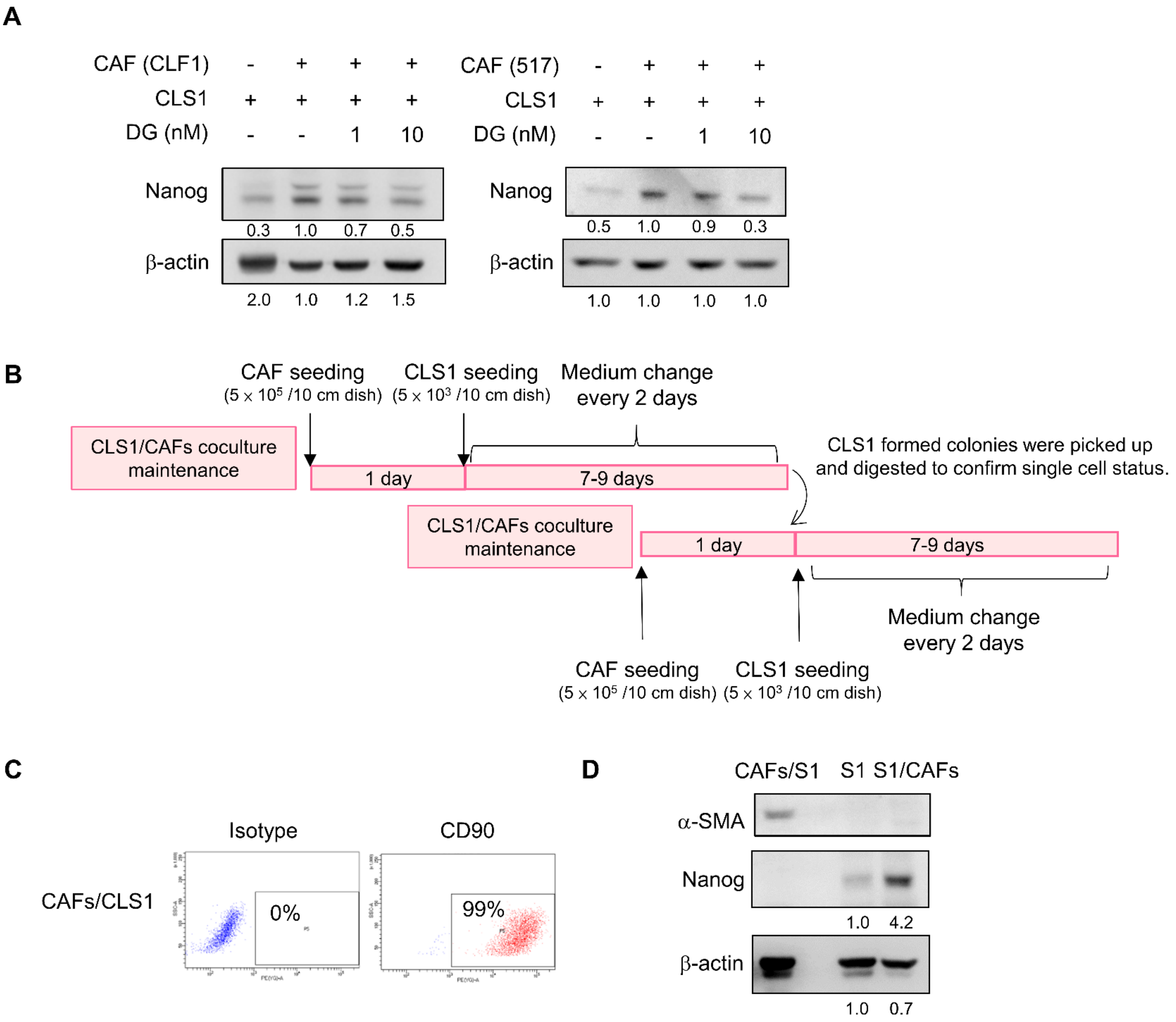


**Figure S1. Timeline of CLS1/CAFs coculture model and validation of CD90 separation method.**

(A) The Nanog expression of CLS1 when cocultured with different CAFs (left: CLF1; right: 517CAF).

(B) Timeline of CLS1/CAFs coculture maintenance.

(C) Flow cytometry analysis showed the CD90-positive ratio of the cells isolated from CLS1-cocultured CAFs (N=2).

(D) The protein levels of α-SMA (CAFs marker) and Nanog (cancer stemness marker) in CAFs/S1 (CAFs separated from CLS1 cocultured), S1 (CLS1 cells without cocultured), and S1/CAFs (CLS1 cells separated from CAFs cocultured) (N=2). DG: digoxin.

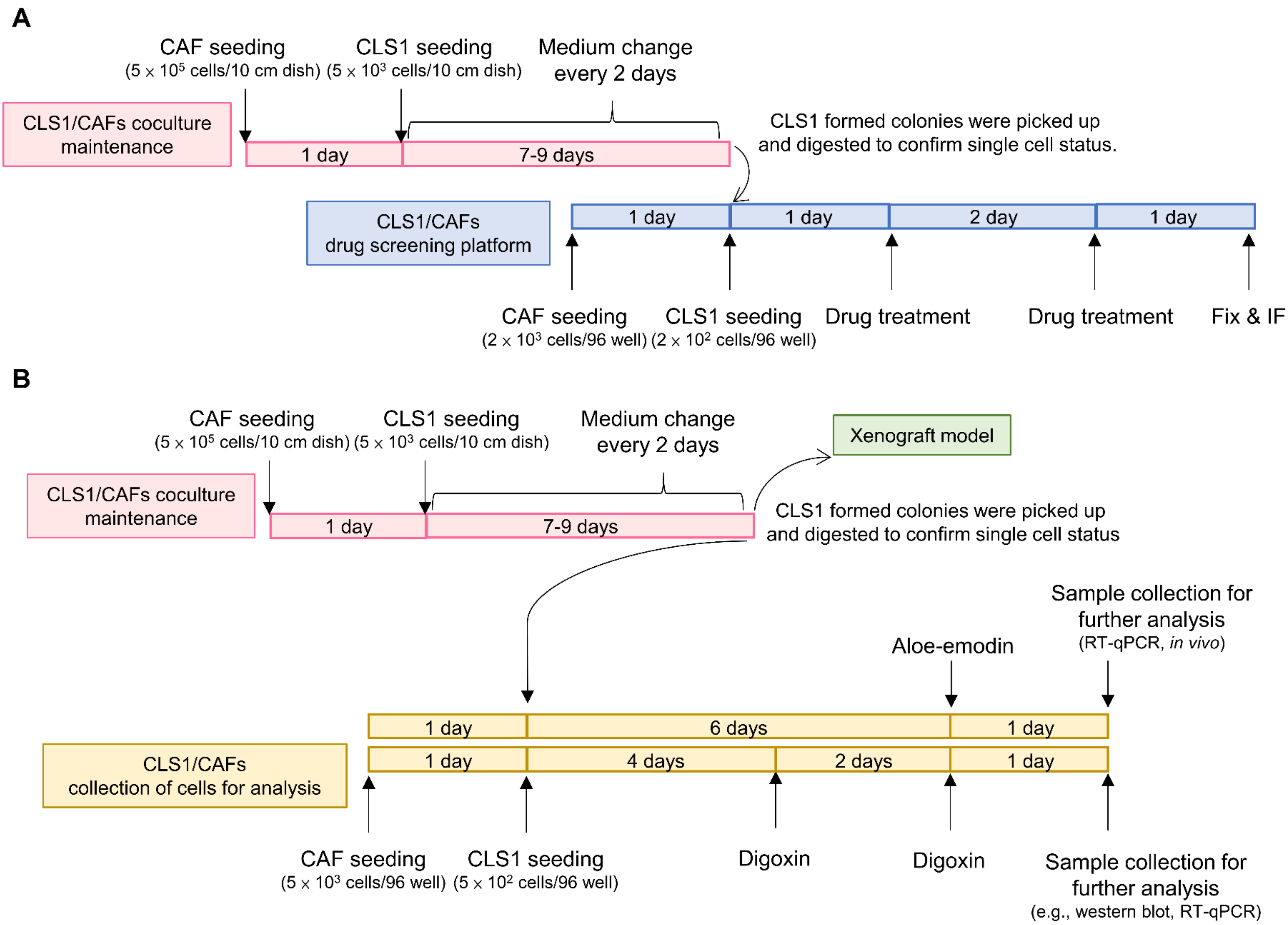


**Figure S2. Timeline of CLS1/CAFs coculture drug screening and drug treatment.**

(A) Timeline of CLS1/CAFs drug screening platform.

(B) Drug treatment timeline of CLS1/CAFs coculture for further analysis (e.g., western blotting, RT-qPCR, *in vivo*…etc.).

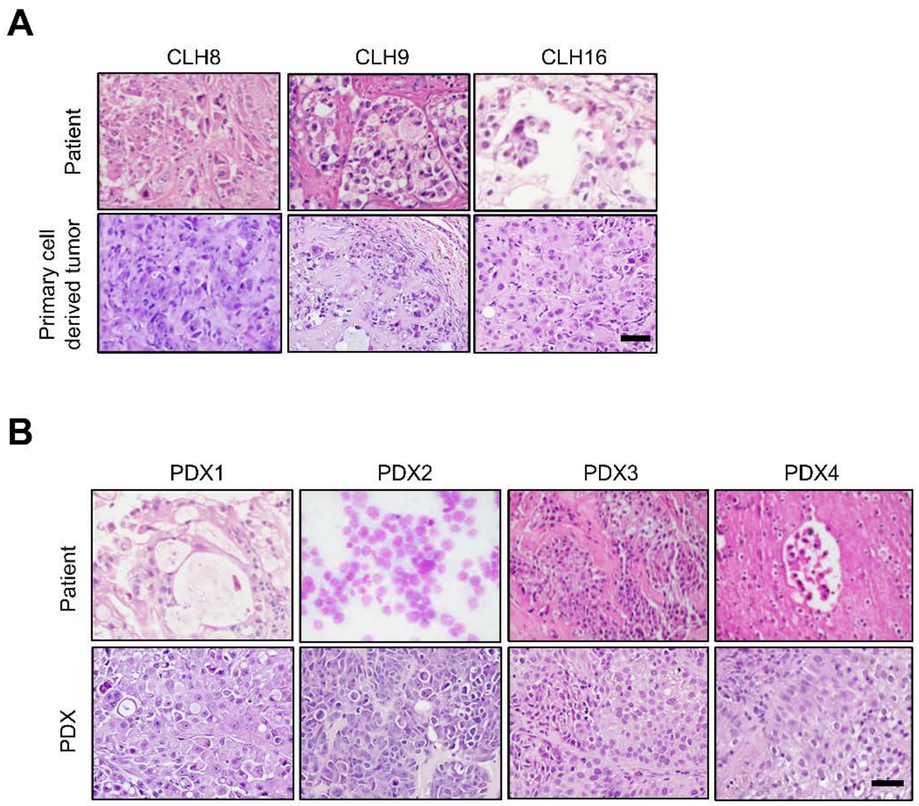


**Figure S3. The histology with H&E stain of tumor sections.**

(A) Primary lung cancer cells-derived tumor in xenograft model and corresponding patient's sample. (Scale: 50 μm)

(B) Patients-derived xenograft (PDX) model and corresponding patient's sample. (Scale: 50 μm)

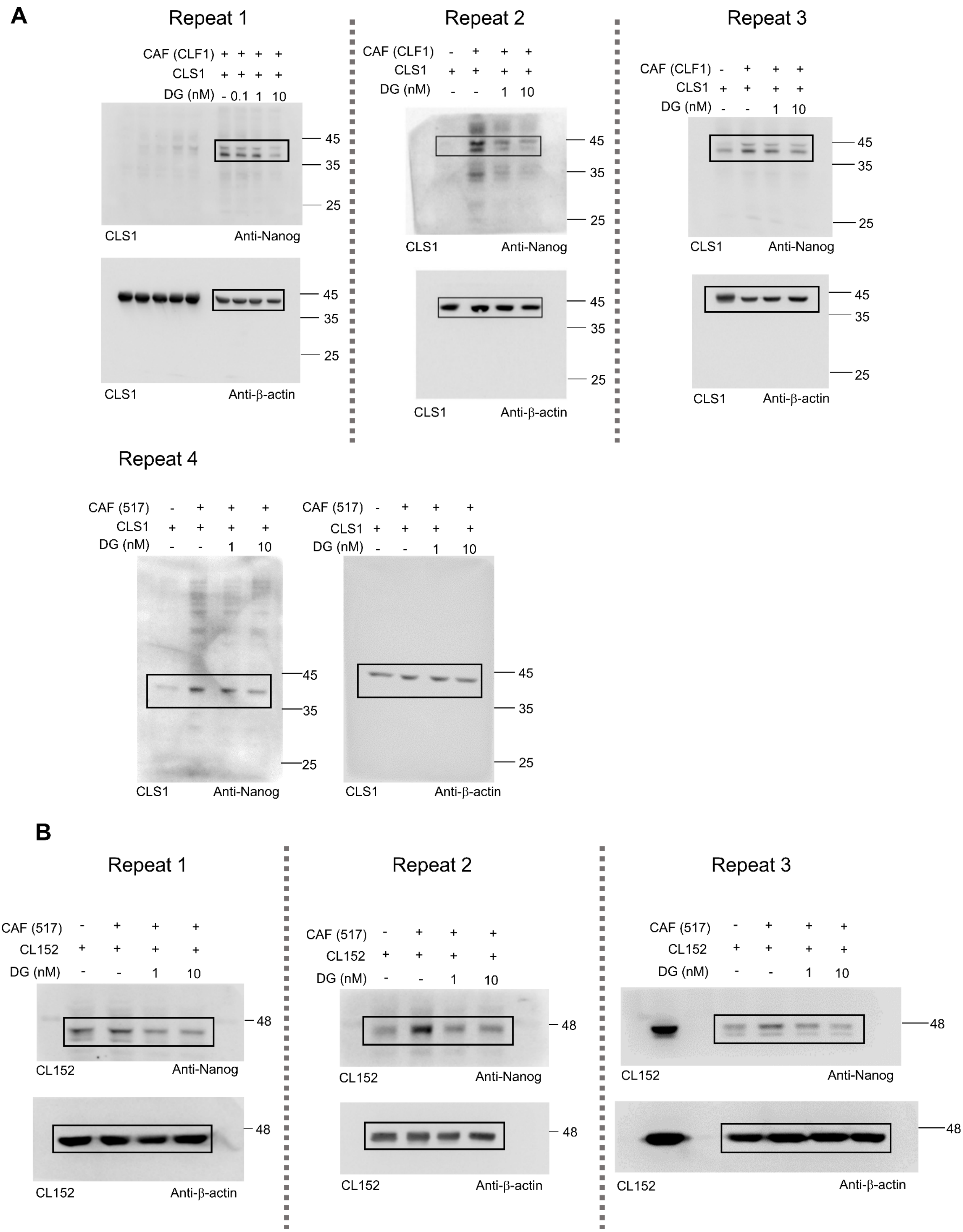
A
Repeat 1
CAF (CLF1) + + + +
CLS1 + + + +
DG (nM) - 0.1 1 10
45
35
25
CLS1
Anti-Nanog
45
35
25
CLS1
Anti-β-actin
Repeat 2
CAF (CLF1) - + + +
CLS1 + + + +
DG (nM) - - 1 10
45
35
25
CLS1
Anti-Nanog
45
35
25
CLS1
Anti-β-actin
Repeat 3
CAF (CLF1) - + + +
CLS1 + + + +
DG (nM) - - 1 10
45
35
25
CLS1
Anti-Nanog
45
35
25
CLS1
Anti-β-actin
Repeat 4
CAF (517) - + + +
CLS1 + + + +
DG (nM) - - 1 10
45
35
25
CLS1
Anti-Nanog
CAF (517) - + + +
CLS1 + + + +
DG (nM) - - 1 10
45
35
25
CLS1
Anti-β-actin
B
Repeat 1
CAF (517) - + + +
CL152 + + + +
DG (nM) - - 1 10
48
CL152
Anti-Nanog
48
CL152
Anti-β-actin
Repeat 2
CAF (517) - + + +
CL152 + + + +
DG (nM) - - 1 10
48
CL152
Anti-Nanog
48
CL152
Anti-β-actin
Repeat 3
CAF (517) - + + +
CL152 + + + +
DG (nM) - - 1 10
48
CL152
Anti-Nanog
48
CL152
Anti-β-actin

**Figure S4. Raw data of western blotting.**

(A) Full unedited gel for Figure 3B and the data for the biological replicates (CLS1).

(B) Full unedited gel for Figure 3B and the data for the biological triplicates (CL152).

DG: digoxin.

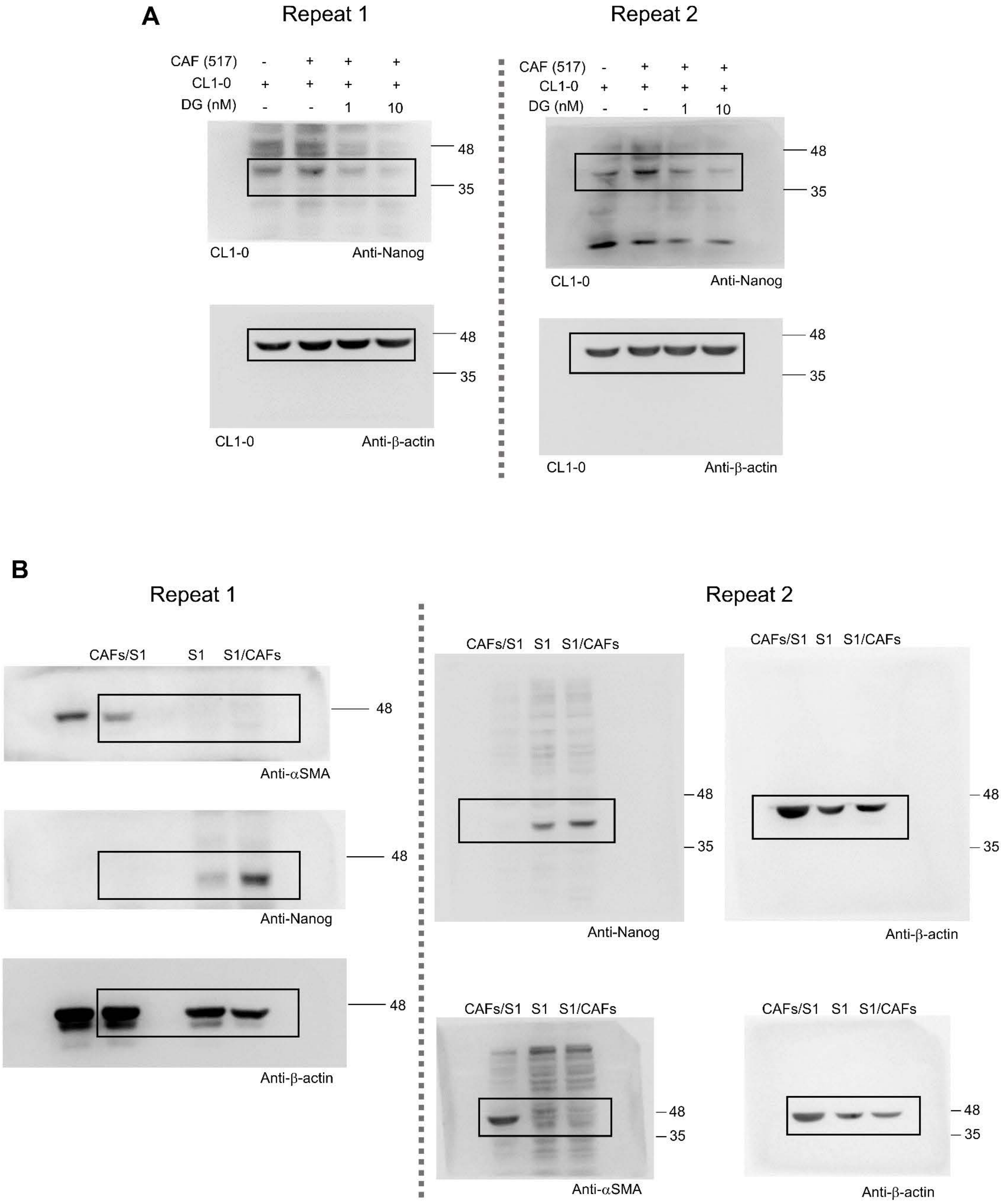
A
Repeat 1
Repeat 2
CAF (517)
CL1-0
DG (nM)
- + + +
+ + + +
- - 1 10
48
35
CL1-0
Anti-Nanog
Anti-β-actin
B
Repeat 1
Repeat 2
CAFs/S1
S1
S1/CAFs
Anti-αSMA
Anti-Nanog
Anti-β-actin
48
35

**Figure S5. Raw data of western blotting.**

(A) Full unedited gel for Figure 3B and the data for the biological duplicates (CL1-0).
(B) Full unedited gel for Figure S1D and the data for the biological duplicates.
DG: digoxin.

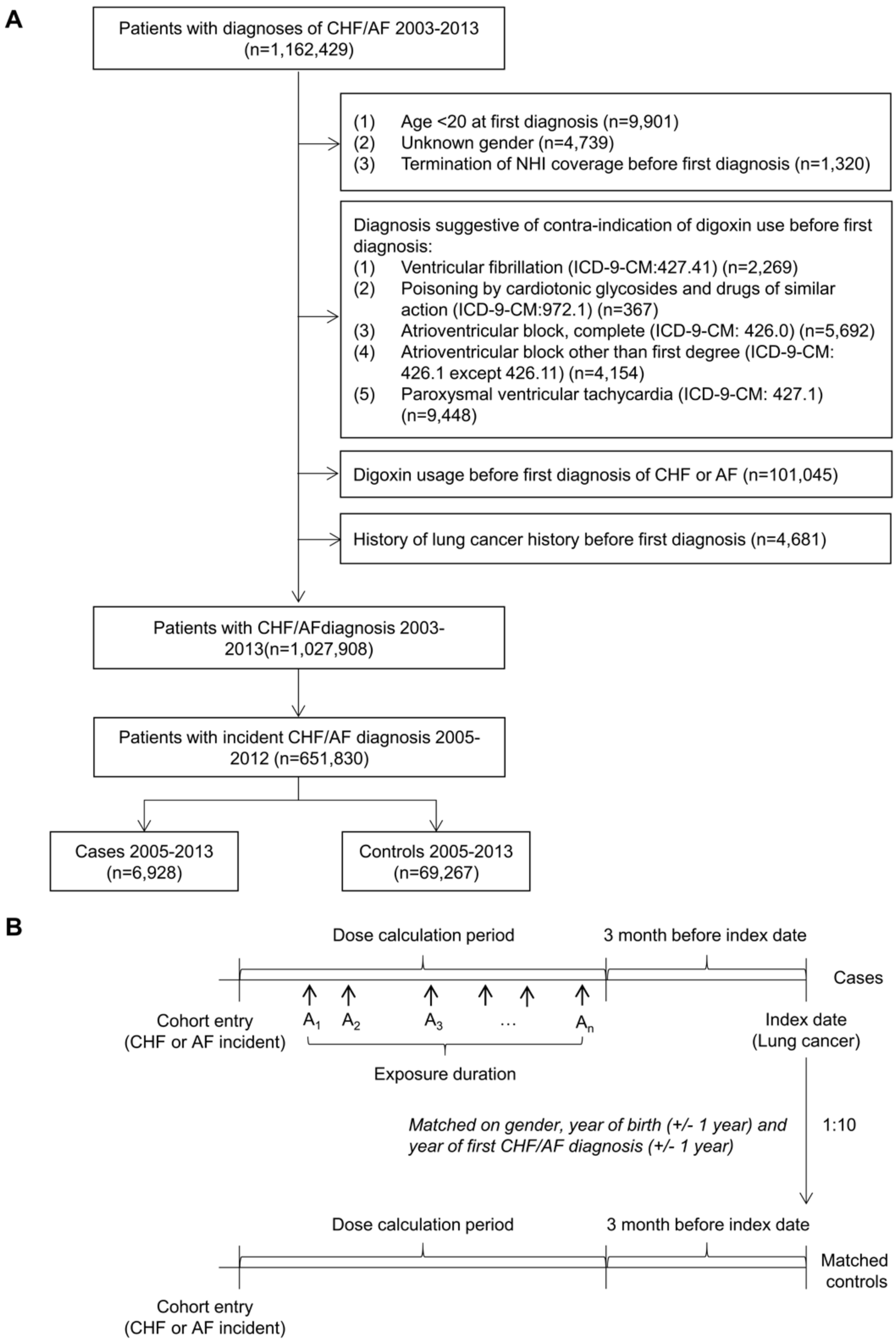
A
Patients with diagnoses of CHF/AF 2003-2013 (n=1,162,429)
(1) Age <20 at first diagnosis (n=9,901)
(2) Unknown gender (n=4,739)
(3) Termination of NHI coverage before first diagnosis (n=1,320)
Diagnosis suggestive of contra-indication of digoxin use before first diagnosis:
(1) Ventricular fibrillation (ICD-9-CM:427.41) (n=2,269)
(2) Poisoning by cardiotonic glycosides and drugs of similar action (ICD-9-CM:972.1) (n=367)
(3) Atrioventricular block, complete (ICD-9-CM: 426.0) (n=5,692)
(4) Atrioventricular block other than first degree (ICD-9-CM: 426.1 except 426.11) (n=4,154)
(5) Paroxysmal ventricular tachycardia (ICD-9-CM: 427.1) (n=9,448)
Digoxin usage before first diagnosis of CHF or AF (n=101,045)
History of lung cancer history before first diagnosis (n=4,681)
Patients with CHF/AFdiagnosis 2003-2013(n=1,027,908)
Patients with incident CHF/AF diagnosis 2005-2012 (n=651,830)
Cases 2005-2013 (n=6,928)
Controls 2005-2013 (n=69,267)
B
Dose calculation period
3 month before index date
Cases
Cohort entry (CHF or AF incident)
$A_1$ $A_2$ $A_3$ … $A_n$
Index date (Lung cancer)
Exposure duration
*Matched on gender, year of birth (+/- 1 year) and year of first CHF/AF diagnosis (+/- 1 year)*
1:10
Dose calculation period
3 month before index date
Matched controls
Cohort entry (CHF or AF incident)

**Figure S6. Design of a nested case-control study.**

(A) Selection flowchart of the nested case-control study.

(B) Digoxin exposure duration and accumulative prescription day definition.

AF: atrial fibrillation (ICD-9-CM: 427.31); CHF: congestive heart failure (ICD-9-CM: 398.91, 402.01, 402.11,402.91, 404.01, 404.03, 404.11,404.13, 404.91, 404.93, 428).

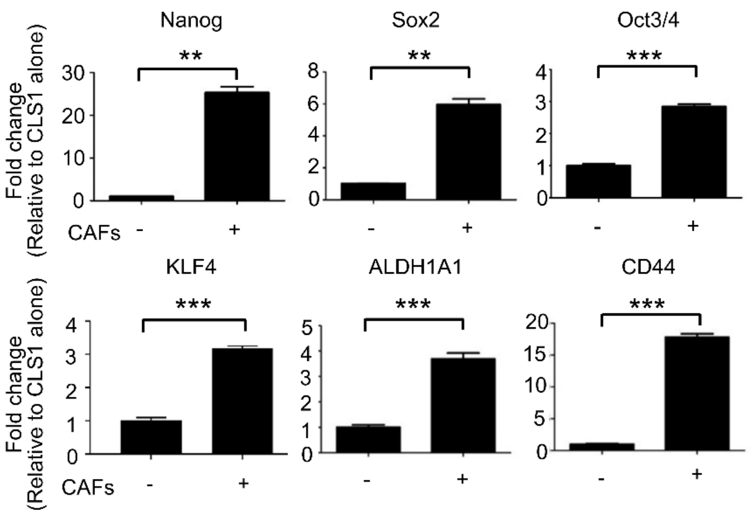


**Figure S7. Validation of CLS1/CAFs coculture system.**

The gene expression of stemness markers in CLS1 alone or cocultured with CAFs (N=3). The data represent the mean±s.e.m. and differences were assessed using Student’s t-test. Results were repeated at least three times.

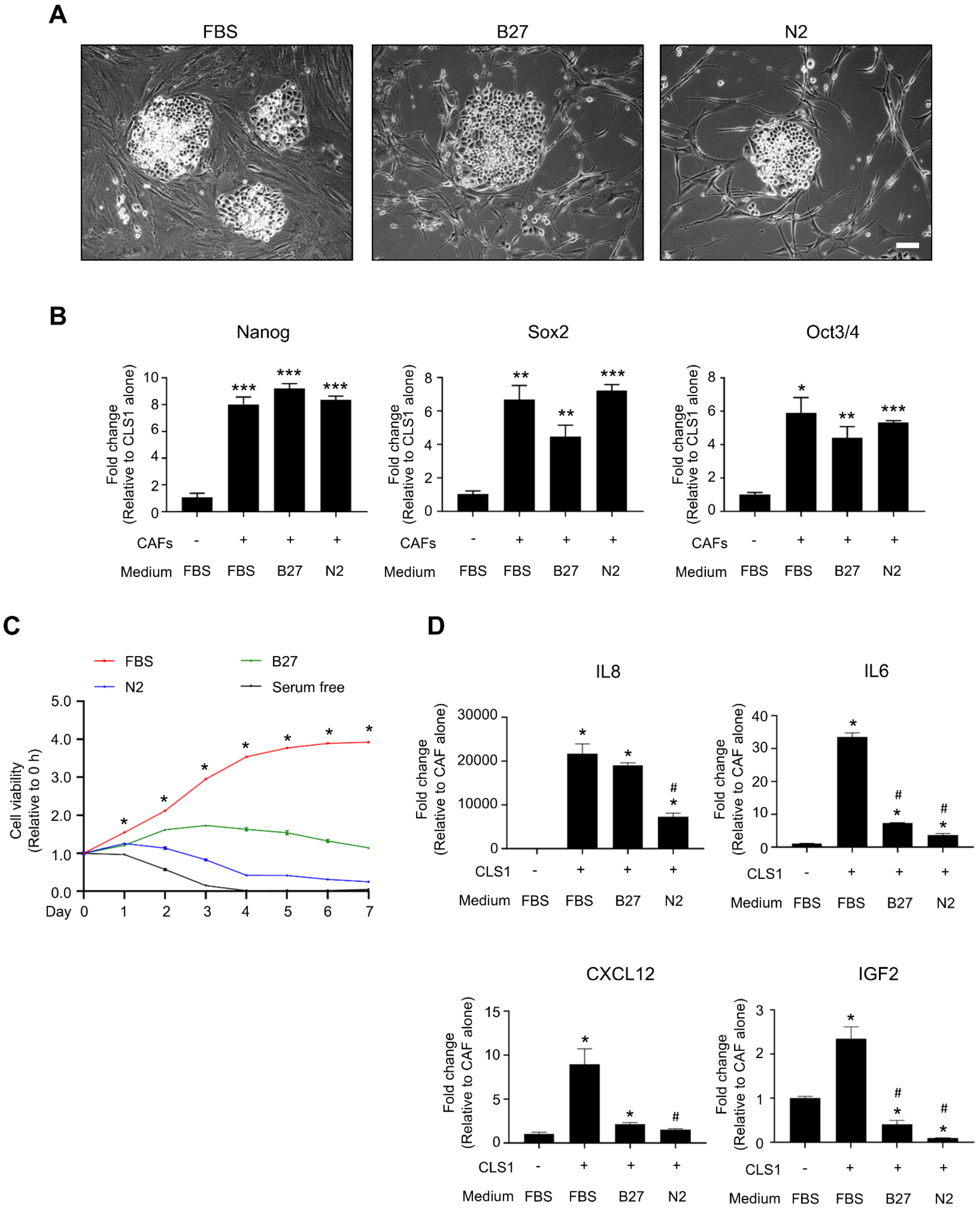
A
FBS
B27
N2
B
Nanog
Sox2
Oct3/4
Fold change (Relative to CLS1 alone)
CAFs - + + +
Medium FBS FBS B27 N2
C
FBS
B27
N2
Serum free
Cell viability (Relative to 0 h)
Day 0 1 2 3 4 5 6 7
D
IL8
IL6
CXCL12
IGF2
Fold change (Relative to CAF alone)
CLS1 - + + +
Medium FBS FBS B27 N2

**Figure S8. Stemness markers of CLS1 cells and cytokines of CAFs under different culture conditions.**

(A) Morphology of CLS1/CAFs coculture cells in FBS (10% FBS), B27 (20 ng/mL EGF and bFGF and 2% B27) and N2 (20 ng/mL EGF and bFGF and 1% N2) culture media (scale: 100 μm).

(B) CLS1/CAFs coculture cells were maintained under different culture conditions After culturing for a total of 9 days, CD90 bead separation was used to separate the CLS1 cells and CAFs. The stemness markers of the CLS1 cells were examined by RT q-PCR (N=3).

(C) Cell viability of CAFs under different culture conditions, including FBS (10% FBS), B27 (20 ng/mL EGF and bFGF and 2% B27) and N2 (20 ng/mL EGF and bFGF and 1% N2) (N=4).

(D) CLS1/CAFs coculture cells were maintained under different culture conditions After culturing for a total of 9 days, CD90 bead separation was used to separate the CLS1 cells and CAFs. The cytokines of CAFs were examined by RT q-PCR (N=3).

The data represent the mean±s.e.m. and the differences were tested by one-way analysis of variance (ANOVA). *Compared to CLS1 alone (B); B27, N2 and serum free (C); CAF alone (D); #compared to CLS1-cocultured CAFs under 10% FBS conditions (D). Results were repeated at least two times.

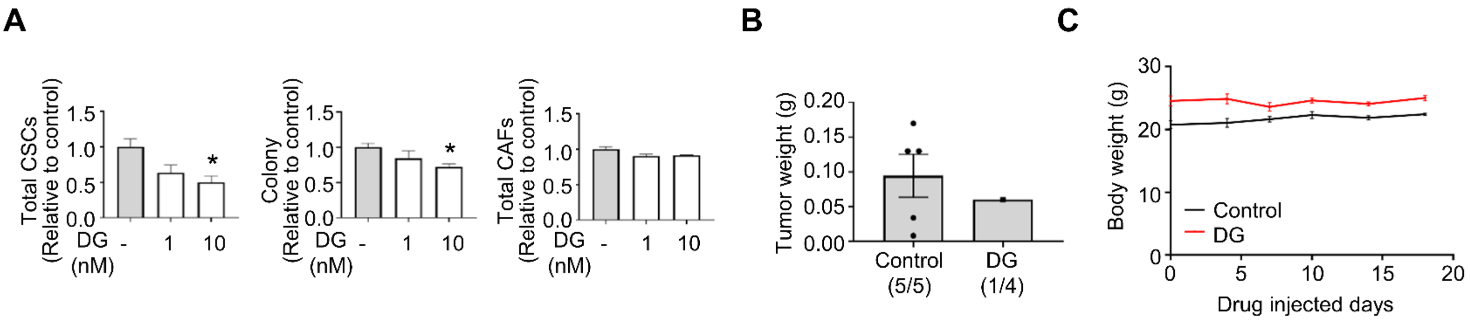


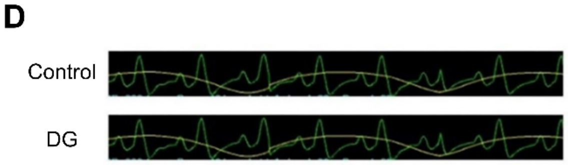


| | | | Control | DG |
|---|---|---|---|---|
| Long axis | | Heart rate(bpm) | 368±34 | 348±18 |
| | Diastolic | IVS(mm) | 1.16±0.15 | 0.84±0.11 |
| | | LVID(mm) | 3.41±0.49 | 3.46±0.26 |
| | | LVPW(mm) | 0.87±0.27 | 1.28±0.34 |
| | Systolic | IVS(mm) | 1.76±0.30 | 1.25±0.19 |
| | | LVID(mm) | 1.74±0.47 | 2.14±0.15 |
| | | LVPW(mm) | 1.56±0.43 | 1.35±0.23 |
| Short axis | | Heart rate(bpm) | 375±23 | 365±22 |
| | Diastolic | IVS(mm) | 1.22±0.26 | 1.16±0.42 |
| | | LVID(mm) | 2.60±1.00 | 3.18±0.86 |
| | | LVPW(mm) | 1.55±0.46 | 1.36±0.57 |
| | Systolic | IVS(mm) | 1.44±0.37 | 1.54±0.47 |
| | | LVID(mm) | 2.30±0.62 | 1.65±0.67 |
| | | LVPW(mm) | 1.56±0.37 | 1.89±0.51 |

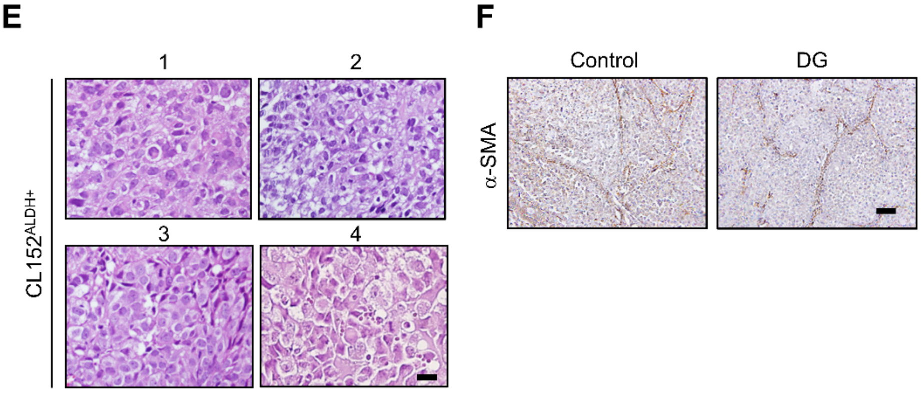


**Figure S9. Digoxin inhibited CSCs subpopulation.**

(A) Total CSCs, colonies and total CAFs were validated in CL152$^{ALDH+}$/CAFs cocultured cells treated with digoxin for 72 h by HCA (N=3).

(B) The tumor weight of CLS1 xenograft model after digoxin treatment (2mg/kg/day) *in vivo* (N=5 for control; N=4 for digoxin, 30 cells/mouse).

(C) Body weight of mice treated with vehicle control and digoxin (2mg/kg/day) (N=4 for control; N=5 for digoxin).

(D) Electrocardiograms of mice treated with vehicle control or digoxin (2mg/kg/day) (N=3).

(E) H&E staining of CL152$^{ALDH+}$ control tumor (scale: 20 μm).

(F) IHC staining of α-SMA expression in tumor sections from the PDX1 model. (Scale: 100 μm). The data represent the mean±s.e.m. (A-C); mean±sd (D); the differences were tested by Student's t-test. Results were repeated at least two times (A, C, F). DG: digoxin; IVS: interventricular septum; LVID: left ventricle inner dimension; LVPW: left ventricular posterior wall.

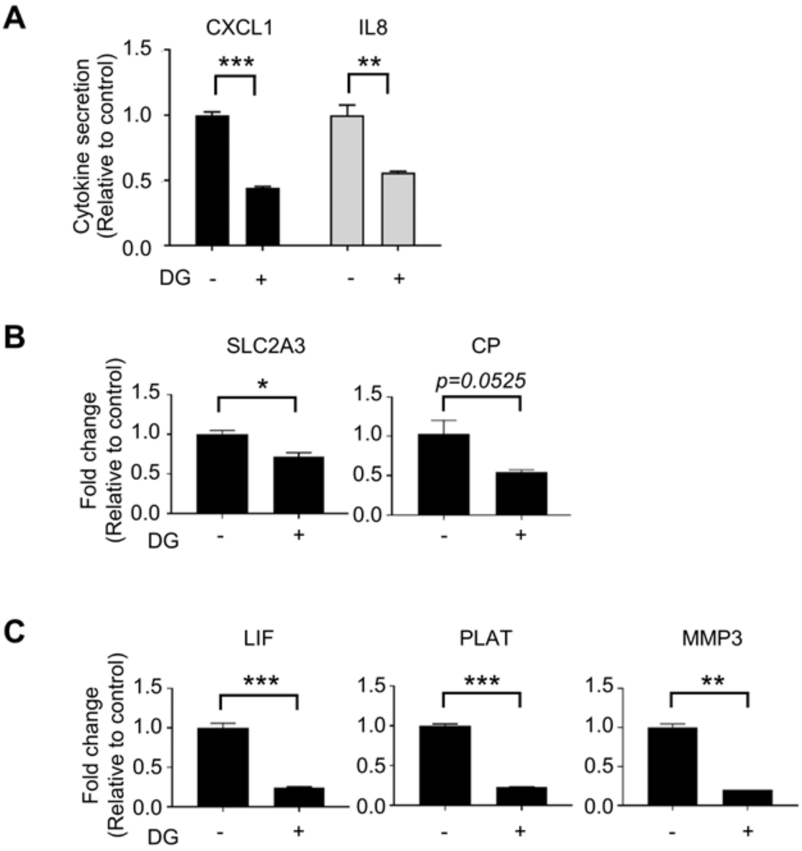


**Figure S10. Digoxin inhibited the expression of stemness-related genes in CLS1 and cytokines in CAFs.**

(A) Cytokine secretion of CLS1/CAFs coculture medium with or without digoxin (10 nM, 72 h) (N=3).

(B) Gene expression of stemness markers in CAFs-cocultured CLS1 with or without digoxin (1 nM, 72 h).

(C) Gene expression of cytokines in CLS1-cocultured CAFs with or without digoxin (1 nM, 72 h) (N=3).

The data represent the mean±s.e.m. and the differences were tested by Student's t-test. Results were repeated at least two times. DG: digoxin.

**Table S1 The status of primary lung cancer cells and PDX model**

| Cells | Gender | Cancer type | EGFR | Other mutation | Cell numbers for tumor initiation experiments *in vivo* | Digoxin treatment time *in vivo* (day) |
|---|---|---|---|---|---|---|
| CLS1 | M | ASC | WT | KRAS Q61H<br>p53 P72R<br>PIK3CA E545K<br>ERBB2 I655V | 100<br>30 | 18<br>36 |
| CL1-0 | M | ADC | WT | p53 R248W | $10^4$ | Unsorted: 24<br>ALDH sorted: 18 |
| CL1-5 | M | ADC | WT | p53 R248W | $10^4$ | 20 |
| CL25 | M | ADC | Ex19Del | p53 C135Y | $10^4$ | 33 |
| CL83 | M | ADC | WT | N/A | $10^4$ | 20 |
| CL100 | M | SCLC | Ex19Del | N/A | $10^4$ | 20 |
| CL141 | M | ADC | WT | PTEN loss | $10^4$ | 33 |
| CL152 | M | SCC | WT | PTEN loss | $10^4$ | Unsorted: 33<br>ALDH: 54 |
| CLH8 | M | ADC | WT | N/A | $10^4$ (Unsorted)<br>$6.5\times10^3$ ($ALDH^+$ sorted) | Unsorted: 35<br>ALDH: 28 |
| CLH9 | M | ADC | Ex19Del | N/A | $10^4$ | 33 |

| | | | | | | |
|---|---|---|---|---|---|---|
| CLH16 | M | ADC | WT | N/A | $10^4$ | Unsorted: 42<br>ALDH sorted: 76 |
| CLY1 | M | ADC | WT | YAP1 R331W | $10^4$ | 14 |
| CLB1 | M | ADC | L858R<br>E709G | N/A | $10^4$ | Unsorted: 33<br>ALDH sorted: 37 |
| PC9 | M | ADC | Ex19Del | p53 R248W | - | - |
| PDX1 | M | ADC | WT | N/A | - | 31 |
| PDX2 | M | ADC | N/A | N/A | - | 23 |
| PDX3 | F | ADC | L858R<br>T790M | N/A | - | 18 |
| PDX4 | F | ADC | N/A | N/A | - | 53 |

M: Male

F: Female

ASC: Adenosquamous carcinoma

ADC: Adenocarcinoma

SCLC: Small cell lung cancer

SCC: Squamous cell carcinoma

N/A: Not applicable

**Table S3 The primer sequence for RT-qPCR**

| Gene | Sequence |
|---|---|
| Nanog-F | CACCAGTCCCAAAGGCAAAC |
| Nanog-R | GCCTTCTGCGTCACACCATT |
| Sox2-F | CACACTGCCCCTCTCACACAT |
| Sox2-R | CCCATTTCCCTGGTTTTTCTT |
| Oct3/4-F | TTCAGCCAAACGACCATCTG |
| Oct3/4-R | GAACCACACTCGGACCACATC |
| CD44-F | CGCAGATCGATTTGAATATAACC |
| CD44-R | CCGATGCTCAGAGCTTTCTC |
| ALDH1A1-F | TCCACATTCCAGTTTGGCCC |
| ALDH1A1-R | TTCGAAGAAGTGTTGAGCG |
| KLF4-F | CCCTTGAATTGTGTATTGATGCA |
| KLF4-R | TCACCAAGCACCATCATTTAGG |
| YAP1-F | CGCTCTTCAACGCCGTCA |
| YAP1-R | AGTACTGGCCTGTCGGGAGT |
| IL6-F | CAGCCCTGAGAAAGGAGACATG |
| IL6-R | TCTGCCAGTGCCTCTTTGC |
| IL8-F | CAGAGACAGCAGAGCACACA |
| IL8-R | TGGCAAAACAGCACCTTCAC |
| IGFII -F | GGCGGCATTTGGGATACA |
| IGFII -R | TCTGTCATGGTGGAAAGATGGA |
| CXCL12-F | TGCCAGAGCCAACGTCAA |
| CXCL12-R | GCTACAATCTGAAGGGCACAGTT |
| CXCL1-F | CGCCCAAACCGAAGTCATAG |
| CXCL1-R | CAGGATTGAGGCAAGCTTTCC |
| IGFBP2-F | CATCCCCAACTGTGACAAGC |
| IGFBP2-R | TGCCCGTTCAGAGACATCTT |
| CP-F | TCCAATACAAGCACAGGGGAG |
| CP-R | CAGCCAGATTTGGTGTCTTCA |
| SCL2A3-F | CACAGGTTTTGTGCCCATGT |
| SCL2A3-R | GGTAAAACCCAGTAGCAGCGG |
| CXCL1-F | CGCCCAAACCGAAGTCATAG |
| CXCL1-R | CAGGATTGAGGCAAGCTTTCC |
| LIF-F | TATTACACAGCCCAGGGGGA |
| LIF-R | ACGACTATGCGGTACAGCTC |
| PLAT-F | GCAACCGGGTGGAATATTGCTG |
| PLAT-R | CGTGGCCCTGGTATCTATTTCA |
| MMP3-F | AGGCTGTATGAAGGAGAGGC |
| MMP3-R | TGGGCCAAAACATTTCCAGG |
| β-actin-F | CTGGAACGGTGAAGGTGACA |
| β-actin-R | CGGCCACATTGTGAACTTTG |
| TBP-F | CACGAACCACGGCACTGATT |
| TBP-R | TTTTCTTGCTGCCAGTCTGGAC |

**Table S4 Baseline characteristics of cases and controls**

| | **Cases<br>N (%)** | **Controls<br>N (%)** |
|---|---|---|
| | N=6,928 | N=69,267 |
| **Male, n (%)** | 4,521 (65.3) | 45,197 (65.3) |
| **Age, median (Q1-Q3)** | 75.0 (68.0-80.0) | 75.0 (68.0-80.0) |
| **Comorbidities, n (%)** | | |
| **Cardiovascular disease** | 5,691 (82.1) | 57,247 (82.6) |
| **Myocardial infarction** | 330 (4.8) | 3,173 (4.6) |
| **Ischemic heart disease** | 2,126 (30.7) | 21,193 (30.6) |
| **Cardiac arrhythmias** | 1,322 (19.1) | 13,320 (19.2) |
| **Valvular heart disease** | 432 (6.2) | 4,736 (6.8) |
| **Hypertension** | 4,419 (63.8) | 45,817 (66.1) |
| **Cerebrovascular disease** | 1,145 (16.5) | 12,991 (18.8) |
| **Chronic kidney disease** | 1,662 (24.0) | 18,318 (26.4) |
| **Chronic liver disease** | 502 (7.2) | 5,058 (7.3) |
| **Diabetes** | 1,641 (23.7) | 17,006 (24.6) |
| **Depression** | 193 (2.8) | 1,984 (2.9) |
| **Dementia** | 211 (3.0) | 2,750 (4.0) |
| **Rheumatologic disease** | 74 (1.1) | 731 (1.1) |
| **Peptic ulcer disease** | 1,250 (18.0) | 12,288 (17.7) |
| **Chronic obstructive pulmonary disease** | 2,196 (31.7) | 16,434 (23.7) |
| **Medication, n (%)** | | |
| **ACE Inhibitors** | 2,334 (33.7) | 24,284 (35.1) |
| **Angiotensin II antagonists** | 2,366 (34.2) | 24,597 (35.5) |
| **Beta blocking agents** | 2,729 (39.4) | 28,060 (40.5) |
| **Calcium channel blockers** | 4,373 (63.1) | 44,620 (64.4) |
| **Hydralazine** | 144 (2.1) | 1,475 (2.1) |
| **Renin inhibitors** | 10 (0.1) | 91 (0.1) |
| **Amiodarone** | 558 (8.1) | 5,498 (7.9) |
| **Isosorbide mononitrate** | 1,387 (20.0) | 12,910 (18.6) |
| **Isosorbide dinitrate** | 845 (12.2) | 8,708 (12.6) |
| **Aspirin** | 3,249 (46.9) | 34,176 (49.3) |
| **Clopidogrel** | 653 (9.4) | 6,589 (9.5) |
| **Warfarin** | 192 (2.8) | 2,466 (3.6) |
| **Fibrates** | 355 (5.1) | 3,826 (5.5) |
| **Statins** | 1,277 (18.4) | 13,143 (19.0) |
| **Bumetanide** | 204 (2.9) | 1,998 (2.9) |
| **Furosemide** | 2,823 (40.7) | 26,184 (37.8) |
| **Thiazide diuretics** | 2,221 (32.1) | 21,960 (31.7) |
| **Other potassium-sparing agents** | 447 (6.5) | 4,664 (6.7) |
| **NSAIDs** | 4,944 (71.4) | 48,778 (70.4) |

**Table S5 Digoxin usage pattern (until 3 months before index date)—Lung cancer (cases before 2013/12/31)**

| | Case | Control |
|---|---|---|
| | n=6928 | n=69267 |
| Digoxin user, n (%) | | |
| until 3 months before index date | 1107 (16.0) | 12208 (17.6) |
| Dosage in user | | |
| < 4 | 226 (3.3) | 2496 (3.6) |
| 4 – 14.9 | 292 (4.2) | 3034 (4.4) |
| 15 – 59.9 | 246 (3.6) | 3129 (4.5) |
| 60 + | 343 (5.0) | 3549 (5.1) |
| medium (Q1-Q3) | 14.75 (4.00-74.50) | 15.25 (4.50-64.00) |
| Average Duration in user, month | | |
| < 0.5 | 282 (4.1) | 2989 (4.3) |
| 0.5 – 1.5 | 246 (3.6) | 2582 (3.7) |
| 1.6 – 5.9 | 241 (3.5) | 3074 (4.4) |
| 6.0 + | 338 (4.9) | 3563 (5.1) |
| medium (Q1-Q3) | 1.87 (0.47-8.70) | 2.00 (0.50-7.56) |
| Cumulative prescription days of digoxin in user | | |
| <30 | 428 (6.2) | 4575 (6.6) |
| 30-59 | 147 (2.1) | 1752 (2.5) |
| 60-89 | 88 (1.3) | 908 (1.3) |
| 90 + | 444 (6.4) | 4973 (7.2) |
| 90-179 | 110 (1.6) | 1372 (2.0) |
| 180-364 | 100 (1.4) | 1353 (2.0) |
| 365+ | 234 (3.4) | 2248 (3.2) |
| medium (Q1-Q3) | 53 (15-274) | 56 (15-235) |

**Table S6 Cancer stem cell frequency of CLS1 after digoxin treatment *in vitro***

| Group | Number of spheres/numbers of well | | | | Cancer stem cell frequency | | Fold reduction | Probability |
|---|---|---|---|---|---|---|---|---|
| | Cells/well | | | | | | | |
| | 500 | 100 | 10 | 1 | Estimate | (95% Confidence interval) | | |
| Control | 48/48 | 41/48 | 47/96 | 16/35 | 1/22.6 | (1/17.1-1/29.9) | 1.5 | p<0.05 |
| Digoxin | 48/48 | 37/48 | 39/96 | 10/29 | 1/34.6 | (1/26.2-1/45.7) | | |

**Table S7 $IC_{50}$ of digoxin in different cells**

| Drug | $IC_{50}$ (nM) | | | | | | |
|---|---|---|---|---|---|---|---|
| | CSCs | | Cancer cells | | Normal cells | Fibroblast | |
| | CLS1/CAFs | $CL152^{ALDH+}$/CAFs | CLS1 | CL152 | BEAS-2B | NFs | CAFs |
| Digoxin | 0.8 ± 0.4 | 2.5 ± 4.1 | 78.6 ± 94.3 | 75.6 ± 21.9 | 138.9 ± 18.3 | 23.7 ± 4.0 | 23.3 ± 9.7 |

**Table S8 Cancer stem cell frequency of CLS1 after digoxin treatment *in vivo***

| Group | Number of tumors/numbers of injection | | Cancer stem cell frequency | | Fold reduction | Probability |
|---|---|---|---|---|---|---|
| | Cells/injection | | | | | |
| | 100 | 30 | Estimate | (95% Confidence interval) | | |
| Control | 7/8 | 5/5 | 1/28.7 | (1/13.2-1/63.4) | 10.5 | $p < 0.001$ |
| Digoxin | 2/9 | 1/4 | 1/300.2 | (1/95.5-1/946.1) | | |

**Table S9 The association between digoxin use and lung cancer**

| | Cases (n=6,928) | Controls (n=69,267) | Crude OR (95 % CI)[a] | Adjusted OR (95% CI) [b,c] |
|---|---|---|---|---|
| **Digoxin use** | | | | |
| No use | 5,443 (78.6%) | 55,831 (80.6%) | 1.00 (reference) | 1.00 (reference) |
| Any use | 1,485 (21.4%) | 13,436 (19.4%) | - | - |
| Use only during the 90 days before lung cancer diagnosis | 378 (5.5%) | 1,228 (1.8%) | - | - |
| Otherwise | 1,107 (16.0%) | 12,208 (17.6%) | 0.92 (0.86-0.99) | 0.89 (0.83-0.96) |
| Cumulative days of digoxin prescriptions[a] | | | | |
| <30 days | 428 (38.7%) | 4,575 (37.5%) | 0.96 (0.86-1.06) | 0.93 (0.84-1.03) |
| 30-89 days | 235 (21.2%) | 2,660 (21.8%) | 0.90 (0.79-1.03) | 0.87 (0.76-1.00) |
| 90+ days | 444 (40.1%) | 4,973 (40.7%) | 0.90 (0.81-1.00) | 0.87 (0.79-0.97) |

a. Cumulative days of digoxin prescriptions was calculated from the date of first CHF/AF diagnosis through the lung cancer diagnosis, excluding digoxin use within 90 days prior to the index date.
b. OR: odds ratio; CI: confidence interval.
c. Based on conditional logistic regression, adjusted for income and comorbidities.
d. The confounding factors (e.g., smoking and family history) were not taken into consideration due to limitations in the database.

**Table S10 Cancer stem cell frequency of CLS1 after digoxin, cisplatin treatment *in vitro***

| Group | Number of spheres/numbers of well<br>Cells/well | | | Cancer stem cell frequency | | Probability (Compare to control) | Probability (Compare to cisplatin) |
|---|---|---|---|---|---|---|---|
| | 1000 | 500 | 100 | Estimate | (95% Confidence interval) | | |
| Control | 104/120 | 38/120 | 3/120 | 1/822 | (1/697-1/969) | | |
| Digoxin | 98/120 | 40/120 | 2/120 | 1/878 | (1/743-1/1038) | | |
| Cisplatin | 106/120 | 77/120 | 12/120 | 1/512 | (1/440-1/596) | $p < 0.001$ | |
| Combine | 93/144 | 41/144 | 0/144 | 1/1204 | (1/1018-1/1424) | $p = 0.002$ | $p < 0.001$ |

**Table S2 Drug list**

| Drug Name | Library | Dose |
|---|---|---|
| DL-alpha-Methyl-p-tyrosine | Lopac | 1 microM |
| 6-Methoxy-1,2,3,4-tetrahydro-9H-pyrido[3,4b] indole | Lopac | 1 microM |
| Acetamide | Lopac | 1 microM |
| Amantadine hydrochloride | Lopac | 1 microM |
| GABA | Lopac | 1 microM |
| Gabaculine hydrochloride | Lopac | 1 microM |
| O-(Carboxymethyl)hydroxylamine hemihydrochloride | Lopac | 1 microM |
| (±)-2-Amino-7-phosphonoheptanoic acid | Lopac | 1 microM |
| N-Acetylprocainamide hydrochloride | Lopac | 1 microM |
| Actinonin | Lopac | 1 microM |
| N-Phenylanthranilic acid | Lopac | 1 microM |
| S-(4-Nitrobenzyl)-6-thioguanosine | Lopac | 1 microM |
| Org 24598 lithium salt | Lopac | 1 microM |
| Aminophylline ethylenediamine | Lopac | 1 microM |
| 3'-Azido-3'-deoxythymidine | Lopac | 1 microM |
| YM 976 | Lopac | 1 microM |
| 5-(N,N-Dimethyl)amiloride hydrochloride | Lopac | 1 microM |
| (±)-2-Amino-5-phosphonopentanoic acid | Lopac | 1 microM |
| Sodium Taurocholate hydrate | Lopac | 1 microM |
| Methotrexate hydrate | Lopac | 1 microM |
| S(-)-p-Bromotetramisole oxalate | Lopac | 1 microM |
| TMB-8 hydrochloride | Lopac | 1 microM |
| L-azetidine-2-carboxylic acid | Lopac | 1 microM |
| S-(p-Azidophenacyl)glutathione | Lopac | 1 microM |
| Acetyl-beta-methylcholine chloride | Lopac | 1 microM |
| AA-861 | Lopac | 1 microM |
| Azathioprine | Lopac | 1 microM |
| L-732,138 | Lopac | 1 microM |
| Amifostine | Lopac | 1 microM |

| | | |
|---|---|---|
| Atropine methyl bromide | Lopac | 1 microM |
| 5-Aminovaleric acid hydrochloride | Lopac | 1 microM |
| 4-Aminopyridine | Lopac | 1 microM |
| p-Aminoclonidine hydrochloride | Lopac | 1 microM |
| Aminopterin | Lopac | 1 microM |
| 5-azacytidine | Lopac | 1 microM |
| 9-Amino-1,2,3,4-tetrahydroacridine hydrochloride | Lopac | 1 microM |
| Acyclovir | Lopac | 1 microM |
| Acetylsalicylic acid | Lopac | 1 microM |
| Acetazolamide | Lopac | 1 microM |
| Amperozide hydrochloride | Lopac | 1 microM |
| (±)-Nipecotic acid | Lopac | 1 microM |
| Atropine sulfate | Lopac | 1 microM |
| 3-aminobenzamide | Lopac | 1 microM |
| N-Acetyl-5-hydroxytryptamine | Lopac | 1 microM |
| 5-(N-Ethyl-N-isopropyl)amiloride | Lopac | 1 microM |
| 10058-F4 | Lopac | 1 microM |
| Amiprilose hydrochloride | Lopac | 1 microM |
| 5-(N-Methyl-N-isobutyl)amiloride | Lopac | 1 microM |
| Arecoline hydrobromide | Lopac | 1 microM |
| Aminoguanidine hemisulfate | Lopac | 1 microM |
| Azelaic acid | Lopac | 1 microM |
| Atropine methyl nitrate | Lopac | 1 microM |
| (±)-Norepinephrine (+)bitartrate | Lopac | 1 microM |
| Aurintricarboxylic acid | Lopac | 1 microM |
| 3-Aminopropionitrile fumarate | Lopac | 1 microM |
| 1-Aminobenzotriazole | Lopac | 1 microM |
| Sandoz 58-035 | Lopac | 1 microM |
| Acetylthiocholine chloride | Lopac | 1 microM |
| A-315456 | Lopac | 1 microM |
| Agmatine sulfate | Lopac | 1 microM |

| | | |
|---|---|---|
| Tryptamine hydrochloride | Lopac | 1 microM |
| Arcaine sulfate | Lopac | 1 microM |
| 4-Amino-1,8-naphthalimide | Lopac | 1 microM |
| (±)-2-Amino-4-phosphonobutyric acid | Lopac | 1 microM |
| Apigenin | Lopac | 1 microM |
| 3-Amino-1-propanesulfonic acid sodium | Lopac | 1 microM |
| (±)-2-Amino-3-phosphonopropionic acid | Lopac | 1 microM |
| 4-Androsten-4-ol-3,17-dione | Lopac | 1 microM |
| GR 46611 | Lopac | 1 microM |
| 4-Aminobenzamidine dihydrochloride | Lopac | 1 microM |
| 5-Fluoroindole-2-carboxylic acid | Lopac | 1 microM |
| 1-Aminocyclopropanecarboxylic acid hydrochloride | Lopac | 1 microM |
| Reserpine | Lopac | 1 microM |
| N-arachidonylglycine | Lopac | 1 microM |
| (+)-Butaclamol hydrochloride | Lopac | 1 microM |
| Apomorphine hydrochloride hemihydrate | Lopac | 1 microM |
| L-Arginine | Lopac | 1 microM |
| 2-(2-Aminoethyl)isothiourea dihydrobromide | Lopac | 1 microM |
| 2-Hydroxysaclofen | Lopac | 1 microM |
| 3-Aminopropylphosphonic acid | Lopac | 1 microM |
| N-Acetyl-L-Cysteine | Lopac | 1 microM |
| 6-Aminohexanoic acid | Lopac | 1 microM |
| Altretamine | Lopac | 1 microM |
| Adenosine 3',5'-cyclic monophosphate | Lopac | 1 microM |
| (±)-AMT hydrochloride | Lopac | 1 microM |
| 5'-Amino-5'-deoxyadenosine p-toluenesulfonate salt | Lopac | 1 microM |
| 1-Allyl-3,7-dimethyl-8-p-sulfophenylxanthine | Lopac | 1 microM |
| Acetohexamide | Lopac | 1 microM |
| cis-Azetidine-2,4-dicarboxylic acid | Lopac | 1 microM |
| 2,3-Butanedione monoxime | Lopac | 1 microM |
| L-2-aminoadipic acid | Lopac | 1 microM |

| | | |
|---|---|---|
| ATPO | Lopac | 1 microM |
| 2-(Methylthio)adenosine 5'-diphosphate trisodium salt hydrate | Lopac | 1 microM |
| L(-)-Norepinephrine bitartrate | Lopac | 1 microM |
| Paroxetine hydrochloride hemihydrate (MW = 374.83) | Lopac | 1 microM |
| PNU-37883A | Lopac | 1 microM |
| trans-(±)-ACPD | Lopac | 1 microM |
| SKF-89145 hydrobromide | Lopac | 1 microM |
| trans-Azetidine-2,4-dicarboxylic acid | Lopac | 1 microM |
| SB 222200 | Lopac | 1 microM |
| N-Acetyltryptamine | Lopac | 1 microM |
| Allopurinol | Lopac | 1 microM |
| Aminoguanidine hydrochloride | Lopac | 1 microM |
| 5-(N,N-hexamethylene)amiloride | Lopac | 1 microM |
| Antozoline hydrochloride | Lopac | 1 microM |
| Tracazolate | Lopac | 1 microM |
| Diacylglycerol kinase inhibitor I | Lopac | 1 microM |
| cis-4-Aminocrotonic acid | Lopac | 1 microM |
| CBIQ | Lopac | 1 microM |
| 1-benzoyl-5-methoxy-2-methylindole-3-acetic acid | Lopac | 1 microM |
| Amiloride hydrochloride | Lopac | 1 microM |
| Amitriptyline hydrochloride | Lopac | 1 microM |
| BW 284c51 | Lopac | 1 microM |
| Fulvestrant | Lopac | 1 microM |
| Aniracetam | Lopac | 1 microM |
| Amoxapine | Lopac | 1 microM |
| 1-Amino-1-cyclohexanecarboxylic acid hydrochloride | Lopac | 1 microM |
| N6-2-(4-Aminophenyl)ethyladenosine | Lopac | 1 microM |
| AIDA | Lopac | 1 microM |
| p-Benzoquinone | Lopac | 1 microM |
| (±)-Atenolol | Lopac | 1 microM |
| Amiodarone hydrochloride | Lopac | 1 microM |

| | | |
|---|---|---|
| Adenosine | Lopac | 1 microM |
| (±)-p-Aminoglutethimide | Lopac | 1 microM |
| HEMADO | Lopac | 1 microM |
| Aminobenztropine | Lopac | 1 microM |
| Alaproclate hydrochloride | Lopac | 1 microM |
| Opipramol dihydrochloride | Lopac | 1 microM |
| A-77636 hydrochloride | Lopac | 1 microM |
| 8-Bromo-cGMP sodium | Lopac | 1 microM |
| Chlormethiazole hydrochloride | Lopac | 1 microM |
| 4-(2-Aminoethyl)benzenesulfonyl fluoride hydrochloride | Lopac | 1 microM |
| L-Aspartic acid | Lopac | 1 microM |
| (±)-HA-966 | Lopac | 1 microM |
| 8-(p-Sulfophenyl)theophylline | Lopac | 1 microM |
| Arecaidine propargyl ester hydrobromide | Lopac | 1 microM |
| Psora-4 | Lopac | 1 microM |
| gamma-Acetylinic GABA | Lopac | 1 microM |
| ATPA | Lopac | 1 microM |
| TBBz | Lopac | 1 microM |
| L-allylglycine | Lopac | 1 microM |
| Ancitabine hydrochloride | Lopac | 1 microM |
| Astaxanthin | Lopac | 1 microM |
| Lercanidipine hydrochloride hemihydrate | Lopac | 1 microM |
| 1,3-Dipropyl-8-p-sulfophenylxanthine | Lopac | 1 microM |
| Indirubin-3'-oxime | Lopac | 1 microM |
| SB 200646 hydrochloride | Lopac | 1 microM |
| AB-MECA | Lopac | 1 microM |
| ARL 67156 trisodium salt | Lopac | 1 microM |
| Bromoenol lactone | Lopac | 1 microM |
| ABT-418 hydrochloride | Lopac | 1 microM |
| Alprenolol hydrochloride | Lopac | 1 microM |
| N-(4-Amino-2-chlorophenyl)phthalimide | Lopac | 1 microM |

| | | |
|---|---|---|
| Amsacrine hydrochloride | Lopac | 1 microM |
| 2-Methylthioadenosine triphosphate tetrasodium | Lopac | 1 microM |
| S(-)-Atenolol | Lopac | 1 microM |
| AS-252424 | Lopac | 1 microM |
| Alloxazine | Lopac | 1 microM |
| Beclomethasone | Lopac | 1 microM |
| Benzamide | Lopac | 1 microM |
| 3-Bromo-7-nitroindazole | Lopac | 1 microM |
| Bumetanide | Lopac | 1 microM |
| (±)-Baclofen | Lopac | 1 microM |
| Brefeldin A from Penicillium brefeldianum | Lopac | 1 microM |
| BP 897 | Lopac | 1 microM |
| Bupropion hydrochloride | Lopac | 1 microM |
| BU224 hydrochloride | Lopac | 1 microM |
| Ciprofibrate | Lopac | 1 microM |
| CGP-7930 | Lopac | 1 microM |
| Chlorprothixene hydrochloride | Lopac | 1 microM |
| (+)-Bromocriptine methanesulfonate | Lopac | 1 microM |
| Betaine hydrochloride | Lopac | 1 microM |
| SB 202190 | Lopac | 1 microM |
| Budesonide | Lopac | 1 microM |
| (E)-5-(2-Bromovinyl)-2'-deoxyuridine | Lopac | 1 microM |
| (-)-Bicuculline methbromide, 1(S), 9(R) | Lopac | 1 microM |
| B-HT 933 dihydrochloride | Lopac | 1 microM |
| 6-Chloromelatonin | Lopac | 1 microM |
| CGP-13501 | Lopac | 1 microM |
| Choline bromide | Lopac | 1 microM |
| O6-benzylguanine | Lopac | 1 microM |
| Betaine aldehyde chloride | Lopac | 1 microM |
| Bay 11-7085 | Lopac | 1 microM |
| 8-Bromo-cAMP sodium | Lopac | 1 microM |

| | | |
|---|---|---|
| BRL 15572 | Lopac | 1 microM |
| (±)-Bay K 8644 | Lopac | 1 microM |
| (±)-Butaclamol hydrochloride | Lopac | 1 microM |
| Carmustine | Lopac | 1 microM |
| CP55940 | Lopac | 1 microM |
| BTO-1 | Lopac | 1 microM |
| N-Bromoacetamide | Lopac | 1 microM |
| Benazoline oxalate | Lopac | 1 microM |
| Betaxolol hydrochloride | Lopac | 1 microM |
| Benztropine mesylate | Lopac | 1 microM |
| Chloroethylclonidine dihydrochloride | Lopac | 1 microM |
| Bromoacetylcholine bromide | Lopac | 1 microM |
| BRL 37344 sodium | Lopac | 1 microM |
| PK 11195 | Lopac | 1 microM |
| L-Cycloserine | Lopac | 1 microM |
| CB 1954 | Lopac | 1 microM |
| (±)-Brompheniramine maleate | Lopac | 1 microM |
| BWB70C | Lopac | 1 microM |
| Benzamidine hydrochloride | Lopac | 1 microM |
| Ro 20-1724 | Lopac | 1 microM |
| 6-Fluoronorepinephrine hydrochloride | Lopac | 1 microM |
| BMY 7378 dihydrochloride | Lopac | 1 microM |
| BRL 54443 maleate | Lopac | 1 microM |
| Caffeic Acid | Lopac | 1 microM |
| ML-9 | Lopac | 1 microM |
| S-(+)-PD 123177 trifluoroacetate salt hydrate | Lopac | 1 microM |
| Benzamil hydrochloride | Lopac | 1 microM |
| 5-Bromo-2'-deoxyuridine | Lopac | 1 microM |
| Betamethasone | Lopac | 1 microM |
| Bestatin hydrochloride | Lopac | 1 microM |
| Bromoacetyl alprenolol menthane | Lopac | 1 microM |

| | | |
|---|---|---|
| R(+)-6-Bromo-APB hydrobromide | Lopac | 1 microM |
| Biperiden hydrochloride | Lopac | 1 microM |
| Cilostazol | Lopac | 1 microM |
| (+)-Catechin Hydrate | Lopac | 1 microM |
| Corticosterone | Lopac | 1 microM |
| L-Buthionine-sulfoximine | Lopac | 1 microM |
| Bepridil hydrochloride | Lopac | 1 microM |
| Buspirone hydrochloride | Lopac | 1 microM |
| Bretylium tosylate | Lopac | 1 microM |
| Benoxathian hydrochloride | Lopac | 1 microM |
| BTCP hydrochloride | Lopac | 1 microM |
| Chlorambucil | Lopac | 1 microM |
| Caffeine | Lopac | 1 microM |
| Chlorpropamide | Lopac | 1 microM |
| Carboplatin | Lopac | 1 microM |
| DL-Buthionine-[S,R]-sulfoximine | Lopac | 1 microM |
| (+)-Brompheniramine maleate | Lopac | 1 microM |
| Benserazide hydrochloride | Lopac | 1 microM |
| BRL 50481 | Lopac | 1 microM |
| Phenoxybenzamine hydrochloride | Lopac | 1 microM |
| DAPH | Lopac | 1 microM |
| Supercinnamaldehyde | Lopac | 1 microM |
| Cyclophosphamide monohydrate | Lopac | 1 microM |
| 1-(4-Chlorobenzyl)-5-methoxy-2-methylindole-3-acetic acid | Lopac | 1 microM |
| Cortisone | Lopac | 1 microM |
| Chelerythrine chloride | Lopac | 1 microM |
| Cyclosporin A | Lopac | 1 microM |
| Carbachol | Lopac | 1 microM |
| Cephalexin hydrate | Lopac | 1 microM |
| Roscovitine | Lopac | 1 microM |
| Cyproheptadine hydrochloride | Lopac | 1 microM |

| | | |
|---|---|---|
| VGX-1027 | Lopac | 1 microM |
| Cantharidin | Lopac | 1 microM |
| Chlorpromazine hydrochloride | Lopac | 1 microM |
| Centrophenoxine hydrochloride | Lopac | 1 microM |
| 1-(2-Chlorophenyl)-1-(4-chlorophenyl)-2,2-dichloroethane | Lopac | 1 microM |
| D-Cycloserine | Lopac | 1 microM |
| Chlorzoxazone | Lopac | 1 microM |
| Chlorothiazide | Lopac | 1 microM |
| SB 204741 | Lopac | 1 microM |
| GR 79236X | Lopac | 1 microM |
| Cefaclor | Lopac | 1 microM |
| Citalopram hydrobromide | Lopac | 1 microM |
| Cefsulodin sodium salt hydrate | Lopac | 1 microM |
| Clemastine fumarate | Lopac | 1 microM |
| (±)-Chlorpheniramine maleate | Lopac | 1 microM |
| 8-(4-Chlorophenylthio)-cAMP sodium | Lopac | 1 microM |
| L-Cysteinesulfinic Acid | Lopac | 1 microM |
| (+)-Chlorpheniramine maleate | Lopac | 1 microM |
| Ceftriaxone sodium | Lopac | 1 microM |
| Cefmetazole sodium | Lopac | 1 microM |
| DL-Cycloserine | Lopac | 1 microM |
| Clonidine hydrochloride | Lopac | 1 microM |
| Caffeic acid phenethyl ester | Lopac | 1 microM |
| beta-Chloro-L-alanine hydrochloride | Lopac | 1 microM |
| Cortisone 21-acetate | Lopac | 1 microM |
| Calmidazolium chloride | Lopac | 1 microM |
| 9-cyclopentyladenine | Lopac | 1 microM |
| Cefazolin sodium | Lopac | 1 microM |
| 4-Chloromercuribenzoic acid | Lopac | 1 microM |
| Clozapine | Lopac | 1 microM |
| McN-A-343 | Lopac | 1 microM |

| | | |
|---|---|---|
| Cefotaxime sodium | Lopac | 1 microM |
| Cephapirin sodium | Lopac | 1 microM |
| Pyrocatechol | Lopac | 1 microM |
| Cephalosporin C zinc salt | Lopac | 1 microM |
| GR 113808 | Lopac | 1 microM |
| Cephalothin sodium | Lopac | 1 microM |
| Clemizole hydrochloride | Lopac | 1 microM |
| (-)-Cotinine | Lopac | 1 microM |
| (±)-p-Chlorophenylalanine | Lopac | 1 microM |
| N-(2-[4-(4-Chlorophenyl)piperazin-1-yl]ethyl)-3-methoxybenzamide | Lopac | 1 microM |
| Cilostamide | Lopac | 1 microM |
| Cephradine | Lopac | 1 microM |
| Z-L-Phe chloromethyl ketone | Lopac | 1 microM |
| CGP-74514A hydrochloride | Lopac | 1 microM |
| Carbamazepine | Lopac | 1 microM |
| Cimetidine | Lopac | 1 microM |
| 2-Chloroadenosine | Lopac | 1 microM |
| CL 316,243 | Lopac | 1 microM |
| Chloroquine diphosphate | Lopac | 1 microM |
| Cystamine dihydrochloride | Lopac | 1 microM |
| Chelidamic acid | Lopac | 1 microM |
| DSP-4 hydrochloride | Lopac | 1 microM |
| CPCCOEt | Lopac | 1 microM |
| Cyproterone acetate | Lopac | 1 microM |
| Captopril | Lopac | 1 microM |
| Cyclobenzaprine hydrochloride | Lopac | 1 microM |
| Bethanechol chloride | Lopac | 1 microM |
| 7-Chloro-4-hydroxy-2-phenyl-1,8-naphthyridine | Lopac | 1 microM |
| Clofibrate | Lopac | 1 microM |
| Clomipramine hydrochloride | Lopac | 1 microM |
| N6-Cyclopentyladenosine | Lopac | 1 microM |

| | | |
|---|---|---|
| Cinoxacin | Lopac | 1 microM |
| Colchicine | Lopac | 1 microM |
| DL-p-Chlorophenylalanine methyl ester hydrochloride | Lopac | 1 microM |
| CNS-1102 | Lopac | 1 microM |
| Carbetapentane citrate | Lopac | 1 microM |
| Cinnarizine | Lopac | 1 microM |
| Clotrimazole | Lopac | 1 microM |
| Cytosine-1-beta-D-arabinofuranoside hydrochloride | Lopac | 1 microM |
| Calcimycin | Lopac | 1 microM |
| Cantharidic Acid | Lopac | 1 microM |
| Carisoprodol | Lopac | 1 microM |
| L-Canavanine sulfate | Lopac | 1 microM |
| Cyclothiazide | Lopac | 1 microM |
| (±)-CPP | Lopac | 1 microM |
| CGS-21680 hydrochloride | Lopac | 1 microM |
| CGS-15943 | Lopac | 1 microM |
| Chloro-IB-MECA | Lopac | 1 microM |
| Debrisoquin sulfate | Lopac | 1 microM |
| Diltiazem hydrochloride | Lopac | 1 microM |
| (S)-3,5-Dihydroxyphenylglycine | Lopac | 1 microM |
| Phenytoin sodium | Lopac | 1 microM |
| Daphnetin | Lopac | 1 microM |
| N6-Cyclohexyladenosine | Lopac | 1 microM |
| CGS-12066A maleate | Lopac | 1 microM |
| Y-27632 dihydrochloride | Lopac | 1 microM |
| 2-Chloro-2-deoxy-D-glucose | Lopac | 1 microM |
| WB-4101 hydrochloride | Lopac | 1 microM |
| 2',3'-didehydro-3'-deoxythymidine | Lopac | 1 microM |
| Dextromethorphan hydrobromide monohydrate | Lopac | 1 microM |
| Dequalinium chloride hydrate | Lopac | 1 microM |
| Doxepin hydrochloride | Lopac | 1 microM |

| | | |
|---|---|---|
| DM 235 | Lopac | 1 microM |
| (S)-(+)-Camptothecin | Lopac | 1 microM |
| 2-Cyclooctyl-2-hydroxyethylamine hydrochloride | Lopac | 1 microM |
| 1-(m-Chlorophenyl)-biguanide hydrochloride | Lopac | 1 microM |
| Tocainide hydrochloride | Lopac | 1 microM |
| DNQX | Lopac | 1 microM |
| Droperidol | Lopac | 1 microM |
| Carvedilol | Lopac | 1 microM |
| Doxylamine succinate | Lopac | 1 microM |
| S(-)-Pindolol | Lopac | 1 microM |
| 5,5-Dimethyl-1-pyrroline-N-oxide | Lopac | 1 microM |
| CK2 Inhibitor 2 | Lopac | 1 microM |
| 5-Carboxamidotryptamine maleate | Lopac | 1 microM |
| 2-Chloroadenosine triphosphate tetrasodium | Lopac | 1 microM |
| Cirazoline hydrochloride | Lopac | 1 microM |
| Dihydroouabain | Lopac | 1 microM |
| L-3,4-Dihydroxyphenylalanine methyl ester hydrochloride | Lopac | 1 microM |
| Dihydroergotamine methanesulfonate | Lopac | 1 microM |
| Desipramine hydrochloride | Lopac | 1 microM |
| (-)-alpha-Methylnorepinephrine | Lopac | 1 microM |
| 2',3'-dideoxycytidine | Lopac | 1 microM |
| Ebastine | Lopac | 1 microM |
| Cetirizine dihydrochloride | Lopac | 1 microM |
| (+)-Cyclazocine | Lopac | 1 microM |
| CGP 20712A methanesulfonate | Lopac | 1 microM |
| Dobutamine hydrochloride | Lopac | 1 microM |
| 1,4-Dideoxy-1,4-imino-D-arabinitol | Lopac | 1 microM |
| Diphenyleneiodonium chloride | Lopac | 1 microM |
| N-Methyl-1-deoxynojirimycin | Lopac | 1 microM |
| Dilazep hydrochloride | Lopac | 1 microM |
| Diacylglycerol Kinase Inhibitor II | Lopac | 1 microM |

| | | |
|---|---|---|
| OXA-22 | Lopac | 1 microM |
| (±)-CGP-12177A hydrochloride | Lopac | 1 microM |
| Capsazepine | Lopac | 1 microM |
| (2S,1'S,2'S)-2-(carboxycyclopropyl)glycine | Lopac | 1 microM |
| Dihydrokainic acid | Lopac | 1 microM |
| 2,4-Dinitrophenyl 2-fluoro-2-deoxy-beta-D-glucopyranoside | Lopac | 1 microM |
| Diphenhydramine hydrochloride | Lopac | 1 microM |
| 5,5-Diphenylhydantoin | Lopac | 1 microM |
| Ganaxolone | Lopac | 1 microM |
| Cambinol | Lopac | 1 microM |
| 8-Cyclopentyl-1,3-dipropylxanthine | Lopac | 1 microM |
| S-(-)-Carbidopa | Lopac | 1 microM |
| Chlormezanone | Lopac | 1 microM |
| CNQX disodium | Lopac | 1 microM |
| Decamethonium dibromide | Lopac | 1 microM |
| D-ribofuranosylbenzimidazole | Lopac | 1 microM |
| 2,3-Butanedione | Lopac | 1 microM |
| N^G,N^G-Dimethylarginine hydrochloride | Lopac | 1 microM |
| 1,7-Dimethylxanthine | Lopac | 1 microM |
| N-Methyldopamine hydrochloride | Lopac | 1 microM |
| 8-Cyclopentyl-1,3-dimethylxanthine | Lopac | 1 microM |
| (±)-Chloro-APB hydrobromide | Lopac | 1 microM |
| 8-(3-Chlorostyryl)caffeine | Lopac | 1 microM |
| CX 546 | Lopac | 1 microM |
| P1,P4-Di(adenosine-5')tetraphosphate triammonium | Lopac | 1 microM |
| SANT-1 | Lopac | 1 microM |
| N,N,N',N'-Tetramethylazodicarboxamide | Lopac | 1 microM |
| Clodronic acid | Lopac | 1 microM |
| 2,3-Dimethoxy-1,4-naphthoquinone | Lopac | 1 microM |
| 1,1-Dimethyl-4-phenyl-piperazinium iodide | Lopac | 1 microM |
| PD 169316 | Lopac | 1 microM |

| | | |
|---|---|---|
| Disopyramide | Lopac | 1 microM |
| Dephostatin | Lopac | 1 microM |
| Diazoxide | Lopac | 1 microM |
| Doxycycline hydrochloride | Lopac | 1 microM |
| R(-)-N-Allylnorapomorphine hydrobromide | Lopac | 1 microM |
| 4-DAMP methiodide | Lopac | 1 microM |
| N,N-Dipropyl-5-carboxamidotryptamine maleate | Lopac | 1 microM |
| Dihydroergocristine methanesulfonate | Lopac | 1 microM |
| Enoximone | Lopac | 1 microM |
| Disopyramide phosphate | Lopac | 1 microM |
| Daidzein | Lopac | 1 microM |
| Imperatorin | Lopac | 1 microM |
| 3,4-Dihydroxyphenylacetic acid | Lopac | 1 microM |
| 6,7-ADTN hydrobromide | Lopac | 1 microM |
| Mephetyl tetrazole | Lopac | 1 microM |
| 1,3-Dipropyl-7-methylxanthine | Lopac | 1 microM |
| 6,7-Dichloroquinoxaline-2,3-dione | Lopac | 1 microM |
| 2,4-Diamino-6-pyrimidinone | Lopac | 1 microM |
| Etoposide | Lopac | 1 microM |
| Demeclocycline hydrochloride | Lopac | 1 microM |
| Cilnidipine | Lopac | 1 microM |
| 3-deazaadenosine | Lopac | 1 microM |
| Dantrolene sodium | Lopac | 1 microM |
| R(-)-Apocodeine hydrochloride | Lopac | 1 microM |
| Icilin | Lopac | 1 microM |
| Domperidone | Lopac | 1 microM |
| 3,7-Dimethyl-1-propargylxanthine | Lopac | 1 microM |
| DL-alpha-Difluoromethylornithine hydrochloride | Lopac | 1 microM |
| ET-18-OCH3 | Lopac | 1 microM |
| Diethylenetriaminepentaacetic acid | Lopac | 1 microM |
| Dicyclomine hydrochloride | Lopac | 1 microM |

| | | |
|---|---|---|
| Cytidine 5'-diphosphocholine sodium salt hydrate | Lopac | 1 microM |
| DCEBIO | Lopac | 1 microM |
| R(-)-Propylnorapomorphine hydrochloride | Lopac | 1 microM |
| (±)-SKF-38393 hydrochloride | Lopac | 1 microM |
| Propofol | Lopac | 1 microM |
| 5,7-Dichlorokynurenic acid | Lopac | 1 microM |
| SCH-28080 | Lopac | 1 microM |
| Etazolate hydrochloride | Lopac | 1 microM |
| Diclofenac sodium | Lopac | 1 microM |
| 3,4-Dichloroisocoumarin | Lopac | 1 microM |
| Danazol | Lopac | 1 microM |
| 1-Deoxynojirimycin hydrochloride | Lopac | 1 microM |
| R(-)-2,10,11-Trihydroxyaporphine hybrobromide | Lopac | 1 microM |
| GBR-12909 dihydrochloride | Lopac | 1 microM |
| Nefiracetam | Lopac | 1 microM |
| 4-Diphenylacetoxy-N-(2-chloroethyl)piperidine hydrochloride | Lopac | 1 microM |
| Venlafaxine hydrochloride | Lopac | 1 microM |
| 7-Cyclopentyl-5-(4-phenoxy)phenyl-7H-pyrrolo[2,3-d]pyrimidin-4-ylamine | Lopac | 1 microM |
| DL-erythro-Dihydrosphingosine | Lopac | 1 microM |
| DBO-83 | Lopac | 1 microM |
| N,N-Dihexyl-2-(4-fluorophenyl)indole-3-acetamide | Lopac | 1 microM |
| L-3,4-Dihydroxyphenylalanine | Lopac | 1 microM |
| R(-)-2,10,11-Trihydroxy-N-propylnoraporphine hydrobromide | Lopac | 1 microM |
| R(+)-SCH-23390 hydrochloride | Lopac | 1 microM |
| R(+)-Butylindazone | Lopac | 1 microM |
| 1,10-Diaminodecane | Lopac | 1 microM |
| Vanillic acid diethylamide | Lopac | 1 microM |
| Emetine dihydrochloride hydrate | Lopac | 1 microM |
| R-(-)-Desmethyldeprenyl hydrochloride | Lopac | 1 microM |
| 7,7-Dimethyl-(5Z,8Z)-eicosadienoic acid | Lopac | 1 microM |
| (R,R)-cis-Diethyl tetrahydro-2,8-chrysenediol | Lopac | 1 microM |

| | | |
|---|---|---|
| Dipyridamole | Lopac | 1 microM |
| Dipropyldopamine hydrobromide | Lopac | 1 microM |
| (±)-DOI hydrochloride | Lopac | 1 microM |
| Eliprodil | Lopac | 1 microM |
| Dihydro-beta-erythroidine hydrobromide | Lopac | 1 microM |
| Epibestatin hydrochloride | Lopac | 1 microM |
| 5'-N-Ethylcarboxamidoadenosine | Lopac | 1 microM |
| 2,2'-Bipyridyl | Lopac | 1 microM |
| (±) trans-U-50488 methanesulfonate | Lopac | 1 microM |
| SP600125 | Lopac | 1 microM |
| Doxazosin mesylate | Lopac | 1 microM |
| AC-93253 iodide | Lopac | 1 microM |
| (±)-2,3-Dichloro-alpha-methylbenzylamine hydrochloride | Lopac | 1 microM |
| 3,5-Dinitrocatechol | Lopac | 1 microM |
| AL-8810 | Lopac | 1 microM |
| Etodolac | Lopac | 1 microM |
| E-64 | Lopac | 1 microM |
| SB 415286 | Lopac | 1 microM |
| rac-2-Ethoxy-3-octadecanamido-1-propylphosphocholine | Lopac | 1 microM |
| (-)-Physostigmine | Lopac | 1 microM |
| S-(-)-Eticlopride hydrochloride | Lopac | 1 microM |
| UCL 2077 | Lopac | 1 microM |
| Fenofibrate | Lopac | 1 microM |
| Forskolin | Lopac | 1 microM |
| Fexofenadine hydrochloride | Lopac | 1 microM |
| N-(3,3-Diphenylpropyl)glycinamide | Lopac | 1 microM |
| L-Canavanine | Lopac | 1 microM |
| S-Ethylisothiourea hydrobromide | Lopac | 1 microM |
| N-Ethylmaleimide | Lopac | 1 microM |
| NBI 27914 | Lopac | 1 microM |
| CCG-4986 | Lopac | 1 microM |

| | | |
|---|---|---|
| Fluvoxamine maleate | Lopac | 1 microM |
| Fenspiride hydrochloride | Lopac | 1 microM |
| Famotidine | Lopac | 1 microM |
| Formoterol | Lopac | 1 microM |
| Glybenclamide | Lopac | 1 microM |
| GW1929 | Lopac | 1 microM |
| Epinastine hydrochloride | Lopac | 1 microM |
| (-)-Epinephrine bitartrate | Lopac | 1 microM |
| beta-Estradiol | Lopac | 1 microM |
| erythro-9-(2-Hydroxy-3-nonyl)adenine hydrochloride | Lopac | 1 microM |
| AS 604850 | Lopac | 1 microM |
| Flumazenil | Lopac | 1 microM |
| FSCPX | Lopac | 1 microM |
| Felodipine | Lopac | 1 microM |
| GW2974 | Lopac | 1 microM |
| GW5074 | Lopac | 1 microM |
| Edrophonium chloride | Lopac | 1 microM |
| EGTA | Lopac | 1 microM |
| Estrone | Lopac | 1 microM |
| Alinidine | Lopac | 1 microM |
| Furegrelate sodium | Lopac | 1 microM |
| Genipin | Lopac | 1 microM |
| NS8593 hydrochloride | Lopac | 1 microM |
| Fluspirilene | Lopac | 1 microM |
| Guanfacine hydrochloride | Lopac | 1 microM |
| Genistein | Lopac | 1 microM |
| Efaroxan hydrochloride | Lopac | 1 microM |
| (±)-Epinephrine hydrochloride | Lopac | 1 microM |
| Phenserine | Lopac | 1 microM |
| Felbamate | Lopac | 1 microM |
| Fiduxosin hydrochloride | Lopac | 1 microM |

| | | |
|---|---|---|
| Fusaric acid | Lopac | 1 microM |
| Flunarizine dihydrochloride | Lopac | 1 microM |
| cis-(Z)-Flupenthixol dihydrochloride | Lopac | 1 microM |
| L-Glutamic acid hydrochloride | Lopac | 1 microM |
| GW7647 | Lopac | 1 microM |
| Ellipticine | Lopac | 1 microM |
| Ethosuximide | Lopac | 1 microM |
| N-Methyl-beta-carboline-3-carboxamide | Lopac | 1 microM |
| Fusidic acid sodium | Lopac | 1 microM |
| Furosemide | Lopac | 1 microM |
| 5-Fluorouracil | Lopac | 1 microM |
| 5-fluoro-5'-deoxyuridine | Lopac | 1 microM |
| Furafylline | Lopac | 1 microM |
| Ganciclovir | Lopac | 1 microM |
| alpha-Guanidinoglutaric acid | Lopac | 1 microM |
| Ebselen | Lopac | 1 microM |
| JX401 | Lopac | 1 microM |
| DPO-1 | Lopac | 1 microM |
| Fenoterol hydrobromide | Lopac | 1 microM |
| p-Fluoro-L-phenylalanine | Lopac | 1 microM |
| Flecainide acetate | Lopac | 1 microM |
| Flupirtine maleate | Lopac | 1 microM |
| FPL 64176 | Lopac | 1 microM |
| L-Glutamine | Lopac | 1 microM |
| Gallamine triethiodide | Lopac | 1 microM |
| rac-2-Ethoxy-3-hexadecanamido-1-propylphosphocholine | Lopac | 1 microM |
| Emodin | Lopac | 1 microM |
| (-)-Eseroline fumarate | Lopac | 1 microM |
| S-(+)-Fluoxetine hydrochloride | Lopac | 1 microM |
| Fluphenazine dihydrochloride | Lopac | 1 microM |
| Fenoldopam bromide | Lopac | 1 microM |

| | | |
|---|---|---|
| Flutamide | Lopac | 1 microM |
| Fluoxetine hydrochloride | Lopac | 1 microM |
| Guanidinyl-naltrindole di-trifluoroacetate | Lopac | 1 microM |
| GBR-12935 dihydrochloride | Lopac | 1 microM |
| Isoguvacine hydrochloride | Lopac | 1 microM |
| Paliperidone | Lopac | 1 microM |
| MHPG piperazine | Lopac | 1 microM |
| DL-threo-beta-hydroxyaspartic acid | Lopac | 1 microM |
| 17alpha-hydroxyprogesterone | Lopac | 1 microM |
| L-Histidine hydrochloride | Lopac | 1 microM |
| L-Hyoscyamine | Lopac | 1 microM |
| 4-Hydroxybenzhydrazide | Lopac | 1 microM |
| R-(+)-7-Hydroxy-DPAT hydrobromide | Lopac | 1 microM |
| Iodoacetamide | Lopac | 1 microM |
| Guvacine hydrochloride | Lopac | 1 microM |
| GR-89696 fumarate | Lopac | 1 microM |
| Hypotaurine | Lopac | 1 microM |
| Ciproxifan hydrochloride | Lopac | 1 microM |
| 1,3,5-tris(4-hydroxyphenyl)-4-propyl-1H-pyrazole | Lopac | 1 microM |
| (±)-8-Hydroxy-DPAT hydrobromide | Lopac | 1 microM |
| Hydroquinone | Lopac | 1 microM |
| Hemicholinium-3 | Lopac | 1 microM |
| Sematilide monohydrochloride monohydrate | Lopac | 1 microM |
| HA-100 | Lopac | 1 microM |
| (±)-AMPA hydrobromide | Lopac | 1 microM |
| Gabapentin | Lopac | 1 microM |
| Haloperidol | Lopac | 1 microM |
| Hydroxytacrine maleate | Lopac | 1 microM |
| 1-(4-Hydroxybenzyl)imidazole-2-thiol | Lopac | 1 microM |
| Dopamine hydrochloride | Lopac | 1 microM |
| BU99006 | Lopac | 1 microM |

| | | |
|---|---|---|
| HA-1004 hydrochloride | Lopac | 1 microM |
| IEM-1460 | Lopac | 1 microM |
| Ipratropium bromide | Lopac | 1 microM |
| Muscimol hydrobromide | Lopac | 1 microM |
| DL-Homatropine hydrobromide | Lopac | 1 microM |
| Hydralazine hydrochloride | Lopac | 1 microM |
| Hydrocortisone | Lopac | 1 microM |
| Histamine dihydrochloride | Lopac | 1 microM |
| Hydroxyurea | Lopac | 1 microM |
| MNS | Lopac | 1 microM |
| N-Methylhistaprodifen dioxalate salt | Lopac | 1 microM |
| Ibudilast | Lopac | 1 microM |
| Idarubicin | Lopac | 1 microM |
| Guanabenz acetate | Lopac | 1 microM |
| (±)-Vanillylmandelic acid | Lopac | 1 microM |
| 4-Imidazolemethanol hydrochloride | Lopac | 1 microM |
| Lithium Chloride | Lopac | 1 microM |
| Harmane | Lopac | 1 microM |
| (+)-Hydrastine | Lopac | 1 microM |
| Serotonin hydrochloride | Lopac | 1 microM |
| Hexahydro-sila-difenidol hydrochloride, p-fluoro analog | Lopac | 1 microM |
| Imidazole-4-acetic acid hydrochloride | Lopac | 1 microM |
| Metolazone | Lopac | 1 microM |
| JFD00244 | Lopac | 1 microM |
| 6-Hydroxymelatonin | Lopac | 1 microM |
| Hexamethonium dichloride | Lopac | 1 microM |
| Hydrochlorothiazide | Lopac | 1 microM |
| JS-K | Lopac | 1 microM |
| (±)-7-Hydroxy-DPAT hydrobromide | Lopac | 1 microM |
| L-165,041 | Lopac | 1 microM |
| Histamine, R(-)-alpha-methyl-, dihydrochloride | Lopac | 1 microM |

| | | |
|---|---|---|
| CPNQ | Lopac | 1 microM |
| GR 55562 dihydrobromide | Lopac | 1 microM |
| Glipizide | Lopac | 1 microM |
| Hexamethonium bromide | Lopac | 1 microM |
| CCG-2046 | Lopac | 1 microM |
| SB 218795 | Lopac | 1 microM |
| Retinoic acid p-hydroxyanilide | Lopac | 1 microM |
| MHPG sulfate potassium | Lopac | 1 microM |
| 5-Hydroxy-L-tryptophan | Lopac | 1 microM |
| 5-hydroxydecanoic acid sodium | Lopac | 1 microM |
| NSC 95397 | Lopac | 1 microM |
| IMID-4F hydrochloride | Lopac | 1 microM |
| GYKI 52466 hydrochloride | Lopac | 1 microM |
| 4-Hydroxy-3-methoxyphenylacetic acid | Lopac | 1 microM |
| 6-Hydroxy-DL-DOPA | Lopac | 1 microM |
| Hispidin | Lopac | 1 microM |
| ABT-702 dihydrochloride | Lopac | 1 microM |
| 5-Hydroxyindolacetic acid | Lopac | 1 microM |
| Hydroxylamine hydrochloride | Lopac | 1 microM |
| R-(+)-8-Hydroxy-DPAT hydrobromide | Lopac | 1 microM |
| Imazodan | Lopac | 1 microM |
| R(-)-Isoproterenol (+)-bitartrate | Lopac | 1 microM |
| ML-7 | Lopac | 1 microM |
| 3-Isobutyl-1-methylxanthine | Lopac | 1 microM |
| Iproniazid phosphate | Lopac | 1 microM |
| m-Iodobenzylguanidine hemisulfate | Lopac | 1 microM |
| Imetit dihydrobromide | Lopac | 1 microM |
| JL-18 | Lopac | 1 microM |
| Kenpaullone | Lopac | 1 microM |
| LY-367,265 | Lopac | 1 microM |
| Leflunomide | Lopac | 1 microM |

| | | |
|---|---|---|
| LFM-A13 | Lopac | 1 microM |
| (±)-Ibotenic acid | Lopac | 1 microM |
| Idazoxan hydrochloride | Lopac | 1 microM |
| S(+)-Isoproterenol (+)-bitartrate | Lopac | 1 microM |
| S(+)-Ibuprofen | Lopac | 1 microM |
| 1,5-Isoquinolinediol | Lopac | 1 microM |
| Kainic acid | Lopac | 1 microM |
| U-73343 | Lopac | 1 microM |
| LY-310,762 hydrochloride | Lopac | 1 microM |
| VER-3323 hemifumarate salt | Lopac | 1 microM |
| NNC 55-0396 | Lopac | 1 microM |
| Ifenprodil tartrate | Lopac | 1 microM |
| 1-(5-Isoquinolinylsulfonyl)-3-methylpiperazine dihydrochloride | Lopac | 1 microM |
| L-N6-(1-Iminoethyl)lysine hydrochloride | Lopac | 1 microM |
| p-Iodoclonidine hydrochloride | Lopac | 1 microM |
| Molindone hydrochloride | Lopac | 1 microM |
| Ketoconazole | Lopac | 1 microM |
| L-701,324 | Lopac | 1 microM |
| Olvanil | Lopac | 1 microM |
| Lidocaine hydrochloride | Lopac | 1 microM |
| Ro 90-7501 | Lopac | 1 microM |
| Isotharine mesylate | Lopac | 1 microM |
| (-)-Isoproterenol hydrochloride | Lopac | 1 microM |
| 3-Iodo-L-tyrosine | Lopac | 1 microM |
| R(+)-IAA-94 | Lopac | 1 microM |
| IB-MECA | Lopac | 1 microM |
| Ketorolac tris salt | Lopac | 1 microM |
| loxoprofen | Lopac | 1 microM |
| Lomefloxacin hydrochloride | Lopac | 1 microM |
| Lidocaine N-ethyl bromide quaternary salt | Lopac | 1 microM |
| Loratadine | Lopac | 1 microM |

| | | |
|---|---|---|
| Isoliquiritigenin | Lopac | 1 microM |
| 1-(5-Isoquinolinylsulfonyl)-2-methylpiperazine dihydrochloride | Lopac | 1 microM |
| Cibenzoline succinate | Lopac | 1 microM |
| Indatraline hydrochloride | Lopac | 1 microM |
| Aurothioglucose | Lopac | 1 microM |
| Ketoprofen | Lopac | 1 microM |
| Labetalol hydrochloride | Lopac | 1 microM |
| Lamotrigine | Lopac | 1 microM |
| L-Leucinethiol, oxidized dihydrochloride | Lopac | 1 microM |
| (-)-Tetramisole hydrochloride | Lopac | 1 microM |
| (±)-Ibuprofen | Lopac | 1 microM |
| Indomethacin | Lopac | 1 microM |
| Ivermectin | Lopac | 1 microM |
| Iofetamine hydrochloride | Lopac | 1 microM |
| 3-(1H-Imidazol-4-yl)propyl di(p-fluorophenyl)methyl ether hydrochloride | Lopac | 1 microM |
| K 185 | Lopac | 1 microM |
| L-162,313 | Lopac | 1 microM |
| alpha-Lobeline hydrochloride | Lopac | 1 microM |
| LE 300 | Lopac | 1 microM |
| L-655,708 | Lopac | 1 microM |
| SD-169 | Lopac | 1 microM |
| Imipramine hydrochloride | Lopac | 1 microM |
| Imiloxan hydrochloride | Lopac | 1 microM |
| ICI 204,448 hydrochloride | Lopac | 1 microM |
| Isonipecotic acid | Lopac | 1 microM |
| Ketotifen fumarate | Lopac | 1 microM |
| CyPPA | Lopac | 1 microM |
| Loperamide hydrochloride | Lopac | 1 microM |
| Lansoprazole | Lopac | 1 microM |
| LY-294,002 hydrochloride | Lopac | 1 microM |
| (±)-Isoproterenol hydrochloride | Lopac | 1 microM |

| | | |
|---|---|---|
| Isoxanthopterin | Lopac | 1 microM |
| Stevioside | Lopac | 1 microM |
| SB-525334 | Lopac | 1 microM |
| JWH-015 | Lopac | 1 microM |
| Kynurenic acid | Lopac | 1 microM |
| beta-Lapachone | Lopac | 1 microM |
| Lonidamine | Lopac | 1 microM |
| L-687,384 hydrochloride | Lopac | 1 microM |
| Loxapine succinate | Lopac | 1 microM |
| TMPH hydrochloride | Lopac | 1 microM |
| L-750,667 trihydrochloride | Lopac | 1 microM |
| 4-Methylpyrazole hydrochloride | Lopac | 1 microM |
| p-MPPI hydrochloride | Lopac | 1 microM |
| Molsidomine | Lopac | 1 microM |
| Metergoline | Lopac | 1 microM |
| Meclofenamic acid sodium | Lopac | 1 microM |
| (±)-Metoprolol (+)-tartrate | Lopac | 1 microM |
| GW405833 hydrochloride | Lopac | 1 microM |
| MDL 28170 | Lopac | 1 microM |
| Lorglumide sodium | Lopac | 1 microM |
| Linopirdine | Lopac | 1 microM |
| Nocodazole | Lopac | 1 microM |
| Metaproterenol hemisulfate | Lopac | 1 microM |
| BBMP | Lopac | 1 microM |
| (-)-cis-(1S,2R)-U-50488 tartrate | Lopac | 1 microM |
| Milrinone | Lopac | 1 microM |
| 6-Methyl-2-(phenylethynyl)pyridine hydrochloride | Lopac | 1 microM |
| 2-methoxyestradiol | Lopac | 1 microM |
| Myricetin | Lopac | 1 microM |
| CHM-1 hydrate | Lopac | 1 microM |
| L-741,626 | Lopac | 1 microM |

| | | |
|---|---|---|
| N-omega-Methyl-5-hydroxytryptamine oxalate salt | Lopac | 1 microM |
| Mianserin hydrochloride | Lopac | 1 microM |
| Mizoribine | Lopac | 1 microM |
| Clorgyline hydrochloride | Lopac | 1 microM |
| (±)-alpha-Methyl-4-carboxyphenylglycine | Lopac | 1 microM |
| Mibefradil dihydrochloride | Lopac | 1 microM |
| Cysteamine hydrochloride | Lopac | 1 microM |
| NG-Monomethyl-L-arginine acetate | Lopac | 1 microM |
| cis(+/-)-8-OH-PBZI hydrobromide | Lopac | 1 microM |
| L-733,060 hydrochloride | Lopac | 1 microM |
| Moxonidine hydrochloride | Lopac | 1 microM |
| Mevastatin | Lopac | 1 microM |
| S-Methylisothiourea hemisulfate | Lopac | 1 microM |
| MRS 2179 | Lopac | 1 microM |
| 1-Methylhistamine dihydrochloride | Lopac | 1 microM |
| N6-Methyladenosine | Lopac | 1 microM |
| alpha,beta-Methylene adenosine 5'-triphosphate dilithium | Lopac | 1 microM |
| MK-912 | Lopac | 1 microM |
| BW 723C86 | Lopac | 1 microM |
| Metoclopramide hydrochloride | Lopac | 1 microM |
| MRS 1845 | Lopac | 1 microM |
| 8-Methoxymethyl-3-isobutyl-1-methylxanthine | Lopac | 1 microM |
| MG 624 | Lopac | 1 microM |
| Meloxicam sodium | Lopac | 1 microM |
| Moxisylyte hydrochloride | Lopac | 1 microM |
| (S)-MAP4 hydrochloride | Lopac | 1 microM |
| Methoxamine hydrochloride | Lopac | 1 microM |
| (±)-3-(3,4-dihydroxyphenyl)-2-methyl-DL-alanine | Lopac | 1 microM |
| Levallorphan tartrate | Lopac | 1 microM |
| R(-)-Me5 | Lopac | 1 microM |
| BIO | Lopac | 1 microM |

| | | |
|---|---|---|
| MK-886 | Lopac | 1 microM |
| N-Methyl-D-aspartic acid | Lopac | 1 microM |
| Morin | Lopac | 1 microM |
| S-Methyl-L-thiocitrulline acetate | Lopac | 1 microM |
| (±)-Methoxyverapamil hydrochloride | Lopac | 1 microM |
| Mitoxantrone | Lopac | 1 microM |
| MRS 2159 | Lopac | 1 microM |
| AFMK | Lopac | 1 microM |
| Dihydrocapsaicin | Lopac | 1 microM |
| MRS 1523 | Lopac | 1 microM |
| Mexiletene hydrochloride | Lopac | 1 microM |
| alpha-Methyl-DL-tyrosine methyl ester hydrochloride | Lopac | 1 microM |
| Minoxidil | Lopac | 1 microM |
| Melatonin | Lopac | 1 microM |
| Metrazoline oxalate | Lopac | 1 microM |
| O-Methylserotonin hydrochloride | Lopac | 1 microM |
| GR 127935 hydrochloride hydrate | Lopac | 1 microM |
| L-745,870 hydrochloride | Lopac | 1 microM |
| (-)-Naproxen sodium | Lopac | 1 microM |
| Melphalan | Lopac | 1 microM |
| Methylergonovine maleate | Lopac | 1 microM |
| ML 10302 | Lopac | 1 microM |
| Rufinamide | Lopac | 1 microM |
| L-Methionine sulfoximine | Lopac | 1 microM |
| GW9662 | Lopac | 1 microM |
| Se-(methyl)selenocysteine hydrochloride | Lopac | 1 microM |
| 2,6-Difluoro-4-[2-(phenylsulfonylamino)ethylthio]phenoxyacetamide | Lopac | 1 microM |
| Mifepristone | Lopac | 1 microM |
| Minocycline hydrochloride | Lopac | 1 microM |
| (-)-MK-801 hydrogen maleate | Lopac | 1 microM |
| Methiothepin mesylate | Lopac | 1 microM |

| | | |
|---|---|---|
| MDL 105,519 | Lopac | 1 microM |
| nor-Binaltorphimine dihydrochloride | Lopac | 1 microM |
| NCS-356 | Lopac | 1 microM |
| (-)-Nicotine hydrogen tartrate salt | Lopac | 1 microM |
| Nicardipine hydrochloride | Lopac | 1 microM |
| NF 023 | Lopac | 1 microM |
| L-alpha-Methyl-p-tyrosine | Lopac | 1 microM |
| Maprotiline hydrochloride | Lopac | 1 microM |
| 2-Methyl-5-hydroxytryptamine maleate | Lopac | 1 microM |
| Nemadipine-A | Lopac | 1 microM |
| Metrifudil | Lopac | 1 microM |
| Neostigmine bromide | Lopac | 1 microM |
| S-Nitrosoglutathione | Lopac | 1 microM |
| NG-Nitro-L-arginine | Lopac | 1 microM |
| Nifedipine | Lopac | 1 microM |
| Nimustine hydrochloride | Lopac | 1 microM |
| SB-215505 | Lopac | 1 microM |
| H-8 dihydrochloride | Lopac | 1 microM |
| alpha-Methyl-5-hydroxytryptamine maleate | Lopac | 1 microM |
| Moclobemide | Lopac | 1 microM |
| p-MPPF dihydrochloride | Lopac | 1 microM |
| CR 2249 | Lopac | 1 microM |
| NCS-382 | Lopac | 1 microM |
| Naphazoline hydrochloride | Lopac | 1 microM |
| Naloxone hydrochloride | Lopac | 1 microM |
| Norcantharidin | Lopac | 1 microM |
| 1-Methylimidazole | Lopac | 1 microM |
| Proglumide | Lopac | 1 microM |
| Metolazone | Lopac | 1 microM |
| MDL 26,630 trihydrochloride | Lopac | 1 microM |
| Levetiracetam | Lopac | 1 microM |

| | | |
|---|---|---|
| S-(4-Nitrobenzyl)-6-thioinosine | Lopac | 1 microM |
| Nalidixic acid sodium | Lopac | 1 microM |
| 3-Nitropropionic acid | Lopac | 1 microM |
| 7-Nitroindazole | Lopac | 1 microM |
| Noscapine hydrchloride | Lopac | 1 microM |
| Mecamylamine hydrochloride | Lopac | 1 microM |
| Fenobam | Lopac | 1 microM |
| DFB | Lopac | 1 microM |
| ZM 39923 hydrochloride | Lopac | 1 microM |
| Niflumic acid | Lopac | 1 microM |
| Naltrexone hydrochloride | Lopac | 1 microM |
| Gossypol | Lopac | 1 microM |
| NG-Nitro-L-arginine methyl ester hydrochloride | Lopac | 1 microM |
| NS 521 oxalate | Lopac | 1 microM |
| (+)-Nicotine (+)-di-p-toluoyl tartrate | Lopac | 1 microM |
| Methapyrilene hydrochloride | Lopac | 1 microM |
| (±)-Muscarine chloride | Lopac | 1 microM |
| L-alpha-Methyl DOPA | Lopac | 1 microM |
| 3-Morpholinosydnonimine hydrochloride | Lopac | 1 microM |
| Nimesulide | Lopac | 1 microM |
| S-Nitroso-N-acetylpenicillamine | Lopac | 1 microM |
| 5-Nitro-2-(3-phenylpropylamino)benzoic acid | Lopac | 1 microM |
| (±)-Normetanephrine hydrochloride | Lopac | 1 microM |
| CI-976 | Lopac | 1 microM |
| Naltrindole hydrochloride | Lopac | 1 microM |
| Memantine hydrochloride | Lopac | 1 microM |
| Methoctramine tetrahydrochloride | Lopac | 1 microM |
| Methysergide maleate | Lopac | 1 microM |
| 3-Methoxy-morphanin hydrochloride | Lopac | 1 microM |
| Nialamide | Lopac | 1 microM |
| Niclosamide | Lopac | 1 microM |

| | | |
|---|---|---|
| AMN082 | Lopac | 1 microM |
| Nortriptyline hydrochloride | Lopac | 1 microM |
| 6-Nitroso-1,2-benzopyrone | Lopac | 1 microM |
| Sertraline hydrochloride | Lopac | 1 microM |
| Me-3,4-dephostatin | Lopac | 1 microM |
| (+)-MK-801 hydrogen maleate | Lopac | 1 microM |
| Ethopropazine hydrochloride | Lopac | 1 microM |
| S15535 | Lopac | 1 microM |
| Nomifensine maleate | Lopac | 1 microM |
| NAN-190 hydrobromide | Lopac | 1 microM |
| Nordihydroguaiaretic acid from Larrea divaricata (creosote bush) | Lopac | 1 microM |
| NADPH tetrasodium | Lopac | 1 microM |
| Nilutamide | Lopac | 1 microM |
| NO-711 hydrochloride | Lopac | 1 microM |
| Nitrendipine | Lopac | 1 microM |
| Naloxone benzoylhydrazone | Lopac | 1 microM |
| Olomoucine | Lopac | 1 microM |
| Orphenadrine hydrochloride | Lopac | 1 microM |
| (±)-Octoclothepin maleate | Lopac | 1 microM |
| O-Phospho-L-serine | Lopac | 1 microM |
| Pancuronium bromide | Lopac | 1 microM |
| Pentolinium di[L(+)-tartrate] | Lopac | 1 microM |
| Valproic acid sodium | Lopac | 1 microM |
| Pyrilamine maleate | Lopac | 1 microM |
| Nimodipine | Lopac | 1 microM |
| NS-1619 | Lopac | 1 microM |
| Oleic Acid | Lopac | 1 microM |
| TG003 | Lopac | 1 microM |
| Progesterone | Lopac | 1 microM |
| (±)-Propranolol hydrochloride | Lopac | 1 microM |
| 3-alpha,21-Dihydroxy-5-alpha-pregnan-20-one | Lopac | 1 microM |

| | | |
|---|---|---|
| 1-Phenyl-3-(2-thiazolyl)-2-thiourea | Lopac | 1 microM |
| Promethazine hydrochloride | Lopac | 1 microM |
| Piroxicam | Lopac | 1 microM |
| Nisoxetine hydrochloride | Lopac | 1 microM |
| Naloxonazine dihydrochloride | Lopac | 1 microM |
| Oxymetazoline hydrochloride | Lopac | 1 microM |
| Ofloxacin | Lopac | 1 microM |
| Palmitoylethanolamide | Lopac | 1 microM |
| SKF-525A hydrochloride | Lopac | 1 microM |
| Pirfenidone | Lopac | 1 microM |
| Thiolactomycin | Lopac | 1 microM |
| Praziquantel | Lopac | 1 microM |
| 3-n-Propylxanthine | Lopac | 1 microM |
| Nylidrin hydrochloride | Lopac | 1 microM |
| NBQX disodium | Lopac | 1 microM |
| Sodium Oxamate | Lopac | 1 microM |
| Oxotremorine sesquifumarate salt | Lopac | 1 microM |
| Piceatannol | Lopac | 1 microM |
| Picrotoxin | Lopac | 1 microM |
| 1,3-Dimethyl-8-phenylxanthine | Lopac | 1 microM |
| Cisplatin | Lopac | 1 microM |
| Propafenone hydrochloride | Lopac | 1 microM |
| Phenylephrine hydrochloride | Lopac | 1 microM |
| SB 242084 dihydrochloride hydrate | Lopac | 1 microM |
| NS 2028 | Lopac | 1 microM |
| Oxybutynin Chloride | Lopac | 1 microM |
| Trandolapril | Lopac | 1 microM |
| Pentamidine isethionate | Lopac | 1 microM |
| LP44 | Lopac | 1 microM |
| PRE-084 | Lopac | 1 microM |
| Podophyllotoxin | Lopac | 1 microM |

| | | |
|---|---|---|
| 5alpha-Pregnan-3alpha-ol-11,20-dione | Lopac | 1 microM |
| Perphenazine | Lopac | 1 microM |
| Naltriben methanesulfonate | Lopac | 1 microM |
| (±)-Octopamine hydrochloride | Lopac | 1 microM |
| Oxiracetam | Lopac | 1 microM |
| SB 216763 | Lopac | 1 microM |
| TBB | Lopac | 1 microM |
| Pentoxifylline | Lopac | 1 microM |
| PPNDS tetrasodium | Lopac | 1 microM |
| SU 9516 | Lopac | 1 microM |
| PNU-282987 | Lopac | 1 microM |
| Pentylenetetrazole | Lopac | 1 microM |
| Naftopidil dihydrochloride | Lopac | 1 microM |
| N-Oleoylethanolamine | Lopac | 1 microM |
| Oxaprozin | Lopac | 1 microM |
| Parthenolide | Lopac | 1 microM |
| Pimozide | Lopac | 1 microM |
| PD 404,182 | Lopac | 1 microM |
| Palmitoyl-DL-Carnitine chloride | Lopac | 1 microM |
| Piracetam | Lopac | 1 microM |
| (+)-Pilocarpine hydrochloride | Lopac | 1 microM |
| Bisoprolol hemifumarate salt | Lopac | 1 microM |
| Oxolinic acid | Lopac | 1 microM |
| ODQ | Lopac | 1 microM |
| Oxotremorine methiodide | Lopac | 1 microM |
| Pindolol | Lopac | 1 microM |
| L-Glutamic acid, N-phthaloyl- | Lopac | 1 microM |
| Papaverine hydrochloride | Lopac | 1 microM |
| R(-)-N6-(2-Phenylisopropyl)adenosine | Lopac | 1 microM |
| Phosphomycin disodium | Lopac | 1 microM |
| Pilocarpine nitrate | Lopac | 1 microM |

| | | |
|---|---|---|
| Promazine hydrochloride | Lopac | 1 microM |
| Pirenzepine dihydrochloride | Lopac | 1 microM |
| ARP 101 | Lopac | 1 microM |
| (±)-cis-Piperidine-2,3-dicarboxylic acid | Lopac | 1 microM |
| Piribedil maleate | Lopac | 1 microM |
| Procaine hydrochloride | Lopac | 1 microM |
| Phaclofen | Lopac | 1 microM |
| Pregnenolone sulfate sodium | Lopac | 1 microM |
| PD 168,077 maleate | Lopac | 1 microM |
| Quinacrine dihydrochloride | Lopac | 1 microM |
| Phenelzine sulfate | Lopac | 1 microM |
| Putrescine dihydrochloride | Lopac | 1 microM |
| Protoporphyrin IX disodium | Lopac | 1 microM |
| Protriptyline hydrochloride | Lopac | 1 microM |
| Paromomycin sulfate | Lopac | 1 microM |
| 2-Phenylaminoadenosine | Lopac | 1 microM |
| BF-170 hydrochloride | Lopac | 1 microM |
| PPADS | Lopac | 1 microM |
| SU 6656 | Lopac | 1 microM |
| Quazinone | Lopac | 1 microM |
| Pheniramine maleate | Lopac | 1 microM |
| Phentolamine mesylate | Lopac | 1 microM |
| 1,4-PBIT dihydrobromide | Lopac | 1 microM |
| Pergolide methanesulfonate | Lopac | 1 microM |
| 1,10-Phenanthroline monohydrate | Lopac | 1 microM |
| R(+)-3PPP hydrochloride | Lopac | 1 microM |
| 1-Phenylbiguanide | Lopac | 1 microM |
| S(+)-PD 128,907 hydrochloride | Lopac | 1 microM |
| Quinolinic acid | Lopac | 1 microM |
| (-)-Quinpirole hydrochloride | Lopac | 1 microM |
| Phosphonoacetic acid | Lopac | 1 microM |

| | | |
|---|---|---|
| Propionylpromazine hydrochloride | Lopac | 1 microM |
| Phenylbutazone | Lopac | 1 microM |
| 6(5H)-Phenanthridinone | Lopac | 1 microM |
| Procainamide hydrochloride | Lopac | 1 microM |
| S(-)-3PPP hydrochloride | Lopac | 1 microM |
| SID7969543 | Lopac | 1 microM |
| Phenamil methanesulfonate | Lopac | 1 microM |
| Quercetin dihydrate | Lopac | 1 microM |
| Quipazine, N-methyl-, dimaleate | Lopac | 1 microM |
| (-)-Perillic acid | Lopac | 1 microM |
| Prazosin hydrochloride | Lopac | 1 microM |
| Picotamide | Lopac | 1 microM |
| 5alpha-Pregnan-3alpha-ol-20-one | Lopac | 1 microM |
| Prilocaine hydrochloride | Lopac | 1 microM |
| (±)-PPHT hydrochloride | Lopac | 1 microM |
| Pirenperone | Lopac | 1 microM |
| Phenylbenzene-omega-phosphono-alpha-amino acid | Lopac | 1 microM |
| Quinidine sulfate | Lopac | 1 microM |
| Quipazine, 6-nitro-, maleate | Lopac | 1 microM |
| Pyrazinecarboxamide | Lopac | 1 microM |
| Phloretin | Lopac | 1 microM |
| Tranylcypromine hydrochloride | Lopac | 1 microM |
| Propantheline bromide | Lopac | 1 microM |
| Propentofylline | Lopac | 1 microM |
| Org 27569 | Lopac | 1 microM |
| IC 261 | Lopac | 1 microM |
| Bay 11-7082 | Lopac | 1 microM |
| SMER28 | Lopac | 1 microM |
| Quinelorane dihydrochloride | Lopac | 1 microM |
| Primidone | Lopac | 1 microM |
| Pargyline hydrochloride | Lopac | 1 microM |

| | | |
|---|---|---|
| (S)-Propranolol hydrochloride | Lopac | 1 microM |
| K114 | Lopac | 1 microM |
| Ziprasidone hydrochloride monohydrate | Lopac | 1 microM |
| Bicalutamide (CDX) | Lopac | 1 microM |
| A3 hydrochloride | Lopac | 1 microM |
| PD 98,059 | Lopac | 1 microM |
| Quinine sulfate | Lopac | 1 microM |
| (±)-Quinpirole dihydrochloride | Lopac | 1 microM |
| (±)-threo-1-Phenyl-2-decanoylamino-3-morpholino-1-propanol hydrochloride | Lopac | 1 microM |
| Phorbol 12-myristate 13-acetate | Lopac | 1 microM |
| Ammonium pyrrolidinedithiocarbamate | Lopac | 1 microM |
| Prochlorperazine dimaleate | Lopac | 1 microM |
| Pyridostigmine bromide | Lopac | 1 microM |
| N6-Phenyladenosine | Lopac | 1 microM |
| Pinacidil | Lopac | 1 microM |
| (±)-PD 128,907 hydrochloride | Lopac | 1 microM |
| (+)-Quisqualic acid | Lopac | 1 microM |
| Cortexolone | Lopac | 1 microM |
| Ritodrine hydrochloride | Lopac | 1 microM |
| REV 5901 | Lopac | 1 microM |
| Ro 8-4304 | Lopac | 1 microM |
| Ro 41-0960 | Lopac | 1 microM |
| Ro 04-6790 dihydrochloride | Lopac | 1 microM |
| ST-148 | Lopac | 1 microM |
| Spermidine trihydrochloride | Lopac | 1 microM |
| Stattic | Lopac | 1 microM |
| IRAK-1/4 Inhibitor I | Lopac | 1 microM |
| (±)-Sulpiride | Lopac | 1 microM |
| Raloxifene hydrochloride | Lopac | 1 microM |
| Rottlerin | Lopac | 1 microM |
| RX 821002 hydrochloride | Lopac | 1 microM |

| | | |
|---|---|---|
| Reactive Blue 2 | Lopac | 1 microM |
| (±)-Sotalol hydrochloride | Lopac | 1 microM |
| SKF 86466 | Lopac | 1 microM |
| SNC80 | Lopac | 1 microM |
| N-Oleoyldopamine | Lopac | 1 microM |
| SB 269970 hydrochloride | Lopac | 1 microM |
| CV-3988 | Lopac | 1 microM |
| Retinoic acid | Lopac | 1 microM |
| Ranolazine dihydrochloride | Lopac | 1 microM |
| Ribavirin | Lopac | 1 microM |
| Riluzole | Lopac | 1 microM |
| SB-366791 | Lopac | 1 microM |
| SR 57227A | Lopac | 1 microM |
| SKF 83959 hydrobromide | Lopac | 1 microM |
| Spironolactone | Lopac | 1 microM |
| Spiperone hydrochloride | Lopac | 1 microM |
| Sulindac | Lopac | 1 microM |
| Ruthenium red | Lopac | 1 microM |
| Rolipram | Lopac | 1 microM |
| Ranitidine hydrochloride | Lopac | 1 microM |
| Steviol | Lopac | 1 microM |
| Sodium nitroprusside dihydrate | Lopac | 1 microM |
| (-)-Scopolamine hydrobromide | Lopac | 1 microM |
| Spermine tetrahydrochloride | Lopac | 1 microM |
| SCH-202676 hydrobromide | Lopac | 1 microM |
| SR 2640 | Lopac | 1 microM |
| Succinylcholine chloride | Lopac | 1 microM |
| 13-cis-retinoic acid | Lopac | 1 microM |
| Ro 25-6981 hydrochloride | Lopac | 1 microM |
| Ritanserin | Lopac | 1 microM |
| S(+)-Raclopride L-tartrate | Lopac | 1 microM |

| | | |
|---|---|---|
| (±)-Synephrine | Lopac | 1 microM |
| Tetrabenazine | Lopac | 1 microM |
| SKF 75670 hydrobromide | Lopac | 1 microM |
| D-Serine | Lopac | 1 microM |
| (-)-Sulpiride | Lopac | 1 microM |
| Salbutamol | Lopac | 1 microM |
| Rutaecarpine | Lopac | 1 microM |
| Phosphoramidon disodium | Lopac | 1 microM |
| Rauwolscine hydrochloride | Lopac | 1 microM |
| Sobuzoxane | Lopac | 1 microM |
| Sulfaphenazole | Lopac | 1 microM |
| Semicarbazide hydrochloride | Lopac | 1 microM |
| SC 19220 | Lopac | 1 microM |
| Albuterol hemisulfate | Lopac | 1 microM |
| SKF 96365 | Lopac | 1 microM |
| Salmeterol xinafoate | Lopac | 1 microM |
| Ropinirole hydrochloride | Lopac | 1 microM |
| BIA 2-093 | Lopac | 1 microM |
| Ibandronate sodium | Lopac | 1 microM |
| Rilmenidine hemifumarate | Lopac | 1 microM |
| 1-(4-Hexyphenyl)-2-propane-1-one | Lopac | 1 microM |
| (-)-Scopolamine methyl nitrate | Lopac | 1 microM |
| SKF 89626 | Lopac | 1 microM |
| Sanguinarine chloride | Lopac | 1 microM |
| (-)-Scopolamine,n-Butyl-, bromide | Lopac | 1 microM |
| SU 5416 | Lopac | 1 microM |
| Resveratrol | Lopac | 1 microM |
| Rotenone | Lopac | 1 microM |
| Ro 41-1049 hydrochloride | Lopac | 1 microM |
| R(-)-Denopamine | Lopac | 1 microM |
| Sulindac sulfone | Lopac | 1 microM |

| | | |
|---|---|---|
| DL-Stearoylcarnitine chloride | Lopac | 1 microM |
| AGK2 | Lopac | 1 microM |
| N-Succinyl-L-proline | Lopac | 1 microM |
| SB 205384 | Lopac | 1 microM |
| (-)-Scopolamine methyl bromide | Lopac | 1 microM |
| SU 4312 | Lopac | 1 microM |
| 1-(2-Methoxyphenyl)piperazine hydrochloride | Lopac | 1 microM |
| Sepiapterin | Lopac | 1 microM |
| Tiapride hydrochloride | Lopac | 1 microM |
| Trihexyphenidyl hydrochloride | Lopac | 1 microM |
| Terbutaline hemisulfate | Lopac | 1 microM |
| Tyrphostin AG 1478 | Lopac | 1 microM |
| BIX 01294 trihydrochloride hydrate | Lopac | 1 microM |
| (±)-alpha-Lipoic Acid | Lopac | 1 microM |
| Triprolidine hydrochloride | Lopac | 1 microM |
| SR 59230A oxalate | Lopac | 1 microM |
| PAPP | Lopac | 1 microM |
| R(-)-SCH-12679 maleate | Lopac | 1 microM |
| Taurine | Lopac | 1 microM |
| Theophylline | Lopac | 1 microM |
| 4-Hydroxyphenethylamine hydrochloride | Lopac | 1 microM |
| Tetrahydrozoline hydrochloride | Lopac | 1 microM |
| Terazosin hydrochloride | Lopac | 1 microM |
| DL-Thiorphan | Lopac | 1 microM |
| Tyrphostin AG 112 | Lopac | 1 microM |
| BRL 52537 hydrochloride | Lopac | 1 microM |
| Spiroxatrine | Lopac | 1 microM |
| (±)-SKF 38393, N-allyl-, hydrobromide | Lopac | 1 microM |
| Thiothixene hydrochloride | Lopac | 1 microM |
| (E)-4-amino-2-butenoic acid | Lopac | 1 microM |
| Triflupromazine hydrochloride | Lopac | 1 microM |

| | | |
|---|---|---|
| Tyrphostin AG 494 | Lopac | 1 microM |
| Tyrphostin AG 537 | Lopac | 1 microM |
| Tulobuterol hydrochloride | Lopac | 1 microM |
| Tyrphostin 1 | Lopac | 1 microM |
| SKF 89976A hydrochloride | Lopac | 1 microM |
| SR-95531 | Lopac | 1 microM |
| SDZ-205,557 hydrochloride | Lopac | 1 microM |
| Tolbutamide | Lopac | 1 microM |
| Tetradecylthioacetic acid | Lopac | 1 microM |
| Trimipramine maleate | Lopac | 1 microM |
| N-p-Tosyl-L-phenylalanine chloromethyl ketone | Lopac | 1 microM |
| Tyrphostin AG 555 | Lopac | 1 microM |
| Trazodone hydrochloride | Lopac | 1 microM |
| Tyrphostin 23 | Lopac | 1 microM |
| SIB 1757 | Lopac | 1 microM |
| (±)-6-Chloro-PB hydrobromide | Lopac | 1 microM |
| SB 206553 hydrochloride | Lopac | 1 microM |
| Tetraethylthiuram disulfide | Lopac | 1 microM |
| Trequinsin hydrochloride | Lopac | 1 microM |
| Tyrphostin AG 490 | Lopac | 1 microM |
| (6R)-5,6,7,8-Tetrahydro-L-biopterin hydrochloride | Lopac | 1 microM |
| Tyrphostin AG 698 | Lopac | 1 microM |
| Tyrphostin AG 34 | Lopac | 1 microM |
| Pifithrin-mu | Lopac | 1 microM |
| SIB 1893 | Lopac | 1 microM |
| L-Beta-threo-benzyl-aspartate | Lopac | 1 microM |
| SB 224289 hydrochloride | Lopac | 1 microM |
| TCPOBOP | Lopac | 1 microM |
| Tyrphostin AG 879 | Lopac | 1 microM |
| TTNPB | Lopac | 1 microM |
| Tyrphostin AG 527 | Lopac | 1 microM |

| | | |
|---|---|---|
| Tyrphostin AG 808 | Lopac | 1 microM |
| Triamcinolone | Lopac | 1 microM |
| Na-p-Tosyl-L-lysine chloromethyl ketone hydrochloride | Lopac | 1 microM |
| 1-(1-Naphthyl)piperazine hydrochloride | Lopac | 1 microM |
| Suramin sodium salt | Lopac | 1 microM |
| L-Tryptophan | Lopac | 1 microM |
| Tetraisopropyl pyrophosphoramide | Lopac | 1 microM |
| Tetraethylammonium chloride | Lopac | 1 microM |
| L-765,314 | Lopac | 1 microM |
| Theobromine | Lopac | 1 microM |
| Thio-NADP sodium | Lopac | 1 microM |
| S(-)-Timolol maleate | Lopac | 1 microM |
| Aprindine hydrochloride | Lopac | 1 microM |
| Ketanserin tartrate | Lopac | 1 microM |
| SQ 22536 | Lopac | 1 microM |
| Tranilast | Lopac | 1 microM |
| Tetramisole hydrochloride | Lopac | 1 microM |
| Tolazamide | Lopac | 1 microM |
| Triamterene | Lopac | 1 microM |
| (±)-Taxifolin | Lopac | 1 microM |
| Tyrphostin AG 835 | Lopac | 1 microM |
| N,N,N-trimethyl-1-(4-trans-stilbenoxy)-2-propylammonium iodide | Lopac | 1 microM |
| 1-[2-(Trifluoromethyl)phenyl]imidazole | Lopac | 1 microM |
| Taxol | Lopac | 1 microM |
| Tomoxetine | Lopac | 1 microM |
| Tamoxifen citrate | Lopac | 1 microM |
| Telenzepine dihydrochloride | Lopac | 1 microM |
| Uridine 5'-diphosphate sodium | Lopac | 1 microM |
| U-69593 | Lopac | 1 microM |
| U-99194A maleate | Lopac | 1 microM |
| Vincristine sulfate | Lopac | 1 microM |

| | | |
|---|---|---|
| WIN 62,577 | Lopac | 1 microM |
| Yohimbine hydrochloride | Lopac | 1 microM |
| Tetracaine hydrochloride | Lopac | 1 microM |
| NU6027 | Lopac | 1 microM |
| Terfenadine | Lopac | 1 microM |
| Thioperamide maleate | Lopac | 1 microM |
| U-74389G maleate | Lopac | 1 microM |
| UK 14,304 | Lopac | 1 microM |
| U0126 | Lopac | 1 microM |
| AMG 9810 | Lopac | 1 microM |
| S(-)-Willardiine | Lopac | 1 microM |
| YS-035 hydrochloride | Lopac | 1 microM |
| Tyrphostin 47 | Lopac | 1 microM |
| 3-Tropanyl-3,5-dichlorobenzoate | Lopac | 1 microM |
| Tropicamide | Lopac | 1 microM |
| (±)-Thalidomide | Lopac | 1 microM |
| Imiquimod | Lopac | 1 microM |
| U-62066 | Lopac | 1 microM |
| Vinblastine sulfate salt | Lopac | 1 microM |
| (±)-Vesamicol hydrochloride | Lopac | 1 microM |
| WAY-100635 maleate | Lopac | 1 microM |
| YC-1 | Lopac | 1 microM |
| Tyrphostin 51 | Lopac | 1 microM |
| Trifluoperazine dihydrochloride | Lopac | 1 microM |
| THIP hydrochloride | Lopac | 1 microM |
| R(+)-Terguride | Lopac | 1 microM |
| U-73122 | Lopac | 1 microM |
| S(-)-UH-301 hydrochloride | Lopac | 1 microM |
| (±)-Verapamil hydrochloride | Lopac | 1 microM |
| XK469 | Lopac | 1 microM |
| AC-55649 | Lopac | 1 microM |

| | | |
|---|---|---|
| Zaprinast | Lopac | 1 microM |
| PAC-1 | Lopac | 1 microM |
| D-609 potassium | Lopac | 1 microM |
| Trifluperidol hydrochloride | Lopac | 1 microM |
| Thio-L-citrulline | Lopac | 1 microM |
| SKF 95282 dimaleate | Lopac | 1 microM |
| R(+)-UH-301 hydrochloride | Lopac | 1 microM |
| A-134974 dihydrochloride hydrate | Lopac | 1 microM |
| Wortmannin from Penicillium funiculosum | Lopac | 1 microM |
| Xylazine hydrochloride | Lopac | 1 microM |
| Zonisamide sodium | Lopac | 1 microM |
| I-OMe-Tyrphostin AG 538 | Lopac | 1 microM |
| Thioridazine hydrochloride | Lopac | 1 microM |
| 3-Tropanyl-indole-3-carboxylate hydrochloride | Lopac | 1 microM |
| Tyrphostin A9 | Lopac | 1 microM |
| 4-Imidazoleacrylic acid | Lopac | 1 microM |
| CGP 57380 | Lopac | 1 microM |
| Vinpocetine | Lopac | 1 microM |
| ICI 63,137 | Lopac | 1 microM |
| SCH 58261 | Lopac | 1 microM |
| Caroverine hydrochloride | Lopac | 1 microM |
| Tyrphostin AG 538 | Lopac | 1 microM |
| Thapsigargin | Lopac | 1 microM |
| XCT790 | Lopac | 1 microM |
| TPMPA | Lopac | 1 microM |
| Urapidil hydrochloride | Lopac | 1 microM |
| (-)-trans-(1S,2S)-U-50488 hydrochloride | Lopac | 1 microM |
| Vancomycin hydrochloride from Streptomyces orientalis | Lopac | 1 microM |
| WB 64 | Lopac | 1 microM |
| Xylometazoline hydrochloride | Lopac | 1 microM |
| Olprinone hydrochloride | Lopac | 1 microM |

| | | |
|---|---|---|
| Trimethoprim | Lopac | 1 microM |
| Tyrphostin AG 126 | Lopac | 1 microM |
| 3-Tropanylindole-3-carboxylate methiodide | Lopac | 1 microM |
| U-75302 | Lopac | 1 microM |
| Urapidil, 5-Methyl- | Lopac | 1 microM |
| U-101958 maleate | Lopac | 1 microM |
| (±)-gamma-Vinyl GABA | Lopac | 1 microM |
| ( R)-(+)-WIN 55,212-2 mesylate | Lopac | 1 microM |
| Xanthine amine congener | Lopac | 1 microM |
| Zimelidine dihydrochloride | Lopac | 1 microM |
| ACETOVANILLON(RG) | Chromadex | 1 microM |
| ARBUTIN(RG) | Chromadex | 1 microM |
| BROMOFLAVONE; 6-(RG) | Chromadex | 1 microM |
| CORYDALINE, (+)-(RG) | Chromadex | 1 microM |
| DIHYDROXYACETOPHENONE,3,4-(RG) | Chromadex | 1 microM |
| DIMETHYLHYDROQUINONE, 2,3-(RG) | Chromadex | 1 microM |
| EPICATECHIN GALLATE, (-)-(P) | Chromadex | 1 microM |
| ACETOVERATRONE(RG) | Chromadex | 1 microM |
| ASCORBYL PALMITATE(USP)(RG) | Chromadex | 1 microM |
| CAFFEIC ACID PHENETHYL ESTER(CAPE)(P) | Chromadex | 1 microM |
| CORYNANTHINE(RAUHIMBINE)(RG) | Chromadex | 1 microM |
| DIHYDROXYBENZOIC ACID, 2,3-(RG) | Chromadex | 1 microM |
| DIMETHYLPHENOL,2,3-(SG) | Chromadex | 1 microM |
| EPIESTRIOL, 16-(RG) | Chromadex | 1 microM |
| FORMIC ACID BENZYLESTER(P) | Chromadex | 1 microM |
| ACETYL-3-ETHYLPYRAZINE, 2-(SG) | Chromadex | 1 microM |
| ATRACTYLOSIDE SODIUM SALT(RG) | Chromadex | 1 microM |
| CARMINIC ACID(RG) | Chromadex | 1 microM |
| CYANIDIN CHLORIDE(P) | Chromadex | 1 microM |
| DIHYDROXYFLAVONE, 7,8-(RG) | Chromadex | 1 microM |
| DIMETHYLUMBELLIFERONE,3,4-(RG) | Chromadex | 1 microM |

| | | |
|---|---|---|
| ESCULETIN(P) | Chromadex | 1 microM |
| FORSKOLIN(P) | Chromadex | 1 microM |
| ACETYL-6,7-DIMETHOXYCOUMARIN, 8-(RG) | Chromadex | 1 microM |
| BACCATIN III(P) | Chromadex | 1 microM |
| CASTANOSPERMINE(RG) | Chromadex | 1 microM |
| DEHYDROCOSTUS LACTONE(RG) | Chromadex | 1 microM |
| DIMETHOXY-2'-HYDROXYCHALCONE, 2,3-(RG) | Chromadex | 1 microM |
| DINITROBENZOIC ACID,3,5-(RG) | Chromadex | 1 microM |
| ESCULIN(ESCULETIN-6-GLUCOSIDE)(RG) | Chromadex | 1 microM |
| ADRENOSTERONE(RG) | Chromadex | 1 microM |
| BAICALEIN-5,6,7-TRIMETHYLETHER(RG) | Chromadex | 1 microM |
| CATHARANTHINE(RG) | Chromadex | 1 microM |
| DEHYDROPREGNENOLONE ACETATE, 16-(RG) | Chromadex | 1 microM |
| DIMETHOXYAPIGENINIDIN, 5,7-(SH) | Chromadex | 1 microM |
| DIOSMETIN(SH) | Chromadex | 1 microM |
| ESERINE SALICYLATE(RG) | Chromadex | 1 microM |
| GALANTHAMINE HYDROBROMIDE(RG) | Chromadex | 1 microM |
| AJMALICINE(RG) | Chromadex | 1 microM |
| BAICALEIN-7-METHYLETHER(RG) | Chromadex | 1 microM |
| DEOXYKAEMPFEROL, 5-(RG) | Chromadex | 1 microM |
| DIMETHOXYBENZOIC ACID,2,4-(RG) | Chromadex | 1 microM |
| DIPYRONE SODIUM(RG) | Chromadex | 1 microM |
| ESTRADIOL-17-VALERATE, 17B-(RG) | Chromadex | 1 microM |
| GENIPOSIDIC ACID(RG) | Chromadex | 1 microM |
| BAKUCHIOL(SH) | Chromadex | 1 microM |
| CHELIDONIC ACID(RG) | Chromadex | 1 microM |
| DIACETYL RHEIN(SH) | Chromadex | 1 microM |
| DIMETHOXYLUTEOLINIDIN, 5,7-(SH) | Chromadex | 1 microM |
| DITHIOERYTHREITOL(RG) | Chromadex | 1 microM |
| ETHISTERONE(RG) | Chromadex | 1 microM |
| ALPINETIN(SH) | Chromadex | 1 microM |

| | | |
|---|---|---|
| BENZYLALCOHOL(SG) | Chromadex | 1 microM |
| DIHYDROROBINETIN(RG) | Chromadex | 1 microM |
| DIMETHYLAMINOCINNAMALDEHYDE,4-(RG) | Chromadex | 1 microM |
| ETHYL-3-METHYLPYRAZINE,2-(RG) | Chromadex | 1 microM |
| GLAUCINE HBR(P) | Chromadex | 1 microM |
| AMINOCOUMARIN, 3-(RG) | Chromadex | 1 microM |
| CHRYSAZIN(RG) | Chromadex | 1 microM |
| DIMETHYLANTHRAQUINONE,2,3-(RG) | Chromadex | 1 microM |
| DOPA, L-(RG) | Chromadex | 1 microM |
| ETHYLENEDIAMINE(SG) | Chromadex | 1 microM |
| GLUCOTROPAEOLIN POTASSIUM SALT(SH) | Chromadex | 1 microM |
| AMMELIDE(P) | Chromadex | 1 microM |
| BERTEROIN(RG) | Chromadex | 1 microM |
| CONIINE, DL-(RG) | Chromadex | 1 microM |
| DIHYDROXY-4',6'-DIMETHOXYCHALCONE, 2',4-(RG) | Chromadex | 1 microM |
| DIMETHYLBENZALDEHYDE,3,4-(RG) | Chromadex | 1 microM |
| ECDYSONE, BETA-(P) | Chromadex | 1 microM |
| ETHYLQUINOL, 4-(RG) | Chromadex | 1 microM |
| AMMELINE(P) | Chromadex | 1 microM |
| BETULIN(P) | Chromadex | 1 microM |
| CORALYNE CHLORIDE, (-)-(RG) | Chromadex | 1 microM |
| DIHYDROXY-4-METHYLBENZOIC ACID,3,5-(RG) | Chromadex | 1 microM |
| DIMETHYLCAFFEIC ACID(RG) | Chromadex | 1 microM |
| ETHYLVANILLIN(SG) | Chromadex | 1 microM |
| GOSSYPETIN(RG) | Chromadex | 1 microM |
| ANISALDEHYDE, 4-(RG) | Chromadex | 1 microM |
| CORNIN(VERBENALIN)(RG) | Chromadex | 1 microM |
| DIHYDROXY-4-METHYLCOUMARIN, 5,7-(RG) | Chromadex | 1 microM |
| DIMETHYLFRAXETIN, O,O-(P) | Chromadex | 1 microM |
| EMBONIC ACID(RG) | Chromadex | 1 microM |
| FLAVONOL(3-HYDROXYFLAVONE)(RG) | Chromadex | 1 microM |

| | | |
|---|---|---|
| GUGGULSTERONES E&Z(MIX)(RG) | Chromadex | 1 microM |
| HARPAGOSIDE(P) | Chromadex | 1 microM |
| HECOGENIN(RG) | Chromadex | 1 microM |
| HELENIEN(ST) | Chromadex | 1 microM |
| HERNIARIN(RG) | Chromadex | 1 microM |
| HOMATROPINE(RG) | Chromadex | 1 microM |
| HORDENINE(FREE BASE)(P) | Chromadex | 1 microM |
| HUPERZINE A(P) | Chromadex | 1 microM |
| HYDROXY-5-METHYLFLAVONE, 7-(RG) | Chromadex | 1 microM |
| HYDROXYCITRIC ACID CALCIUM SALT, (-)-(P) | Chromadex | 1 microM |
| HYDROXYFLAVANONE, 7-(RG) | Chromadex | 1 microM |
| HYDROXYINDOLYL-3-ACETIC ACID, 5-(RG) | Chromadex | 1 microM |
| HYDROXYISOVANILLIC ACID,5-(RG) | Chromadex | 1 microM |
| HYDROXYPHENYL-2-BUTANONE,4-(RG) | Chromadex | 1 microM |
| HYDROXYPREGNENOLONE, 17a-(RG) | Chromadex | 1 microM |
| HYDROXYTROPINONE,6-(RG) | Chromadex | 1 microM |
| IBERVERIN(RG) | Chromadex | 1 microM |
| ISOASCORBIC ACID(RG) | Chromadex | 1 microM |
| ISOCINCHOMERONIC ACID(RG) | Chromadex | 1 microM |
| ISOCORYDINE HCL, (+)-(RG) | Chromadex | 1 microM |
| ISOFERULIC ACID(RG) | Chromadex | 1 microM |
| ISOQUERCETIN(P) | Chromadex | 1 microM |
| ISOSCOPOLETIN(P) | Chromadex | 1 microM |
| ISOSTEVIOL(RG) | Chromadex | 1 microM |
| ISOVANILLIN(RG) | Chromadex | 1 microM |
| JATRORRHIZINE(P) | Chromadex | 1 microM |
| JUSTALAKONIN(SH) | Chromadex | 1 microM |
| KAEMPFERIDE(SH) | Chromadex | 1 microM |
| KUROMANIN CHLORIDE(SH) | Chromadex | 1 microM |
| KUTKOSIDE(SH) | Chromadex | 1 microM |
| LAGOCHILINE (P) | Chromadex | 1 microM |

| | | |
|---|---|---|
| LANOSTEROL(RG) | Chromadex | 1 microM |
| LARICIRESINOL(P) | Chromadex | 1 microM |
| LAUDANOSINE, DL-(SH) | Chromadex | 1 microM |
| LOBARIC ACID(SH) | Chromadex | 1 microM |
| LOBELANIDINE HCL(P) | Chromadex | 1 microM |
| LUCIGENIN(RG) | Chromadex | 1 microM |
| LUPENONE(RG) | Chromadex | 1 microM |
| MADECASSOSIDE(P) | Chromadex | 1 microM |
| MALVIDIN CHLORIDE(P) | Chromadex | 1 microM |
| MANGOSTIN, ALPHA-(RG) | Chromadex | 1 microM |
| MATRINE(P) | Chromadex | 1 microM |
| METHACHOLINE BROMIDE(RG) | Chromadex | 1 microM |
| METHOXYFLAVANONE, 6-(RG) | Chromadex | 1 microM |
| METHOXYFLAVONOL, 4'-(RG) | Chromadex | 1 microM |
| METHOXYPHENOL, PARA-(SG) | Chromadex | 1 microM |
| METHYL-7-ETHOXYCOUMARIN, 4-(SG) | Chromadex | 1 microM |
| METHYL-7-METHOXYISOFLAVONE, 5-(P) | Chromadex | 1 microM |
| METHYLCATECHOL, 3-(RG) | Chromadex | 1 microM |
| METHYLDAPHNETIN, 4-(RG) | Chromadex | 1 microM |
| METHYLESCULETIN, 4-(RG) | Chromadex | 1 microM |
| METHYLGALLIC ACID, 3-O-(RG) | Chromadex | 1 microM |
| METHYLSYNEPHRINE(P) | Chromadex | 1 microM |
| MITRAGYNINE(SH) | Chromadex | 1 microM |
| MONOCROTALIN(CROTALIN)(RG) | Chromadex | 1 microM |
| NEOHESPERIDIN(SH) | Chromadex | 1 microM |
| NORHARMAN(RG) | Chromadex | 1 microM |
| NORNICOTINE, DL-(RG) | Chromadex | 1 microM |
| NORSTICTIC ACID(SH) | Chromadex | 1 microM |
| OLEUROPEIN(RG) | Chromadex | 1 microM |
| OSAJIN(RG) | Chromadex | 1 microM |
| OSTHOLE(P) | Chromadex | 1 microM |

| | | |
|---|---|---|
| OXOCAFESTOL, 16-(RG) | Chromadex | 1 microM |
| OXOSITOSTENONE, 6-(P) | Chromadex | 1 microM |
| OXYMATRINE(P) | Chromadex | 1 microM |
| OXYPEUCEDANIN HYDRATE (P) | Chromadex | 1 microM |
| PHENYLBUTYLISOTHIOCYANATE, 4-(RG) | Chromadex | 1 microM |
| PHENYLINDOL, ALPHA-(RG) | Chromadex | 1 microM |
| PHENYLMORPHOLINE, N-(RG) | Chromadex | 1 microM |
| PHYSCION(SH) | Chromadex | 1 microM |
| PICEIN (RG) | Chromadex | 1 microM |
| PILOCARPINE HCL(RG) | Chromadex | 1 microM |
| Piperlongumine | Chromadex | 1 microM |
| POLYDATIN(PICEID)(P) | Chromadex | 1 microM |
| POLYETHYLENE GLYCOL(RG) | Chromadex | 1 microM |
| POMIFERIN(RG) | Chromadex | 1 microM |
| PONGAMOL(RG) | Chromadex | 1 microM |
| PRATOL(SH) | Chromadex | 1 microM |
| PRAVASTATIN LACTONE(RG) | Chromadex | 1 microM |
| PREGNANETRIOL(RG) | Chromadex | 1 microM |
| PREGNENOLONE(RG) | Chromadex | 1 microM |
| PROPYL SULFIDE(SG) | Chromadex | 1 microM |
| PROPYL-3-METHYLPYRAZINE, 2-(SG) | Chromadex | 1 microM |
| PTEROSTILBENE(P) | Chromadex | 1 microM |
| PURPURIN(RG) | Chromadex | 1 microM |
| QUASSIN(SH) | Chromadex | 1 microM |
| REBAUDIOSIDE A (Rebiana)(FG) | Chromadex | 1 microM |
| RESERPINE(RG) | Chromadex | 1 microM |
| ROBINETIN(SH) | Chromadex | 1 microM |
| PHOSPHATIDYLCHOLINE(SEE LECITHIN PART# 12110)(RG) | Chromadex | 1 microM |
| SALICYL ALCOHOL(RG) | Chromadex | 1 microM |
| SALANNIN(P) | Chromadex | 1 microM |
| SITOSTERONE, BETA-(P) | Chromadex | 1 microM |

| | | |
|---|---|---|
| TIGLIC ACID(RG) | Chromadex | 1 microM |
| SALVIANOLIC ACID B(RG)(CALL) | Chromadex | 1 microM |
| STEVIOSIDE(RG) | Chromadex | 1 microM |
| VINPOCETINE (RG) | Chromadex | 1 microM |
| FLUTAMIDE(P) | Chromadex | 1 microM |
| SALICYLALDEHYDE(RG) | Chromadex | 1 microM |
| SOPHORICOSIDE(GENISTEIN-4'-GLUCOSIDE)(P) | Chromadex | 1 microM |
| SCOPARONE(RG) | Chromadex | 1 microM |
| STICTIC ACID(SH) | Chromadex | 1 microM |
| VULPINIC ACID(RG) | Chromadex | 1 microM |
| FUSIDIC ACID SODIUM SALT(RG) | Chromadex | 1 microM |
| SALICYLIC ACID BENZYLESTER(SG) | Chromadex | 1 microM |
| SPARTEINE(RG) | Chromadex | 1 microM |
| VERATRIC ACID(RG) | Chromadex | 1 microM |
| SCUTELLARIN(P) | Chromadex | 1 microM |
| WOGONIN(P) | Chromadex | 1 microM |
| GENTIOPICROSIDE(RG) | Chromadex | 1 microM |
| SILYCHRISTIN(P) | Chromadex | 1 microM |
| TETRAHYDROCORTICOSTERONE(RG) | Chromadex | 1 microM |
| YOHIMBIC ACID(RG) | Chromadex | 1 microM |
| PSEUDOPELLETIERINE(RG) | Chromadex | 1 microM |
| GUGGULSTERONES E&Z(MIX)(RG) | Chromadex | 1 microM |
| SESAMIN(RG) | Chromadex | 1 microM |
| SINIGRIN(SH) | Chromadex | 1 microM |
| TETRAHYDROPALMATINE, L-(RG) | Chromadex | 1 microM |
| PURPUROGALLIN(RG) | Chromadex | 1 microM |
| SALICYL ALCOHOL(RG) | Chromadex | 1 microM |
| TANGERETIN(RG) | Chromadex | 1 microM |
| SITOSTEROL, B- >80%(RG) | Chromadex | 1 microM |
| RESERPIC ACID HCL(RG) | Chromadex | 1 microM |
| Digoxin | Digitalis derivatives | 10 nanoM |

| | | |
|---|---|---|
| Digitoxin | Digitalis derivatives | 10 nanoM |
| Bufalin | Digitalis derivatives | 10 nanoM |
| Ouabain | Lopac | 10 nanoM |
| Oleandrin | Digitalis derivatives | 10 nanoM |
| Strophanthidin | Chromadex | 10 nanoM |
| ALOE EMODIN(SH) | Chromadex | 1 microM |
| BERBERINE SULFATE(RG) | Chromadex | 1 microM |
| BIXIN(P) | Chromadex | 1 microM |
| CEPHALOMANNINE(P) | Chromadex | 1 microM |
| CHELIDONINE, (+)-(P) | Chromadex | 1 microM |
| DIHYDROXY-1,4-NAPHTHOQUINONE,5,8-(RG) | Chromadex | 1 microM |
| DOCETAXEL(P) | Chromadex | 1 microM |
| EMBELIN(RG) | Chromadex | 1 microM |
| LANATOSIDE B(RG) | Chromadex | 1 microM |
| MEVINOLIN(SH) | Chromadex | 1 microM |
| PIPERLONGUMINE(RG) | Chromadex | 1 microM |
| SECURININE(RG) | Chromadex | 1 microM |
| SHIKONIN(SH) | Chromadex | 1 microM |
| STROPHANTHIDOL(RG) | Chromadex | 1 microM |
| STROPHANTHIN, g-(RG) | Chromadex | 1 microM |
| TETRANDRINE, (S,S)-(+)-(RG) | Chromadex | 1 microM |
| TOMATINE(RG) | Chromadex | 1 microM |
| XAV939 | Signaling compounds | 5 microM |
| celecoxib | Signaling compounds | 50 microM |
| PHA665752 | Signaling compounds | 1 microM |
| DAPT | Signaling compounds | 10 microM |
| Cyclopamine hydrate | Signaling compounds | 10 microM |
| LY294002 | Signaling compounds | 10 microM |
| SB431542 | Signaling compounds | 10 microM |
| DKK1 | Signaling compounds | 100 ng/mL |
| sFRP1 | Signaling compounds | 200 ng/mL |

| | | |
|---|---|---|
| HGF | Signaling compounds | 50 ng/mL |
| EGF | Signaling compounds | 20 ng/mL |
| Iressa | Signaling compounds | 1 microM |
| Reserpine | Signaling compounds | 10 microM |
| HGFAb | Signaling compounds | 10 microg/mL |
| AMD3100 | Signaling compounds | 10 microM |
| MK8033 | Signaling compounds | 10 microM |
